# Resveratrol Ameliorates Lipopolysaccharide-Induced Sudden Sensorineural Hearing Loss in *In Vitro* Model through Multitarget Antiapoptotic Mechanism Based on Network Pharmacology and Molecular Docking

**DOI:** 10.1155/2022/6404588

**Published:** 2022-05-19

**Authors:** Shiming Ye, Jing Liu, Qi Dong, Xinxin Wang, Wandong She

**Affiliations:** ^1^Department of Otolaryngology-Head and Neck Surgery, Nanjing Drum Tower Hospital Clinical College of Traditional Chinese and Western Medicine, Nanjing University of Chinese Medicine, Nanjing 210008, China; ^2^Department of Otolaryngology, Yizheng People's Hospital, Yangzhou 211400, Jiangsu, China; ^3^Department of Otolaryngology Head and Neck Surgery, Nanjing Drum Tower Hospital, The Affiliated Hospital of Nanjing University Medical School, Nanjing 210008, China; ^4^Department of Otolaryngology-Head and Neck Surgery, Nanjing Drum Tower Hospital, Medical College of Southeast University, Nanjing 210008, China

## Abstract

**Objective:**

To explore the effects of resveratrol (RSV) on hair cell apoptosis caused by sudden sensorineural hearing loss (SSNHL) and its effect on lipopolysaccharide-induced apoptosis of HEI-OC1 cells.

**Methods:**

We used the network pharmacology method to screen molecules related to RSV for the treatment of SSNHL and analyzed these molecules and their enriched biological processes and signaling pathways through Kyoto Encyclopedia of Genes and Genomes (KEGG) and Gene Ontology (GO) analysis. We selected hub genes related to apoptosis using protein-protein interaction (PPI) analysis for *in vitro* and molecular docking verification.

**Results:**

Eighty overlapping genes were identified as potential targets for RSV treatment of SSNHL. Further GO analysis showed that the biological processes were mainly related to toxicity, cell proliferation, and lipopolysaccharide reactions. KEGG analysis showed that the AGE-RAGE signaling pathway in diabetic complications, Kaposi's sarcoma-associated herpesvirus infection, FoxO signaling pathway, PI3K-Akt signaling pathway, and other inflammatory signaling pathways were concentrated. *AKT1, STAT3, JUN, TNF, TP53, MAPK3, CASP3*, and *VEGFA* were screened as HUB genes using PPI analysis. The apoptosis-related proteins TNF, CASP3, AKT1, and TP53 were selected for *in vitro* experiments, which showed that mRNA was significantly different before and after RSV intervention, confirming that the corresponding protein receptors could bind well with RSV.

**Conclusion:**

RSV mainly affects the prognosis of SSNHL through anti-inflammatory effects and may improve hair cell apoptosis caused by inflammatory factors through multitargeted interventions involving TNF, CASP3, AKT1, and TP53.

## 1. Introduction

Although sudden sensorineural hearing loss (SSNHL) is an acute sensorineural hearing loss of unknown etiology, our recent studies have shown that inflammatory factors may represent a common pathological basis for SSNHL caused by various elements [1]. Apoptosis is considered to be the main pathogenic manifestation of SSNHL [2], along with age-related deafness [3] and autoimmune inner-ear disease [4]. Apoptosis of cochlear sensory hair cells has been observed in SSNHL animal models, *in vitro* models, and lipopolysaccharide (LPS) models of the middle ear [5–7]. At present, SSNHL still involves glucocorticoid-based comprehensive treatment [8, 9], but certain treatments are still ineffective even if the dosage of glucocorticoids is timely and sufficient [10]. Under the traditional concept of “one disease, one gene, one drug,” no specific treatment has been found. Therefore, multitargeted natural products involving a broad range of pharmacological activities are most likely to show potential superiority [11].

Resveratrol (RSV) is a polyphenolic organic compound widely found in a variety of herbaceous plants and is a bioactive component of wine and grape juice [12]. It has extensive anti-inflammatory, antioxidant, neuroprotective, and cardiovascular protective effects [13]. Its molecular weight (MW = 228.25) is low and has good blood-brain barrier (BBB = −1) permeability, which obeys Lipinski's five laws, including a MW between 180 and 500 Da, an octanol-water partition coefficient (Alog *P*=3.01) below 5, the number of possible receptors (Hacc = 3) < 10, and hydrogen bond donors (Hdon = 3) < 5 [14]. We speculated that RSV might also play a therapeutic role in sensorineural hearing loss (SHL) through the blood-labyrinth barrier. Many studies have found that RSV exhibits good outcomes in the treatment of SHL, such as age-related deafness [3], cisplatin-induced hearing loss [15], and noise-induced hearing loss [16]. However, RSV has not been reported in SSNHL.

**Figure 1:**
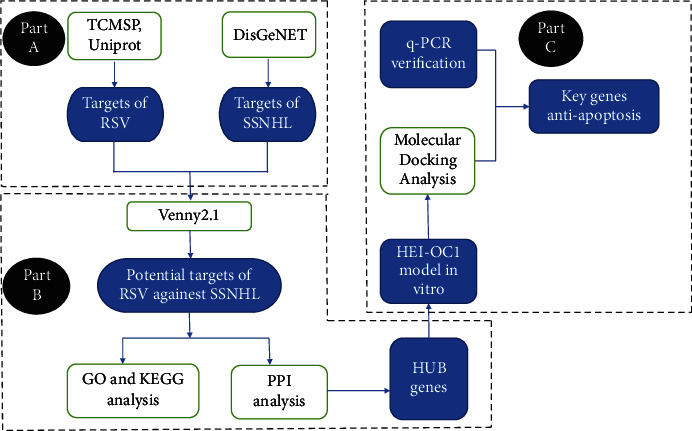
Flowchart of analysis. (a) Target search using TCMSP, Uniprot, and DisGeNET. (b) Target analysis using Venny2.1, GO, and KEGG with PPI. (c) Key target verification in HEI-OC1 cells.

In the present study, we used the network pharmacology method to screen RSV- and SSNHL-related molecules and analyzed these molecules and their enriched biological processes and signaling pathways using Kyoto Encyclopedia of Genes and Genomes (KEGG) and Gene Ontology (GO) analysis. Moreover, we selected hub genes related to apoptosis through protein-protein interaction (PPI) analysis for molecular docking verification and further verified the results in an *in vitro* model of LPS-induced acute hearing loss. Our results suggested that RSV plays a protective role in cochlear cells by regulating key factors involved in the apoptotic pathway. The current study lays a foundation for future *in vivo* validation and clinical application to improve the prognosis of patients with refractory SSNHL. The flowchart of such analysis is presented in Figure 1.

## 2. Materials and Methods

### 2.1. RSV-Related Target Genes Screening

The Traditional Chinese Medicine System Pharmacology (TCMSP) database is based on the framework of Traditional Chinese Medicine System Pharmacology [17]. We used the TCMSP (http://tcmspw.com/tcmsp.php) database to search for RSV-related targets and used the UniProt [18] (https://www.uniprot.org) database to convert the obtained target proteins into the corresponding genes (the species was defined as human). All database searches were conducted in March 2021.

### 2.2. Acquisition of Target Genes Related to SSNHL

We used the DisGeNET [19] (http://www.disgenet.org) database to search the pathogenesis of SSNHL-related target genes. The DisGeNET database is a comprehensive platform integrating and specifying disease-related genes and provides variation data from the scientific literature and multiple sources.

### 2.3. Potential Drug-Disease Target Gene Screening

We used the online tool Venny2.1 to analyze intersecting genes (https://bioinfogp.cnb.csic.es/tools/venny) [20], identify potential RSV-SSNHL target genes, and save the Venn diagram.

### 2.4. GO Function Enrichment and KEGG Analysis

The RSV-SSNHL cross genes whose names were modified to formal gene symbols were introduced into David 6.8 [21] (https://david.ncifcrf.gov/home.jsp) and KEGG pathway [22] (https://www.genome.jp/kegg/pathway.html) databases. The species was defined as “human,” and the exclusion criteria were set as *p* value < 0.05 and *q* value < 0.05.

### 2.5. PPI Analysis of Potential Target Genes and Screening of Hub Genes

Potential drug-disease target genes were imported into the online database STRING [23] (https://string-db.org/) to conduct PPI analysis (selecting high confidence 0.7), and the results were input into Cytoscape software. We selected the top-eight degrees as hub genes. Hub genes related to apoptosis were selected for further *in vitro* verification.

### 2.6. Cell Culture and Treatments

#### 2.6.1. CCK-8 Analysis

HEI-OC1 cells, donated by Professor Chai Renjie's Laboratory (Medical College of Southeast University, Nanjing, China), were cultured in DMEM containing 10% fetal bovine serum (FBS) and 100 U/mL penicillin. Cells were seeded into 96-well microplates at a density of 5 × 10^3^ cells/well for 24 h. The cells were then divided into three groups. The first group was cultured with various concentrations (0, 2, 5, 10, 25, and 50 *μ*g/mL) of LPS (L2630, Sigma) in a high-glucose DMEM culture medium for 24 h. A CCK-8 kit (A311-01/02, Vazyme, Nanjing, China) was used to determine the optimal concentration. The second group (RSV + LPS group) was pretreated with different concentrations (0, 5, 10, 20, and 40 *μ*mol) of RSV (HY-16561, MCE) for 12 h and then treated with the optimum concentration of LPS for 24 h. The third group was a solvent control cohort. All experiments were repeated three times.

#### 2.6.2. Apoptosis Analysis Using FITC/PI

Cells were collected, digested with EDTA-free trypsin, and washed twice with precooled PBS. The cells were gently blown into a single-cell suspension with 100 *μ*L of 1 × binding buffer. Annexin V-FITC (5 *μ*L of) and 5 *μ*L propidium iodide (PI) staining solutions were added to the binding buffer. Cells were incubated in the dark for 10 min at room temperature, gently mixed with 400 *μ*L of 1 × binding buffer, and subjected to flow cytometry within 1 h. The total apoptotic rate is calculated as follows: Q2+Q3.

#### 2.6.3. RT-qPCR Analysis

To examine differentially expressed mRNAs in HEI-OC1 cells in the different treatment groups, total RNA was extracted using TRIzol (R401-01, Vazyme) and cDNA was generated using the HiScriptII 1st Strand cDNA Synthesis Kit (R211-01/02,Vazyme). RT-qPCR was performed using ChamQ SYBR qPCR Master Mix (Q311-02/03, Vazyme), according to the manufacturer's instructions. PCR primers were synthesized by TSINGKE Biotechnology Co., Ltd. (Beijing, China) and were purified by oligonucleotide purification cartridge (OPC). The results are shown in Table 1. The samples underwent initial denaturation at 95°C for 30 s, and then 40 cycles were performed at 95°C for 10 s and 60°C for 30 s. Finally, melting curves were obtained under the conditions of 95°C for 15 s, 60°C for 60 s, and 95°C for 15 s, repeated three times for each gene. Changes in expression were calculated using the 2^−ΔΔCT^ method.

#### 2.6.4. Molecular Docking Analysis

Molecular docking can be used to model the interactions between a small molecule and protein structures at the atomic level. Molecular docking analysis was used to predict the binding of apoptosis-related proteins to RSV. We obtained the RSV three-dimensional structure from the PubChem database (https://pubchem.ncbi.nlm.nih.gov/) and the three-dimensional structures of apoptosis-related hub protein receptors from the RCSB Protein Data Bank (PDB) database (http://www.rcsb.org/). Molecular docking simulations of the target protein receptor and RSV were performed using AutoDock Tool (v.1.5.6) and AutoDock Vina 1.1.2 (Molecular Graphics Laboratory, Scripps Institute, 2011) and displayed using the PyMOL molecular graphics system (v.2.4.0, Schrödinger, LLC) [24].

### 2.7. Statistical Analysis

Data are presented as means ± standard deviation (SD). All statistical analyses were performed using Microsoft Excel and GraphPad Prism, v.7. Two-tailed unpaired Student's *t*-test was used for analysis involving two groups. One-way analysis of variance (ANOVA) was used among multiple groups (> two groups), *p* values < 0.05 were considered statistically significant.

## 3. Results

### 3.1. Screening Results of RSV Target Genes

A total of 151 RSV target proteins were obtained from TCMSP and back-translated into their corresponding genes using UniProt. The species selected was humans. The results are presented in Table S1.

### 3.2. Screening Results of Target Genes Related to SSNHL

A total of 2,342 corresponding target genes were obtained from the DisGeNET database by inputting the disease name of sudden sensorineural hearing loss. The results are presented in Table S2.

### 3.3. Potential Drug-Disease Target Gene Screening Results

**Figure 2:**
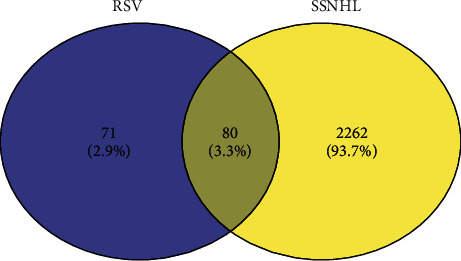
Venny analysis of RSV-SSNHL potential target genes. Eighty RSV-SSNHL cross genes were found by this analysis.

We obtained a total of 80 drug-disease cross genes using the online tool Venny2.1 (Figure 2).

### 3.4. GO Functional Enrichment and KEGG Analysis Results

**Figure 3:**
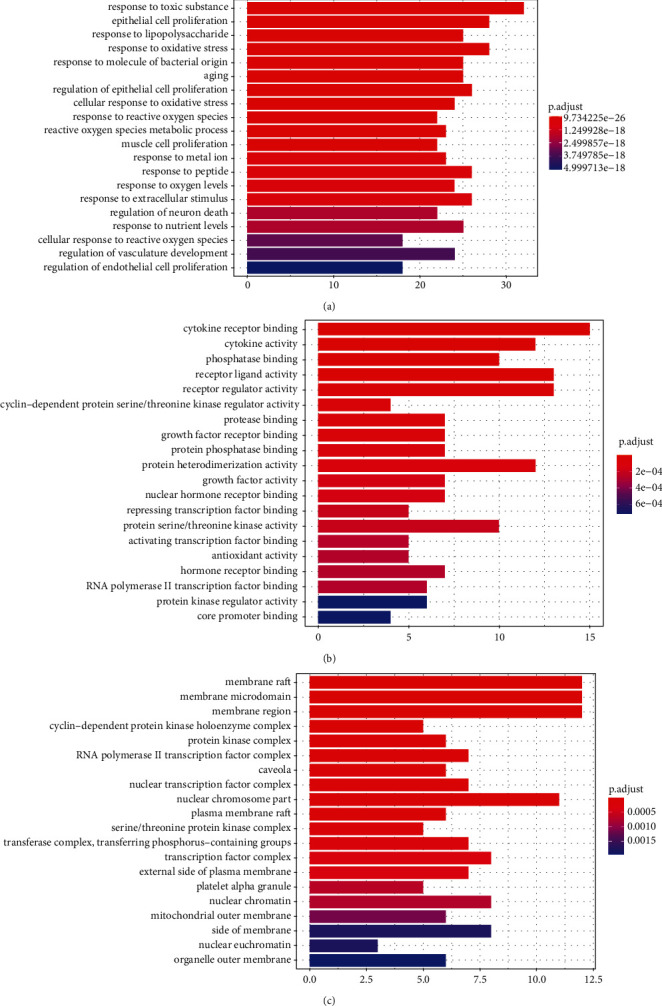
GO enrichment analysis of potential targets. (a) Molecular function enrichment analysis. (b) Cell composition enrichment analysis. (c) Bioprocess enrichment analysis. *p* value gradually increases as the color changes from dark red to blue.

GO analysis showed that toxic reactions, cell proliferation, response to LPS, oxidative stress, inflammation, and aging were mainly involved in cytokine receptor, receptor ligand activity, receptor regulator activity, cytokine activity, and protein dimerization activity. The cell composition mainly included membrane rafts, membrane microdomains, membrane regions, nuclear chromosomes, and transcription factor complexes (Figure 3).

**Figure 4:**
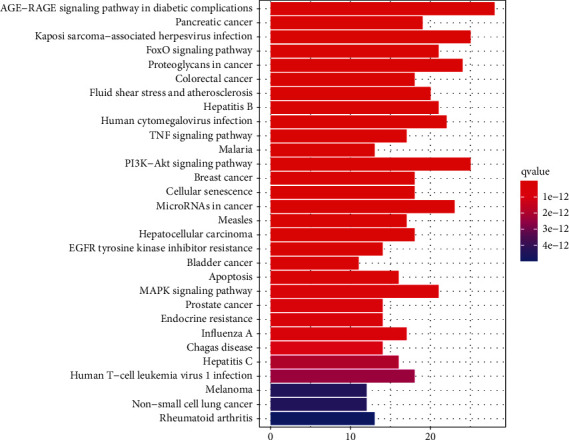
KEGG enrichment analysis of potential targets. q value is the adjusted *p* value, and q value gradually increases as the colors change from dark red to blue.

KEGG analysis indicated that 80 potential target genes were enriched involving 92 signaling pathways, of which the 10 most important pathways included the AGE-RAGE signaling pathway in diabetic complications, Kaposi's sarcoma-associated herpesvirus infection, FoxO signaling pathway, PI3K-Akt signaling pathway, proteoglycan in cancer, small molecule ribonucleic acids in cancer, human cytomegalovirus infection, hepatitis B, MAPK signaling pathway, fluid shear stress, and atherosclerosis (Figure 4).

### 3.5. PPI Analysis of Potential Target Genes Related to Apoptosis of Top Ten Genes

**Figure 5:**
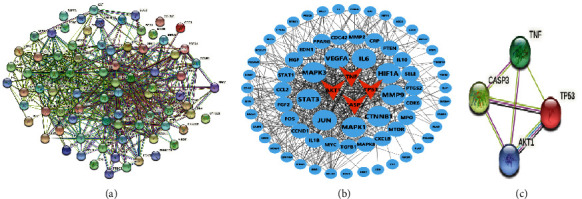
PPI analysis and target genes antiapoptosis screening results. (a) The string analysis of all genes resveratrol-SSNHL. (b) Cytoscape mapping results by degree values and apoptosis-related genes. (c) The relationship of four apoptosis-related genes based on STRING database analysis, edges represent protein-protein associations: green lines mean textmining; wine red lines mean experimentally determined; pale yellow lines mean from curated databases; black lines mean co-expression.

We used the online STRING database to analyze differential genes PPI and imported the results into Cytoscape software to analyze 2,416 related nodes (Figure 5(a)). According to the number of nodes ≥70, *AKT1, STAT3, JUN, TNF, TP53, MAPK3, CASP3*, and *VEGFA* were screened (Table S3). Among these hub genes, *TNF, CASP3, AKT1*, and *TP53* are involved in the apoptosis pathway both outside and inside the cell. We speculated that this might play a protective role in hair cells by regulating the apoptosis pathway involving TNF, CASP3, AKT1, and TP53 (Figure 5(b)). We used the STRING database to further analyze the relationship among TNF, CASP3, AKT1, and TP53 (Figure 5(c)).

#### 3.5.1. CCK-8 Analysis Results

**Figure 6:**
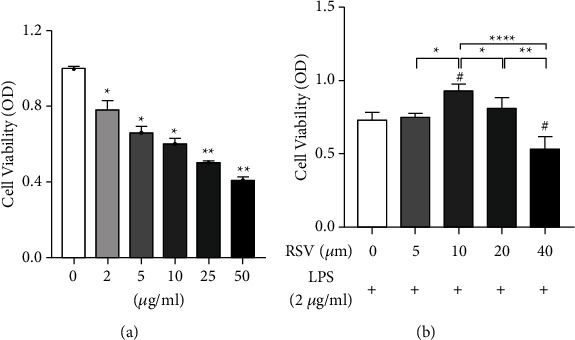
CCK-8 analysis results. (a) CCK-8 analysis results for HEI-OC1 cells exposed to different concentrations of LPS. All concentrations of LPS induced significant apoptosis in HEI-OC1 cells versus the control group (^*∗*^*p* < 0.05 or ^*∗∗*^*p* < 0.01). (b) Protective effects of RSV against LPS-induced apoptosis in HEI-OC1 cells. #*p* < 0.05 versus the control group (0 *μ*mol of RSV). 10 *μ*mol RSV administration showed significant protection versus the control group (0 *μ*mol of RSV). 5 and 20 *μ*mol of RSV did not exhibit a significant protective effect. 40 *μ*mol of RSV even reduced the activity compared to that in the control group (^*∗*^*p* < 0.05, ^*∗∗*^*p* < 0.01, or ^*∗∗∗∗*^*p* < 0.001).

CCK-8 screening showed that LPS (2 *μ*g/mL) could induce apoptosis in HEI-OC1 cells after 24 h of treatment (Figure 6(a)). Therefore, we chose a concentration of 2 *µ*g/mL for subsequent experiments. We found that 10 *μ*mol RSV exerted a significant protective effect against LPS-induced apoptosis in HEI-OC1 cells in a dose-dependent manner (Figure 6(b)).

#### 3.5.2. FITC/PI Analysis Results

**Figure 7:**
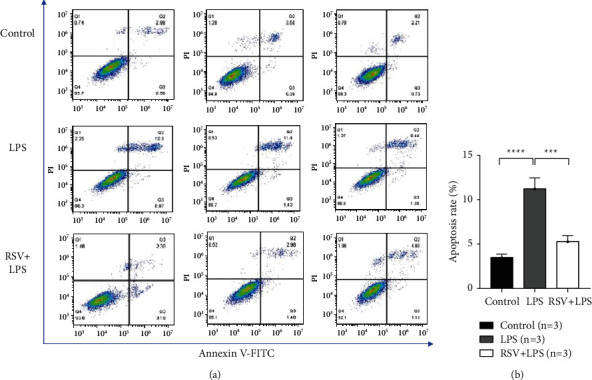
Results of FITC/PI analysis. (a) Examples of FITC/PI flow cytometric analysis results of three groups. (b) Statistical analysis results of FITC/PI flow cytometry apoptosis rates among groups. ^*∗∗∗∗*^*p* < 0.0001 versus the control group and ^*∗∗∗*^*p* < 0.001 versus the RSV + LPS group.

FITC/PI analysis showed that there was a significant difference in apoptotic rates in HEI-OC1 cells among the different groups. Compared with the control group, the apoptosis rate in the LPS intervention group significantly increased, whereas there was no significant difference in the apoptosis rate in the RSV + LPS intervention group (Figure 7).

#### 3.5.3. RT-qPCR Analysis Results

**Figure 8:**
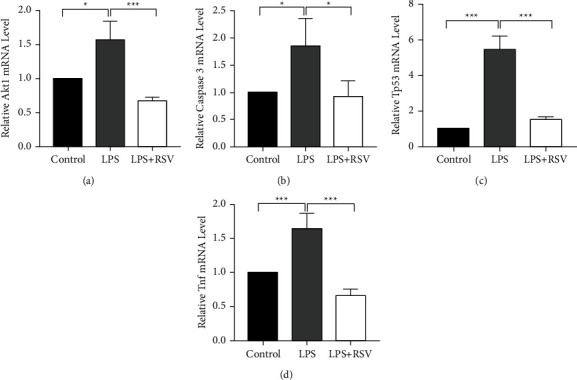
mRNA expression of apoptosis-related genes among groups. (a) The AKT1 mRNA expression. (b) The caspase-3 mRNA expression. (c) The TP53 mRNA expression. (d) The TNF mRNA expression. Compared to the control group, the AKT1, TNF, caspase-3, and TP53 levels in the LPS group significantly increased (^*∗*^*p* < 0.05 or ^*∗∗∗*^*p* < 0.001) while no significant changes were observed in the RSV + LPS group (*p* > 0.05).

RT-qPCR results showed that the mRNA expression levels of apoptosis-related genes in the different groups were statistically different: the AKT1, TNF, caspase3, and TP53 levels in the LPS group were higher than those in the control group, and this difference was statistically significant, while there was no significant difference in the RSV + LPS group (Figure 8).

#### 3.5.4. Molecular Docking Results

**Figure 9:**
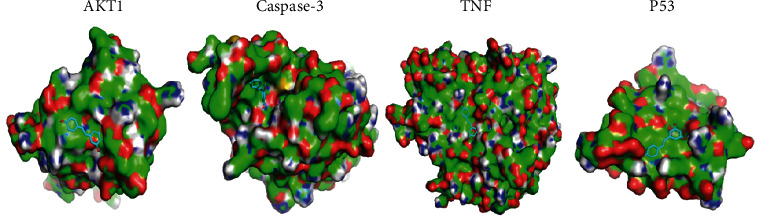
Diagram of structural interactions between resveratrol and apoptosis-related key receptors.

We obtained the three-dimensional structure of RSV from the PubChem database and the three-dimensional structure of apoptosis-related protein target receptors from the RCSB PDB database. Molecular docking potential simulation of target proteins and RSV was generated using AutoDock Tool and AutoDock Vina software. Finally, combinations of the target and equivalent component were verified through molecular docking, and the combination relationship between the target and equivalent component was established using the PyMOL molecular graphics system. We designated TNF-RSV, caspase-3-RSV, AKT1-RSV, and P53-RSV for verification. Among the four docking simulations, the minimum affinity was −7.8 kcal/mol, −7.4 kcal/mol, −6.2 kcal/mol, and −5.5 kcal/mol (Table 2). The grid centers are shown in Table 2, and distances to the best mode were 0.000 rmsd l.b. and 0.000 rmsd u.b. (Figure 9).

## 4. Discussion

The etiology of SSNHL remains unclear, which may be related to hair cell apoptosis and damage caused by inflammatory responses, viral infection, microcirculation disturbance, and other factors, because inflammation can result in microvascular damage [25], atherosclerosis [26], and cochlear immune responses [27]; *in vivo* experiments have shown that LPS-stimulated guinea pigs have severe cochlear microcirculation disturbance [28]. Therefore, inflammation is considered to be an important cause of SSNHL. We identified 151 potential RSV targets from TCMSP and 2,342 SSNHL-related genes from the DisGeNET database. Venny analysis identified 80 overlapping genes as potential targets for RSV treatment of SSNHL. Furthermore, GO analysis revealed that the potential targets are mainly involved in toxic reactions, cell proliferation, and LPS reactions. The molecular functions involved were mainly cytokine receptors and cellular components, including membrane rafts. KEGG analysis also focused on the AGE-RAGE signaling pathway in diabetic complications, Kaposi's sarcoma-associated herpesvirus infection, FoxO signaling pathway, PI3K-AKT signaling pathway, and other inflammatory and apoptosis-related signaling pathways. To further understand relationships among potential targets, we screened *AKT1, STAT3, JUN, TNF, TP53, MAPK3, CASP3*, and *VEGFA* as hub genes using PPI analysis. Among these hub genes, *TNF, CASP3, AKT1*, and *TP53* are involved in intracellular or extracellular apoptotic pathways. We verified that RSV could improve LPS-induced HEI-OC1 cell apoptosis by TNF, CASP3, AKT1, and TP53 multitargeted intervention *in vitro*. Molecular docking studies also identified that these central targets exhibited good affinity for RSV.

Glucocorticoids are currently recognized as the primary treatment for various types of SSNHL. Studies have shown that dexamethasone can play a protective role in TNF-*α*-induced apoptosis by activating signaling pathways such as PI3K/AKT and NF-kB [29] and revealed the protective effect of tunicamycin in HEI-OC1 cells by inhibiting endoplasmic reticulum stress [30]. However, certain patients remain insensitive to hormone therapy, which may play a beneficial role through multitargeted intervention involving traditional Chinese medicine.

RSV is a natural polyphenol with a stilbene skeleton that has many beneficial properties such as anti-inflammatory, antioxidant, and neuroprotective activities [13]. Studies have shown that RSV can be used to treat neurodegenerative (ND) diseases through multitargeted treatment [31]. In this study, we selected *TNF, CASP3, AKT1,* and *TP53* as hub genes related to apoptosis by PPI analysis. TNF-*α* is closely related to the occurrence and development of nervous disorders. Previous studies have shown that TNF-*α* inhibits glucocorticoid receptor function, *TNF-α* mutant mice are prone to high-frequency hearing loss during early development stages [32], and targeted silencing of TNF-*α* is beneficial to prevent NIHL [33]. Exogenous and endogenous pathways focus on the activation of executors such as caspase-3 and ultimately the physical execution of apoptotic cell death [34]. Studies have shown that inhibiting caspases can prevent or delay hair cell death induced by noise or aminoglycosides [35]. In a hypoxia-induced SSNHL *in vitro* model, the level of cleaved-caspase-3 significantly increased [2]. AKT1 promotes cell proliferation by regulating cyclins and inhibits apoptosis by p53 [36]. Studies have also shown that age-related cochlear hair cell apoptosis is related to miR-34a/SIRT1/p53 signal transduction, which may represent a potential target for the treatment of age-related hearing loss [3]. Our results showed that RSV regulates *TNF, CASP3, AKT1*, and *TP53* mRNA levels and may play a protective role in LPS-induced apoptosis of hair cells through multitarget regulation. Molecular docking further confirmed the good docking performance of the target protein structures with RSV.

We may have chosen a relatively single database in the early stage of network pharmacological analysis, but TCMSP and DisGeNET databases selected in this study are the most representative databases. Among them, TCMSP contains 29,384 species of 499 Chinese herbal medicines, 3,311 targets, and 837 related diseases registered with the Chinese Pharmacopoeia [17]. The DisGeNET release in 2019 covers over 24,000 diseases and features, 17,000 genes, and 117,000 genomic variations, covering the full spectrum of human diseases and normal and abnormal features [19]. In the process of *in vitro* verification, we found that apoptosis of HEI-OC1 cells increased after LPS treatment, and flow cytometric analysis showed that the apoptosis rate was significantly higher than that of the control group. The apoptosis rate of the RSV + LPS group decreased, and there was no significant difference compared to that of the control cohort. We verified binding involving target proteins corresponding to key genes and RSV through molecular docking studies. Moreover, we found that TNF had the lowest minimum binding energy (-7.8 kcal/mol) among the four key apoptosis-related proteins, which further verified the close relationship between the pathogenesis of SSNHL and inflammation. Consistent with previous studies [1, 37–39], we speculate that RSV might play a protective role in hair cell apoptosis mainly through anti-inflammatory effects.

## 5. Conclusion

In summary, RSV is expected to be a safe and effective multitarget drug treatment for SSNHL therapy. Our network pharmacological analysis involving RSV predicted that the therapeutic effect of RSV is mediated by the regulation of apoptosis-related pathways involving TNF, CASP3, AKT1, and TP53. It will be necessary to explore the main mechanisms of RSV action through further *in vivo* verification experiments.

## Figures and Tables

**Table 1:** Primer list for mRNA expression of apoptosis-related genes.

Primers for mRNA expression of apoptosis-related genes (5′-3′)		
Primers	Forward	Reverse
Caspase-3	GAAACTCTTCATCATTCAGGCC	GCGAGTGAGAATGTGCATAAAT
Tnf	ATGTCTCAGCCTCTTCTCATTC	GCTTGTCACTCGAATTTTGAGA
Akt1	TGCACAAACGAGGGGAATATAT	CGTTCCTTGTAGCCAATAAAGG
Tp53	TGGAAGGAAATTTGTATCCCGA	GTGGATGGTGGTATACTCAGAG
Gapdh	AGGTCGGTGTGAACGGATTTG	TGTAGACCATGTAGTTGAGGTCA

**Table 2:** The minimum binding energy of resveratrol and apoptosis-related key receptors.

Drug	Target	The minimum binding energy (kcal/mol)	Grid center (X/Y/Z)
Resveratrol	TNF	−7.8	20.083/49.892/39.738
	Caspase-3	−7.4	26.71/22.591/37.128
	AKT1	−6.2	21.62/14.471/9.976
	P53	−5.5	52.103/−12.611/45.156

## Data Availability

Data can be obtained from authors upon reasonable request.

## Supplementary Materials

Supplementary Materials
*Table S1.* Targets of resveratrol from the TCMSP database (151 resveratrol target proteins were obtained and transformed into corresponding differential genes). *Table S2*. Targets of SSNHL from the DisGeNET database (2342 corresponding target genes were obtained). *Table S3*. RSV-SSNHL potential targets and Cytoscape analysis results (a total of 2416 nodes were obtained by PPI analysis, and the number of nodes was selected as greater than or equal to 70).
