# Supplementary material for: Resveratrol Ameliorates Lipopolysaccharide-Induced Sudden Sensorineural Hearing Loss in In Vitro Model through Multitarget Antiapoptotic Mechanism Based on Network Pharmacology and Molecular Docking
Source: Evid Based Complement Alternat Med. 2022 May 19;2022:6404588. doi: 10.1155/2022/6404588 (PMC9135530; doi:10.1155/2022/6404588)
Supplement: Supplementary Materials — Table S1. Targets of resveratrol from the TCMSP database (151 resveratrol target proteins were obtained and transformed into corresponding differential genes). Table S2. Targets of SSNHL from the DisGeNET database (2342 corresponding target genes were obtained). Table S3. RSV-SSNHL potential targets and Cytoscape analysis results (a total of 2416 nodes were obtained by PPI analysis, and the number of nodes was selected as greater than or equal to 70). [file 6404588.f1.zip › 6404588.f1/Table S1 Resveratrol targets.docx]

**Table S1 Targets of Resveratrol from the TCMSP database**

| **Drug** | **Protein name** | **Gene name** |
| --- | --- | --- |
| Resveratrol | Prostaglandin G/H synthase 1 | PTGS1 |
|  | Prostaglandin G/H synthase 2 | PTGS2 |
|  | Amine oxidase [flavin-containing] B | MAOB |
|  | Nuclear receptor coactivator 2 | NCOA2 |
|  | Carbonic anhydrase II | CA2 |
|  | Transcription factor p65 | RELA |
|  | Signal transducer and activator of transcription 3 | STAT3 |
|  | RAC-alpha serine/threonine-protein kinase | AKT1 |
|  | Vascular endothelial growth factor A | VEGFA |
|  | G1/S-specific cyclin-D1 | CCND1 |
|  | Apoptosis regulator Bcl-2 | BCL2 |
|  | Bcl-2-like protein 1 | BCL2L1 |
|  | Proto-oncogene c-Fos | FOS |
|  | Cyclin-dependent kinase inhibitor 1 | CDKN1A |
|  | Eukaryotic translation initiation factor 6 | EIF6 |
|  | Apoptosis regulator BAX | BAX |
|  | Caspase-9 | CASP9 |
|  | Urokinase-type plasminogen activator | PLAU |
|  | 72 kDa type IV collagenase | MMP2 |
|  | Matrix metalloproteinase-9 | MMP9 |
|  | Mitogen-activated protein kinase 3 | MAPK3 |
|  | Mitogen-activated protein kinase 1 | MAPK1 |
|  | Interleukin-10 | IL10 |
|  | Cell division protein kinase 4 | CDK4 |
|  | Tumor necrosis factor | TNF |
|  | Transcription factor AP-1 | JUN |
|  | Interleukin-6 | IL6 |
|  | Cell division protein kinase 6 | CDK6 |
|  | Activator of 90 kDa heat shock protein ATPase homolog 1 | AHSA1 |
|  | Caspase-3 | CASP3 |
|  | Cellular tumor antigen p53 | TP53 |
|  | Mitogen-activated protein kinase 8 | MAPK8 |
|  | NF-kappa-B inhibitor alpha | NFKBIA |
|  | Ornithine decarboxylase | ODC1 |
|  | Xanthine dehydrogenase/oxidase | XDH |
|  | Bcl-2 homologous antagonist/killer | BAK1 |
|  | Bcl-2-binding component 3 | BBC3 |
|  | Superoxide dismutase [Cu-Zn] | SOD1 |
|  | Catalase | CAT |
|  | Protein kinase C alpha type | PRKCA |
|  | Telomerase protein component 1 | TEP1 |
|  | Hypoxia-inducible factor 1-alpha | HIF1A |
|  | Insulin-like growth factor 1 receptor | IGF1R |
|  | Signal transducer and activator of transcription 1-alpha/beta | STAT1 |
|  | Protein CBFA2T1 | RUNX1T1 |
|  | Cell division control protein 2 homolog | CDK1 |
|  | Endothelin-1 | EDN1 |
|  | 78 kDa glucose-regulated protein | HSPA5 |
|  | Forkhead box protein O1 | FOXO1 |
|  | Heparin-binding growth factor 2 | FGF2 |
|  | Peroxisome proliferator-activated receptor gamma | PPARG |
|  | Catenin beta-1 | CTNNB1 |
|  | Myc proto-oncogene protein | MYC |
|  | Tissue factor | F3 |
|  | Gap junction alpha-1 protein | GJA1 |
|  | Cytochrome P450 1A1 | CYP1A1 |
|  | Intercellular adhesion molecule 1 | ICAM1 |
|  | Interleukin-1 beta | IL1B |
|  | C-C motif chemokine 2 | CCL2 |
|  | E-selectin | SELE |
|  | Vascular cell adhesion protein 1 | VCAM1 |
|  | Protein kinase C delta type | PRKCD |
|  | Interleukin-8 | CXCL8 |
|  | Induced myeloid leukemia cell differentiation protein Mcl-1 | MCL1 |
|  | Breast cancer type 1 susceptibility protein | BRCA1 |
|  | X-ray repair cross-complementing protein 6 | XRCC6 |
|  | Superoxide dismutase [Mn], mitochondrial | SOD2 |
|  | Platelet endothelial cell adhesion molecule | PECAM1 |
|  | Baculoviral IAP repeat-containing protein 5 | BIRC5 |
|  | Dual oxidase 2 | DUOX2 |
|  | Nitric oxide synthase, endothelial | NOS3 |
|  | Tyrosine-protein phosphatase non-receptor type 1 | PTPN13 |
|  | G1/S-specific cyclin-D2 | CCND2 |
|  | Bcl-2-related protein A1 | BCL2A1 |
|  | Multidrug resistance protein 1 | ABCB1 |
|  | Hepatocyte growth factor | HGF |
|  | Transforming growth factor beta-1 | TGFB1 |
|  | Estrogen sulfotransferase | SULT1E1 |
|  | Maltase-glucoamylase, intestinal | MGAM |
|  | Cytochrome P450 1B1 | CYP1B1 |
|  | G2/mitotic-specific cyclin-B1 | CCNB1 |
|  | DNA damage-inducible transcript 3 protein | DDIT3 |
|  | Tissue-type plasminogen activator | PLAT |
|  | Phosphatidylinositol-3,4,5-trisphosphate 3-phosphatase and dual-specificity protein phosphatase PTEN | PTEN |
|  | Cell division protein kinase 7 | CDK7 |
|  | Interleukin-1 alpha | IL1A |
|  | Myeloperoxidase | MPO |
|  | Tumor necrosis factor receptor superfamily member 10B | TNFRSF10B |
|  | Cytochrome P450 19A1 | CYP19A1 |
|  | ATP-binding cassette sub-family G member 2 | ABCA2 |
|  | Nuclear factor erythroid 2-related factor 2 | NFE2L2 |
|  | Baculoviral IAP repeat-containing protein 4 | XIAP |
|  | Aryl hydrocarbon receptor | AHR |
|  | 5'-AMP-activated protein kinase subunit gamma-2 | PRKAG2 |
|  | Solute carrier family 2, facilitated glucose transporter member 4 | SLC2A4 |
|  | Peroxisome proliferator-activated receptor alpha | PPARA |
|  | C-reactive protein | CRP |
|  | Serum paraoxonase/arylesterase 1 | PON1 |
|  | T-lymphocyte activation antigen CD80 | CD80 |
|  | G1/S-specific cyclin-E1 | CCNE1 |
|  | G1/S-specific cyclin-E2 | CCNE2 |
|  | Basal cell adhesion molecule | BCAM |
|  | NAD-dependent deacetylase sirtuin-1 | SIRT1 |
|  | CREB/ATF bZIP transcription factor | CREBZF |
|  | High affinity nerve growth factor receptor | NTRK1 |
|  | Krueppel-like factor 10 | KLF10 |
|  | Apoptotic protease-activating factor 1 | APAF1 |
|  | Canalicular multispecific organic anion transporter 2 | ABCC3 |
|  | Adiponectin receptor protein 1 | ADIPOR1 |
|  | Adiponectin receptor protein 2 | ADIPOR2 |
|  | Tyrosine-protein kinase JAK1 | JAK1 |
|  | Type-1 angiotensin II receptor | AGTR1 |
|  | Insulin receptor substrate 1 | IRS1 |
|  | CASP8 and FADD-like apoptosis regulator | CFLAR |
|  | Lengsin | LGSN |
|  | Pygopus homolog 1 | PYGO1 |
|  | Interleukin-17B | IL17B |
|  | NAD-dependent deacetylase sirtuin-2 | SIRT2 |
|  | C5a anaphylatoxin chemotactic receptor | C5AR1 |
|  | Serine/threonine-protein kinase mTOR | MTOR |
|  | C-C chemokine receptor type 2 | CCR2 |
|  | Eukaryotic translation initiation factor 2 subunit 1 | EIF2S1 |
|  | Prostaglandin E synthase | PTGES |
|  | Phorbol-12-myristate-13-acetate-induced protein 1 | PMAIP1 |
|  | Bcl-2-like protein 11 | BCL2L11 |
|  | Tumor necrosis factor ligand superfamily member 10 | TNFSF10 |
|  | Tumor necrosis factor receptor superfamily member 10A | TNFRSF10A |
|  | GTP cyclohydrolase 1 | GCH1 |
|  | Baculoviral IAP repeat-containing protein 3 | BIRC3 |
|  | TNF receptor-associated factor 2 | TRAF2 |
|  | Cell division control protein 42 homolog | CDC42 |
|  | Basigin | BSG |
|  | Collagen alpha-1(II) chain | COL2A1 |
|  | Integrin beta-1 | ITGB1 |
|  | T-cell-specific surface glycoprotein CD28 | CD28 |
|  | Oxysterols receptor LXR-alpha | NR1H3 |
|  | Pappalysin-1 | PAPPA |
|  | SPARC | SPARC |
|  | Breast cancer type 2 susceptibility protein | BRCA2 |
|  | Alpha- and gamma-adaptin-binding protein p34 | AAGAB |
|  | Serine/threonine-protein kinase D1 | PRKD1 |
|  | Protransforming growth factor alpha | TGFA |
|  | CD320 antigen | CD320 |
|  | Transforming growth factor beta-2 | TGFB2 |
|  | Heat shock protein HSP 90 | HSP90AB1 |
|  | mRNA of PKA Catalytic Subunit C-alpha | PRKACA |
|  | 40S ribosomal protein S6 | RPS6 |
|  | Sterol regulatory element-binding protein 1 | SREBF1 |
|  | NAD(P)H dehydrogenase [quinone] 1 | NQO1 |
|  | Protein kinase C beta type | PRKCB |
|  | Probable E3 ubiquitin-protein ligase HERC5 | HERC5 |

The date of data search is March 2021
