# Supplementary material for: Resveratrol Ameliorates Lipopolysaccharide-Induced Sudden Sensorineural Hearing Loss in In Vitro Model through Multitarget Antiapoptotic Mechanism Based on Network Pharmacology and Molecular Docking
Source: Evid Based Complement Alternat Med. 2022 May 19;2022:6404588. doi: 10.1155/2022/6404588 (PMC9135530; doi:10.1155/2022/6404588)
Supplement: Supplementary Materials — Table S1. Targets of resveratrol from the TCMSP database (151 resveratrol target proteins were obtained and transformed into corresponding differential genes). Table S2. Targets of SSNHL from the DisGeNET database (2342 corresponding target genes were obtained). Table S3. RSV-SSNHL potential targets and Cytoscape analysis results (a total of 2416 nodes were obtained by PPI analysis, and the number of nodes was selected as greater than or equal to 70). [file 6404588.f1.zip › 6404588.f1/Table S2 SSNHL-SearchResults.docx]

**Table S2 Targets of SSNHL from the DisGeNET database**

| **Gene Symbol** | **Description** | **Category** | **Gifts** | **GC Id** |
| --- | --- | --- | --- | --- |
| GJB2 | Gap Junction Protein Beta 2 | Protein Coding | 47 | GC13M020187 |
| CDH23 | Cadherin Related 23 | Protein Coding | 41 | GC10P071396 |
| SLC26A4 | Solute Carrier Family 26 Member 4 | Protein Coding | 43 | GC07P107660 |
| MYO7A | Myosin VIIA | Protein Coding | 42 | GC11P077128 |
| GJB6 | Gap Junction Protein Beta 6 | Protein Coding | 44 | GC13M020221 |
| TECTA | Tectorin Alpha | Protein Coding | 39 | GC11P121101 |
| COCH | Cochlin | Protein Coding | 42 | GC14P030874 |
| WFS1 | Wolframin ER Transmembrane Glycoprotein | Protein Coding | 45 | GC04P006271 |
| TMC1 | Transmembrane Channel Like 1 | Protein Coding | 39 | GC09P072521 |
| USH2A | Usherin | Protein Coding | 38 | GC01M215622 |
| MYH9 | Myosin Heavy Chain 9 | Protein Coding | 49 | GC22M036281 |
| KCNQ1 | Potassium Voltage-Gated Channel Subfamily Q Member 1 | Protein Coding | 49 | GC11P002444 |
| GJB3 | Gap Junction Protein Beta 3 | Protein Coding | 44 | GC01P034781 |
| WHRN | Whirlin | Protein Coding | 32 | GC09M114403 |
| MYO6 | Myosin VI | Protein Coding | 45 | GC06P075749 |
| IL6 | Interleukin 6 | Protein Coding | 50 | GC07P022765 |
| TNF | Tumor Necrosis Factor | Protein Coding | 51 | GC06P047305 |
| KCNJ10 | Potassium Inwardly Rectifying Channel Subfamily J Member 10 | Protein Coding | 44 | GC01M159998 |
| ADGRV1 | Adhesion G Protein-Coupled Receptor V1 | Protein Coding | 35 | GC05P090529 |
| KCNE1 | Potassium Voltage-Gated Channel Subfamily E Regulatory Subunit 1 | Protein Coding | 44 | GC21M034446 |
| KCNQ4 | Potassium Voltage-Gated Channel Subfamily Q Member 4 | Protein Coding | 44 | GC01P040784 |
| SCN5A | Sodium Voltage-Gated Channel Alpha Subunit 5 | Protein Coding | 50 | GC03M038549 |
| MYH14 | Myosin Heavy Chain 14 | Protein Coding | 45 | GC19P050192 |
| EYA4 | EYA Transcriptional Coactivator And Phosphatase 4 | Protein Coding | 43 | GC06P133240 |
| GJA1 | Gap Junction Protein Alpha 1 | Protein Coding | 50 | GC06P121436 |
| IL1B | Interleukin 1 Beta | Protein Coding | 48 | GC02M112829 |
| MITF | Melanocyte Inducing Transcription Factor | Protein Coding | 47 | GC03P069788 |
| MT-RNR1 | Mitochondrially Encoded 12S RRNA | RNA Gene | 14 | GCMTP000642 |
| TMIE | Transmembrane Inner Ear | Protein Coding | 33 | GC03P046717 |
| ACTG1 | Actin Gamma 1 | Protein Coding | 50 | GC17M081509 |
| ABHD12 | Abhydrolase Domain Containing 12, Lysophospholipase | Protein Coding | 40 | GC20M025294 |
| POLG | DNA Polymerase Gamma, Catalytic Subunit | Protein Coding | 45 | GC15M089316 |
| MT-ND1 | Mitochondrially Encoded NADH:Ubiquinone Oxidoreductase Core Subunit 1 | Protein Coding | 32 | GCMTP003309 |
| ATP1A3 | ATPase Na+/K+ Transporting Subunit Alpha 3 | Protein Coding | 47 | GC19M041966 |
| MT-CO1 | Mitochondrially Encoded Cytochrome C Oxidase I | Protein Coding | 32 | GCMTP005906 |
| MT-TL1 | Mitochondrially Encoded TRNA-Leu (UUA/G) 1 | RNA Gene | 15 | GCMTP003232 |
| FGFR3 | Fibroblast Growth Factor Receptor 3 | Protein Coding | 55 | GC04P001795 |
| ALB | Albumin | Protein Coding | 50 | GC04P073397 |
| LMNA | Lamin A/C | Protein Coding | 47 | GC01P156082 |
| MT-CYB | Mitochondrially Encoded Cytochrome B | Protein Coding | 31 | GCMTP014749 |
| DIAPH1 | Diaphanous Related Formin 1 | Protein Coding | 47 | GC05M141477 |
| TBC1D24 | TBC1 Domain Family Member 24 | Protein Coding | 39 | GC16P002475 |
| SOX10 | SRY-Box Transcription Factor 10 | Protein Coding | 45 | GC22M046233 |
| BDNF | Brain Derived Neurotrophic Factor | Protein Coding | 47 | GC11M027654 |
| COL2A1 | Collagen Type II Alpha 1 Chain | Protein Coding | 48 | GC12M047972 |
| EYA1 | EYA Transcriptional Coactivator And Phosphatase 1 | Protein Coding | 45 | GC08M071210 |
| IARS2 | Isoleucyl-TRNA Synthetase 2, Mitochondrial | Protein Coding | 41 | GC01P220094 |
| TP53 | Tumor Protein P53 | Protein Coding | 54 | GC17M007661 |
| PSAP | Prosaposin | Protein Coding | 46 | GC10M071816 |
| MT-TS1 | Mitochondrially Encoded TRNA-Ser (UCN) 1 | RNA Gene | 14 | GCMTM007447 |
| MT-ND4 | Mitochondrially Encoded NADH:Ubiquinone Oxidoreductase Core Subunit 4 | Protein Coding | 31 | GCMTP010762 |
| MTHFR | Methylenetetrahydrofolate Reductase | Protein Coding | 47 | GC01M011785 |
| INS | Insulin | Protein Coding | 48 | GC11M002159 |
| KCNH2 | Potassium Voltage-Gated Channel Subfamily H Member 2 | Protein Coding | 49 | GC07M150944 |
| APOE | Apolipoprotein E | Protein Coding | 50 | GC19P044906 |
| RPGR | Retinitis Pigmentosa GTPase Regulator | Protein Coding | 40 | GC0XM038269 |
| F2 | Coagulation Factor II, Thrombin | Protein Coding | 48 | GC11P046720 |
| RET | Ret Proto-Oncogene | Protein Coding | 53 | GC10P043081 |
| PEX6 | Peroxisomal Biogenesis Factor 6 | Protein Coding | 43 | GC06M042963 |
| IGF1 | Insulin Like Growth Factor 1 | Protein Coding | 50 | GC12M102395 |
| TIMM8A | Translocase Of Inner Mitochondrial Membrane 8A | Protein Coding | 43 | GC0XM101345 |
| MT-ATP6 | Mitochondrially Encoded ATP Synthase Membrane Subunit 6 | Protein Coding | 31 | GCMTP008531 |
| TWNK | Twinkle MtDNA Helicase | Protein Coding | 33 | GC10P100993 |
| CTNNB1 | Catenin Beta 1 | Protein Coding | 53 | GC03P041236 |
| FGFR1 | Fibroblast Growth Factor Receptor 1 | Protein Coding | 55 | GC08M038400 |
| EDNRB | Endothelin Receptor Type B | Protein Coding | 49 | GC13M077895 |
| MT-TK | Mitochondrially Encoded TRNA-Lys (AAA/G) | RNA Gene | 14 | GCMTP008297 |
| ERCC6 | ERCC Excision Repair 6, Chromatin Remodeling Factor | Protein Coding | 45 | GC10M049454 |
| MT-ND6 | Mitochondrially Encoded NADH:Ubiquinone Oxidoreductase Core Subunit 6 | Protein Coding | 32 | GCMTM014151 |
| OPA1 | OPA1 Mitochondrial Dynamin Like GTPase | Protein Coding | 44 | GC03P193594 |
| HGF | Hepatocyte Growth Factor | Protein Coding | 52 | GC07M081699 |
| CAT | Catalase | Protein Coding | 50 | GC11P034460 |
| CACNA1C | Calcium Voltage-Gated Channel Subunit Alpha1 C | Protein Coding | 48 | GC12P001970 |
| IL10 | Interleukin 10 | Protein Coding | 47 | GC01M206767 |
| NLRP3 | NLR Family Pyrin Domain Containing 3 | Protein Coding | 47 | GC01P247415 |
| TYR | Tyrosinase | Protein Coding | 47 | GC11P089177 |
| ABCA4 | ATP Binding Cassette Subfamily A Member 4 | Protein Coding | 44 | GC01M093992 |
| MPZ | Myelin Protein Zero | Protein Coding | 44 | GC01M161304 |
| MECP2 | Methyl-CpG Binding Protein 2 | Protein Coding | 45 | GC0XM154021 |
| AKT1 | AKT Serine/Threonine Kinase 1 | Protein Coding | 54 | GC14M104769 |
| DNMT1 | DNA Methyltransferase 1 | Protein Coding | 50 | GC19M010133 |
| RHO | Rhodopsin | Protein Coding | 47 | GC03P130619 |
| RPE65 | Retinoid Isomerohydrolase RPE65 | Protein Coding | 44 | GC01M068428 |
| CRYAA | Crystallin Alpha A | Protein Coding | 44 | GC21P043169 |
| ACTB | Actin Beta | Protein Coding | 49 | GC07M005527 |
| EPRS1 | Glutamyl-Prolyl-TRNA Synthetase 1 | Protein Coding | 36 | GC01M219969 |
| TRIOBP | TRIO And F-Actin Binding Protein | Protein Coding | 40 | GC22P037696 |
| PEX1 | Peroxisomal Biogenesis Factor 1 | Protein Coding | 44 | GC07M092487 |
| RYR2 | Ryanodine Receptor 2 | Protein Coding | 47 | GC01P237042 |
| SPATA5 | Spermatogenesis Associated 5 | Protein Coding | 40 | GC04P122923 |
| DMD | Dystrophin | Protein Coding | 46 | GC0XM031047 |
| KCNE5 | Potassium Voltage-Gated Channel Subfamily E Regulatory Subunit 5 | Protein Coding | 32 | GC0XM109623 |
| MAPT | Microtubule Associated Protein Tau | Protein Coding | 50 | GC17P045894 |
| SNCA | Synuclein Alpha | Protein Coding | 50 | GC04M089724 |
| GJB1 | Gap Junction Protein Beta 1 | Protein Coding | 47 | GC0XP071215 |
| F5 | Coagulation Factor V | Protein Coding | 45 | GC01M169511 |
| PRPH2 | Peripherin 2 | Protein Coding | 39 | GC06M043145 |
| BCS1L | BCS1 Homolog, Ubiquinol-Cytochrome C Reductase Complex Chaperone | Protein Coding | 43 | GC02P218658 |
| MFN2 | Mitofusin 2 | Protein Coding | 48 | GC01P011980 |
| DLX5 | Distal-Less Homeobox 5 | Protein Coding | 44 | GC07M097020 |
| COL1A1 | Collagen Type I Alpha 1 Chain | Protein Coding | 50 | GC17M050183 |
| HLA-DRB1 | Major Histocompatibility Complex, Class II, DR Beta 1 | Protein Coding | 46 | GC06M032578 |
| SOD1 | Superoxide Dismutase 1 | Protein Coding | 51 | GC21P031659 |
| KCNJ2 | Potassium Inwardly Rectifying Channel Subfamily J Member 2 | Protein Coding | 48 | GC17P070168 |
| FBN1 | Fibrillin 1 | Protein Coding | 45 | GC15M048408 |
| CRX | Cone-Rod Homeobox | Protein Coding | 43 | GC19P047819 |
| NGF | Nerve Growth Factor | Protein Coding | 50 | GC01M115285 |
| COL1A2 | Collagen Type I Alpha 2 Chain | Protein Coding | 47 | GC07P094394 |
| PSEN1 | Presenilin 1 | Protein Coding | 52 | GC14P073136 |
| PTPN11 | Protein Tyrosine Phosphatase Non-Receptor Type 11 | Protein Coding | 53 | GC12P112418 |
| ARSA | Arylsulfatase A | Protein Coding | 47 | GC22M050622 |
| PDE6B | Phosphodiesterase 6B | Protein Coding | 45 | GC04P000587 |
| ELN | Elastin | Protein Coding | 44 | GC07P074027 |
| EDN3 | Endothelin 3 | Protein Coding | 47 | GC20P059300 |
| SLC25A4 | Solute Carrier Family 25 Member 4 | Protein Coding | 48 | GC04P185143 |
| TLR4 | Toll Like Receptor 4 | Protein Coding | 51 | GC09P117704 |
| MYH7 | Myosin Heavy Chain 7 | Protein Coding | 47 | GC14M023412 |
| MARVELD2 | MARVEL Domain Containing 2 | Protein Coding | 39 | GC05P069415 |
| TULP1 | TUB Like Protein 1 | Protein Coding | 42 | GC06M042216 |
| ARSG | Arylsulfatase G | Protein Coding | 43 | GC17P068259 |
| COL9A2 | Collagen Type IX Alpha 2 Chain | Protein Coding | 42 | GC01M040300 |
| CHAT | Choline O-Acetyltransferase | Protein Coding | 47 | GC10P049609 |
| KCNE2 | Potassium Voltage-Gated Channel Subfamily E Regulatory Subunit 2 | Protein Coding | 41 | GC21P034364 |
| SDHD | Succinate Dehydrogenase Complex Subunit D | Protein Coding | 44 | GC11P112087 |
| BSND | Barttin CLCNK Type Accessory Subunit Beta | Protein Coding | 40 | GC01P054998 |
| PEX26 | Peroxisomal Biogenesis Factor 26 | Protein Coding | 41 | GC22P018079 |
| ANK2 | Ankyrin 2 | Protein Coding | 41 | GC04P112706 |
| C9orf72 | C9orf72-SMCR8 Complex Subunit | Protein Coding | 41 | GC09M027539 |
| CAV3 | Caveolin 3 | Protein Coding | 43 | GC03P008733 |
| CRB1 | Crumbs Cell Polarity Complex Component 1 | Protein Coding | 43 | GC01P197170 |
| LEP | Leptin | Protein Coding | 47 | GC07P128241 |
| TYMP | Thymidine Phosphorylase | Protein Coding | 46 | GC22M050525 |
| FGFR2 | Fibroblast Growth Factor Receptor 2 | Protein Coding | 54 | GC10M121478 |
| GFAP | Glial Fibrillary Acidic Protein | Protein Coding | 47 | GC17M044905 |
| SOX2 | SRY-Box Transcription Factor 2 | Protein Coding | 47 | GC03P181711 |
| KITLG | KIT Ligand | Protein Coding | 44 | GC12M088492 |
| MT-ND5 | Mitochondrially Encoded NADH:Ubiquinone Oxidoreductase Core Subunit 5 | Protein Coding | 31 | GCMTP012339 |
| MYH6 | Myosin Heavy Chain 6 | Protein Coding | 45 | GC14M023380 |
| MT-CO3 | Mitochondrially Encoded Cytochrome C Oxidase III | Protein Coding | 30 | GCMTP009209 |
| GDNF | Glial Cell Derived Neurotrophic Factor | Protein Coding | 47 | GC05M037812 |
| PEX10 | Peroxisomal Biogenesis Factor 10 | Protein Coding | 41 | GC01M002403 |
| DGUOK | Deoxyguanosine Kinase | Protein Coding | 44 | GC02P073926 |
| GUCY2D | Guanylate Cyclase 2D, Retinal | Protein Coding | 43 | GC17P008002 |
| FLNA | Filamin A | Protein Coding | 49 | GC0XM154348 |
| TGFB1 | Transforming Growth Factor Beta 1 | Protein Coding | 52 | GC19M041301 |
| SLC52A3 | Solute Carrier Family 52 Member 3 | Protein Coding | 39 | GC20M000741 |
| FOXI1 | Forkhead Box I1 | Protein Coding | 37 | GC05P170105 |
| SLC19A2 | Solute Carrier Family 19 Member 2 | Protein Coding | 46 | GC01M169463 |
| PMP22 | Peripheral Myelin Protein 22 | Protein Coding | 40 | GC17M015229 |
| BRAF | B-Raf Proto-Oncogene, Serine/Threonine Kinase | Protein Coding | 54 | GC07M140719 |
| GLA | Galactosidase Alpha | Protein Coding | 48 | GC0XM101393 |
| P2RX2 | Purinergic Receptor P2X 2 | Protein Coding | 43 | GC12P132618 |
| CEP290 | Centrosomal Protein 290 | Protein Coding | 40 | GC12M088049 |
| SCN1A | Sodium Voltage-Gated Channel Alpha Subunit 1 | Protein Coding | 47 | GC02M165989 |
| RDH12 | Retinol Dehydrogenase 12 | Protein Coding | 45 | GC14P067701 |
| ACE | Angiotensin I Converting Enzyme | Protein Coding | 49 | GC17P063477 |
| TNNT2 | Troponin T2, Cardiac Type | Protein Coding | 48 | GC01M201359 |
| PEX2 | Peroxisomal Biogenesis Factor 2 | Protein Coding | 43 | GC08M076980 |
| PEX12 | Peroxisomal Biogenesis Factor 12 | Protein Coding | 39 | GC17M035574 |
| ALMS1 | ALMS1 Centrosome And Basal Body Associated Protein | Protein Coding | 41 | GC02P073385 |
| CCL2 | C-C Motif Chemokine Ligand 2 | Protein Coding | 48 | GC17P034255 |
| NKX2-5 | NK2 Homeobox 5 | Protein Coding | 44 | GC05M173232 |
| MT-CO2 | Mitochondrially Encoded Cytochrome C Oxidase II | Protein Coding | 32 | GCMTP007587 |
| TTN | Titin | Protein Coding | 47 | GC02M178525 |
| MT-TS2 | Mitochondrially Encoded TRNA-Ser (AGU/C) 2 | RNA Gene | 13 | GCMTP012215 |
| VCP | Valosin Containing Protein | Protein Coding | 48 | GC09M035056 |
| ITGB3 | Integrin Subunit Beta 3 | Protein Coding | 49 | GC17P047254 |
| SNTA1 | Syntrophin Alpha 1 | Protein Coding | 43 | GC20M033407 |
| RRM2B | Ribonucleotide Reductase Regulatory TP53 Inducible Subunit M2B | Protein Coding | 49 | GC08M102204 |
| IL1RN | Interleukin 1 Receptor Antagonist | Protein Coding | 48 | GC02P115307 |
| SERPINC1 | Serpin Family C Member 1 | Protein Coding | 48 | GC01M174153 |
| MIR96 | MicroRNA 96 | RNA Gene | 20 | GC07M129774 |
| IFNG | Interferon Gamma | Protein Coding | 48 | GC12M068064 |
| SCN4B | Sodium Voltage-Gated Channel Beta Subunit 4 | Protein Coding | 43 | GC11M118134 |
| CLCNKB | Chloride Voltage-Gated Channel Kb | Protein Coding | 44 | GC01P016043 |
| BTD | Biotinidase | Protein Coding | 44 | GC03P015621 |
| ROM1 | Retinal Outer Segment Membrane Protein 1 | Protein Coding | 43 | GC11P062611 |
| MYBPC3 | Myosin Binding Protein C3 | Protein Coding | 46 | GC11M061124 |
| SDHA | Succinate Dehydrogenase Complex Flavoprotein Subunit A | Protein Coding | 46 | GC05P000208 |
| CCR6 | C-C Motif Chemokine Receptor 6 | Protein Coding | 44 | GC06P167111 |
| PRODH | Proline Dehydrogenase 1 | Protein Coding | 45 | GC22M018912 |
| EGF | Epidermal Growth Factor | Protein Coding | 51 | GC04P109912 |
| KRAS | KRAS Proto-Oncogene, GTPase | Protein Coding | 51 | GC12M025204 |
| PEX13 | Peroxisomal Biogenesis Factor 13 | Protein Coding | 40 | GC02P061017 |
| PTPN22 | Protein Tyrosine Phosphatase Non-Receptor Type 22 | Protein Coding | 46 | GC01M113813 |
| PRPF31 | Pre-MRNA Processing Factor 31 | Protein Coding | 42 | GC19P055363 |
| SLC6A4 | Solute Carrier Family 6 Member 4 | Protein Coding | 47 | GC17M030194 |
| SERAC1 | Serine Active Site Containing 1 | Protein Coding | 36 | GC06M158109 |
| COL9A3 | Collagen Type IX Alpha 3 Chain | Protein Coding | 42 | GC20P062816 |
| TTR | Transthyretin | Protein Coding | 49 | GC18P031557 |
| DNM1L | Dynamin 1 Like | Protein Coding | 47 | GC12P032679 |
| HSD17B4 | Hydroxysteroid 17-Beta Dehydrogenase 4 | Protein Coding | 46 | GC05P119452 |
| GJB4 | Gap Junction Protein Beta 4 | Protein Coding | 40 | GC01P034759 |
| TNFRSF1A | TNF Receptor Superfamily Member 1A | Protein Coding | 49 | GC12M006328 |
| GRHL2 | Grainyhead Like Transcription Factor 2 | Protein Coding | 40 | GC08P101492 |
| SALL1 | Spalt Like Transcription Factor 1 | Protein Coding | 44 | GC16M051135 |
| IDUA | Alpha-L-Iduronidase | Protein Coding | 42 | GC04P000986 |
| PEX5 | Peroxisomal Biogenesis Factor 5 | Protein Coding | 42 | GC12P008247 |
| COMT | Catechol-O-Methyltransferase | Protein Coding | 51 | GC22P019941 |
| BMP4 | Bone Morphogenetic Protein 4 | Protein Coding | 49 | GC14M053949 |
| TNFSF11 | TNF Superfamily Member 11 | Protein Coding | 47 | GC13P042562 |
| PAX6 | Paired Box 6 | Protein Coding | 47 | GC11M031784 |
| PTEN | Phosphatase And Tensin Homolog | Protein Coding | 52 | GC10P087863 |
| GLI3 | GLI Family Zinc Finger 3 | Protein Coding | 48 | GC07M041960 |
| AIFM1 | Apoptosis Inducing Factor Mitochondria Associated 1 | Protein Coding | 49 | GC0XM130129 |
| DARS2 | Aspartyl-TRNA Synthetase 2, Mitochondrial | Protein Coding | 41 | GC01P173824 |
| SURF1 | SURF1 Cytochrome C Oxidase Assembly Factor | Protein Coding | 43 | GC09M133351 |
| DNAJC19 | DnaJ Heat Shock Protein Family (Hsp40) Member C19 | Protein Coding | 41 | GC03M180983 |
| FAS | Fas Cell Surface Death Receptor | Protein Coding | 50 | GC10P088969 |
| TARDBP | TAR DNA Binding Protein | Protein Coding | 45 | GC01P011013 |
| NOTCH3 | Notch Receptor 3 | Protein Coding | 49 | GC19M015159 |
| CFH | Complement Factor H | Protein Coding | 45 | GC01P196621 |
| MT-TF | Mitochondrially Encoded TRNA-Phe (UUU/C) | RNA Gene | 14 | GCMTP000580 |
| SQSTM1 | Sequestosome 1 | Protein Coding | 48 | GC05P179806 |
| HRAS | HRas Proto-Oncogene, GTPase | Protein Coding | 52 | GC11M000635 |
| NEFL | Neurofilament Light | Protein Coding | 46 | GC08M024950 |
| TLR2 | Toll Like Receptor 2 | Protein Coding | 51 | GC04P153684 |
| MET | MET Proto-Oncogene, Receptor Tyrosine Kinase | Protein Coding | 54 | GC07P116672 |
| OTOG | Otogelin | Protein Coding | 33 | GC11P017530 |
| ATRX | ATRX Chromatin Remodeler | Protein Coding | 45 | GC0XM077504 |
| FOS | Fos Proto-Oncogene, AP-1 Transcription Factor Subunit | Protein Coding | 50 | GC14P075278 |
| APP | Amyloid Beta Precursor Protein | Protein Coding | 51 | GC21M025880 |
| CASQ2 | Calsequestrin 2 | Protein Coding | 44 | GC01M115700 |
| CLDN14 | Claudin 14 | Protein Coding | 44 | GC21M036460 |
| SNAP25 | Synaptosome Associated Protein 25 | Protein Coding | 49 | GC20P010218 |
| SEMA3A | Semaphorin 3A | Protein Coding | 45 | GC07M083955 |
| SDHB | Succinate Dehydrogenase Complex Iron Sulfur Subunit B | Protein Coding | 47 | GC01M017020 |
| SLC52A2 | Solute Carrier Family 52 Member 2 | Protein Coding | 37 | GC08P144354 |
| NF1 | Neurofibromin 1 | Protein Coding | 48 | GC17P031094 |
| EP300 | E1A Binding Protein P300 | Protein Coding | 50 | GC22P041091 |
| ZMPSTE24 | Zinc Metallopeptidase STE24 | Protein Coding | 43 | GC01P040258 |
| ACOX1 | Acyl-CoA Oxidase 1 | Protein Coding | 45 | GC17M075941 |
| NARS2 | Asparaginyl-TRNA Synthetase 2, Mitochondrial | Protein Coding | 43 | GC11M078435 |
| BTK | Bruton Tyrosine Kinase | Protein Coding | 53 | GC0XM101349 |
| HADHA | Hydroxyacyl-CoA Dehydrogenase Trifunctional Multienzyme Complex Subunit Alpha | Protein Coding | 45 | GC02M026190 |
| FUS | FUS RNA Binding Protein | Protein Coding | 44 | GC16P031180 |
| CACNA1A | Calcium Voltage-Gated Channel Subunit Alpha1 A | Protein Coding | 47 | GC19M013206 |
| PPARG | Peroxisome Proliferator Activated Receptor Gamma | Protein Coding | 52 | GC03P012287 |
| MAPK1 | Mitogen-Activated Protein Kinase 1 | Protein Coding | 51 | GC22M021754 |
| PRKN | Parkin RBR E3 Ubiquitin Protein Ligase | Protein Coding | 40 | GC06M161348 |
| CTSA | Cathepsin A | Protein Coding | 45 | GC20P045890 |
| SCO2 | Synthesis Of Cytochrome C Oxidase 2 | Protein Coding | 45 | GC22M050523 |
| TRMU | TRNA 5-Methylaminomethyl-2-Thiouridylate Methyltransferase | Protein Coding | 39 | GC22P046330 |
| DCAF17 | DDB1 And CUL4 Associated Factor 17 | Protein Coding | 36 | GC02P171434 |
| MT-ND2 | Mitochondrially Encoded NADH:Ubiquinone Oxidoreductase Core Subunit 2 | Protein Coding | 32 | GCMTP004472 |
| SLC6A3 | Solute Carrier Family 6 Member 3 | Protein Coding | 49 | GC05M001392 |
| SYNE4 | Spectrin Repeat Containing Nuclear Envelope Family Member 4 | Protein Coding | 32 | GC19M036003 |
| OTX2 | Orthodenticle Homeobox 2 | Protein Coding | 45 | GC14M056799 |
| ESR1 | Estrogen Receptor 1 | Protein Coding | 53 | GC06P151656 |
| FKBP14 | FKBP Prolyl Isomerase 14 | Protein Coding | 40 | GC07M030010 |
| RLBP1 | Retinaldehyde Binding Protein 1 | Protein Coding | 44 | GC15M089209 |
| CTLA4 | Cytotoxic T-Lymphocyte Associated Protein 4 | Protein Coding | 45 | GC02P203867 |
| PIK3R1 | Phosphoinositide-3-Kinase Regulatory Subunit 1 | Protein Coding | 51 | GC05P068215 |
| COQ6 | Coenzyme Q6, Monooxygenase | Protein Coding | 42 | GC14P073949 |
| SMAD4 | SMAD Family Member 4 | Protein Coding | 50 | GC18P051028 |
| MPV17 | Mitochondrial Inner Membrane Protein MPV17 | Protein Coding | 41 | GC02M027309 |
| TNNI3 | Troponin I3, Cardiac Type | Protein Coding | 48 | GC19M055151 |
| CISD2 | CDGSH Iron Sulfur Domain 2 | Protein Coding | 41 | GC04P102868 |
| SRC | SRC Proto-Oncogene, Non-Receptor Tyrosine Kinase | Protein Coding | 51 | GC20P037344 |
| EGFR | Epidermal Growth Factor Receptor | Protein Coding | 54 | GC07P055019 |
| HSPD1 | Heat Shock Protein Family D (Hsp60) Member 1 | Protein Coding | 47 | GC02M197486 |
| POLG2 | DNA Polymerase Gamma 2, Accessory Subunit | Protein Coding | 41 | GC17M064477 |
| TSPEAR | Thrombospondin Type Laminin G Domain And EAR Repeats | Protein Coding | 32 | GC21M044497 |
| NOTCH1 | Notch Receptor 1 | Protein Coding | 51 | GC09M136602 |
| SNAI2 | Snail Family Transcriptional Repressor 2 | Protein Coding | 44 | GC08M048854 |
| DSG2 | Desmoglein 2 | Protein Coding | 45 | GC18P031498 |
| OPA3 | Outer Mitochondrial Membrane Lipid Metabolism Regulator OPA3 | Protein Coding | 39 | GC19M045527 |
| GATA3 | GATA Binding Protein 3 | Protein Coding | 49 | GC10P008045 |
| GARS1 | Glycyl-TRNA Synthetase 1 | Protein Coding | 36 | GC07P030595 |
| ACADM | Acyl-CoA Dehydrogenase Medium Chain | Protein Coding | 47 | GC01P075724 |
| PAX2 | Paired Box 2 | Protein Coding | 46 | GC10P100735 |
| KIT | KIT Proto-Oncogene, Receptor Tyrosine Kinase | Protein Coding | 53 | GC04P054657 |
| APTX | Aprataxin | Protein Coding | 44 | GC09M032886 |
| REST | RE1 Silencing Transcription Factor | Protein Coding | 44 | GC04P056907 |
| EDN1 | Endothelin 1 | Protein Coding | 47 | GC06P012290 |
| GNRH1 | Gonadotropin Releasing Hormone 1 | Protein Coding | 41 | GC08M025419 |
| SYP | Synaptophysin | Protein Coding | 43 | GC0XM049187 |
| CCND1 | Cyclin D1 | Protein Coding | 52 | GC11P069641 |
| GNAS | GNAS Complex Locus | Protein Coding | 50 | GC20P058839 |
| ACSL4 | Acyl-CoA Synthetase Long Chain Family Member 4 | Protein Coding | 44 | GC0XM109624 |
| FXN | Frataxin | Protein Coding | 46 | GC09P069035 |
| PEX7 | Peroxisomal Biogenesis Factor 7 | Protein Coding | 43 | GC06P136822 |
| FMR1 | FMRP Translational Regulator 1 | Protein Coding | 44 | GC0XP147913 |
| CDKN2A | Cyclin Dependent Kinase Inhibitor 2A | Protein Coding | 51 | GC09M021967 |
| STAT3 | Signal Transducer And Activator Of Transcription 3 | Protein Coding | 52 | GC17M042313 |
| FGF10 | Fibroblast Growth Factor 10 | Protein Coding | 47 | GC05M044340 |
| JAG1 | Jagged Canonical Notch Ligand 1 | Protein Coding | 50 | GC20M010637 |
| COL4A1 | Collagen Type IV Alpha 1 Chain | Protein Coding | 47 | GC13M110148 |
| SLC4A1 | Solute Carrier Family 4 Member 1 (Diego Blood Group) | Protein Coding | 47 | GC17M044282 |
| ERCC2 | ERCC Excision Repair 2, TFIIH Core Complex Helicase Subunit | Protein Coding | 47 | GC19M045349 |
| BRCA1 | BRCA1 DNA Repair Associated | Protein Coding | 50 | GC17M043044 |
| MTOR | Mechanistic Target Of Rapamycin Kinase | Protein Coding | 54 | GC01M011106 |
| PEX16 | Peroxisomal Biogenesis Factor 16 | Protein Coding | 38 | GC11M061098 |
| MT-TW | Mitochondrially Encoded TRNA-Trp (UGA/G) | RNA Gene | 12 | GCMTP005514 |
| MT-TV | Mitochondrially Encoded TRNA-Val (GUN) | RNA Gene | 14 | GCMTP001605 |
| PEX3 | Peroxisomal Biogenesis Factor 3 | Protein Coding | 42 | GC06P143450 |
| COQ2 | Coenzyme Q2, Polyprenyltransferase | Protein Coding | 41 | GC04M083261 |
| ANXA5 | Annexin A5 | Protein Coding | 46 | GC04M121667 |
| IGF2R | Insulin Like Growth Factor 2 Receptor | Protein Coding | 45 | GC06P159969 |
| MT-TI | Mitochondrially Encoded TRNA-Ile (AUU/C) | RNA Gene | 13 | GCMTP004265 |
| PKD1 | Polycystin 1, Transient Receptor Potential Channel Interacting | Protein Coding | 45 | GC16M002348 |
| SLC12A3 | Solute Carrier Family 12 Member 3 | Protein Coding | 47 | GC16P056865 |
| PEX19 | Peroxisomal Biogenesis Factor 19 | Protein Coding | 43 | GC01M160276 |
| MT-TH | Mitochondrially Encoded TRNA-His (CAU/C) | RNA Gene | 13 | GCMTP012140 |
| DES | Desmin | Protein Coding | 48 | GC02P219418 |
| CALM1 | Calmodulin 1 | Protein Coding | 45 | GC14P090396 |
| HTR1A | 5-Hydroxytryptamine Receptor 1A | Protein Coding | 47 | GC05M063960 |
| NTRK2 | Neurotrophic Receptor Tyrosine Kinase 2 | Protein Coding | 53 | GC09P084668 |
| CRP | C-Reactive Protein | Protein Coding | 46 | GC01M159716 |
| BBS2 | Bardet-Biedl Syndrome 2 | Protein Coding | 41 | GC16M056467 |
| RAF1 | Raf-1 Proto-Oncogene, Serine/Threonine Kinase | Protein Coding | 54 | GC03M012583 |
| KCNJ11 | Potassium Inwardly Rectifying Channel Subfamily J Member 11 | Protein Coding | 47 | GC11M017364 |
| GUCA1A | Guanylate Cyclase Activator 1A | Protein Coding | 42 | GC06P047516 |
| HLA-DQB1 | Major Histocompatibility Complex, Class II, DQ Beta 1 | Protein Coding | 44 | GC06M032804 |
| CDHR1 | Cadherin Related Family Member 1 | Protein Coding | 39 | GC10P084194 |
| CRH | Corticotropin Releasing Hormone | Protein Coding | 44 | GC08M066176 |
| KCNE3 | Potassium Voltage-Gated Channel Subfamily E Regulatory Subunit 3 | Protein Coding | 44 | GC11M074454 |
| TLR3 | Toll Like Receptor 3 | Protein Coding | 52 | GC04P186059 |
| PHYH | Phytanoyl-CoA 2-Hydroxylase | Protein Coding | 45 | GC10M013277 |
| PEX14 | Peroxisomal Biogenesis Factor 14 | Protein Coding | 43 | GC01P010472 |
| LARS2 | Leucyl-TRNA Synthetase 2, Mitochondrial | Protein Coding | 45 | GC03P045405 |
| SPG7 | SPG7 Matrix AAA Peptidase Subunit, Paraplegin | Protein Coding | 43 | GC16P089492 |
| GP1BA | Glycoprotein Ib Platelet Subunit Alpha | Protein Coding | 45 | GC17P004932 |
| BEST1 | Bestrophin 1 | Protein Coding | 43 | GC11P061949 |
| MT-ND3 | Mitochondrially Encoded NADH:Ubiquinone Oxidoreductase Core Subunit 3 | Protein Coding | 31 | GCMTP010061 |
| TGFB3 | Transforming Growth Factor Beta 3 | Protein Coding | 47 | GC14M075958 |
| HCRT | Hypocretin Neuropeptide Precursor | Protein Coding | 40 | GC17M042185 |
| PROM1 | Prominin 1 | Protein Coding | 44 | GC04M015965 |
| PITX2 | Paired Like Homeodomain 2 | Protein Coding | 47 | GC04M110617 |
| TCOF1 | Treacle Ribosome Biogenesis Factor 1 | Protein Coding | 42 | GC05P150358 |
| COX10 | Cytochrome C Oxidase Assembly Factor Heme A:Farnesyltransferase COX10 | Protein Coding | 43 | GC17P014069 |
| KCNQ3 | Potassium Voltage-Gated Channel Subfamily Q Member 3 | Protein Coding | 45 | GC08M132120 |
| BMP2 | Bone Morphogenetic Protein 2 | Protein Coding | 47 | GC20P006696 |
| SOX9 | SRY-Box Transcription Factor 9 | Protein Coding | 47 | GC17P072121 |
| DCTN2 | Dynactin Subunit 2 | Protein Coding | 41 | GC12M057530 |
| PEX11B | Peroxisomal Biogenesis Factor 11 Beta | Protein Coding | 40 | GC01M145911 |
| GBA | Glucosylceramidase Beta | Protein Coding | 47 | GC01M155234 |
| CD2AP | CD2 Associated Protein | Protein Coding | 43 | GC06P047777 |
| VHL | Von Hippel-Lindau Tumor Suppressor | Protein Coding | 47 | GC03P010211 |
| VEGFA | Vascular Endothelial Growth Factor A | Protein Coding | 48 | GC06P043770 |
| SOX3 | SRY-Box Transcription Factor 3 | Protein Coding | 44 | GC0XM140502 |
| KIF1B | Kinesin Family Member 1B | Protein Coding | 44 | GC01P010210 |
| AVP | Arginine Vasopressin | Protein Coding | 45 | GC20M003082 |
| ECE1 | Endothelin Converting Enzyme 1 | Protein Coding | 47 | GC01M021217 |
| AIPL1 | Aryl Hydrocarbon Receptor Interacting Protein Like 1 | Protein Coding | 42 | GC17M006393 |
| CNGB1 | Cyclic Nucleotide Gated Channel Subunit Beta 1 | Protein Coding | 43 | GC16M057884 |
| GDAP1 | Ganglioside Induced Differentiation Associated Protein 1 | Protein Coding | 42 | GC08P074315 |
| PIK3CA | Phosphatidylinositol-4,5-Bisphosphate 3-Kinase Catalytic Subunit Alpha | Protein Coding | 52 | GC03P179148 |
| GNAT2 | G Protein Subunit Alpha Transducin 2 | Protein Coding | 45 | GC01M109603 |
| FBN2 | Fibrillin 2 | Protein Coding | 41 | GC05M128257 |
| RUNX2 | RUNX Family Transcription Factor 2 | Protein Coding | 47 | GC06P047549 |
| ABCC8 | ATP Binding Cassette Subfamily C Member 8 | Protein Coding | 45 | GC11M017392 |
| TMEM126A | Transmembrane Protein 126A | Protein Coding | 39 | GC11P085647 |
| KCNA1 | Potassium Voltage-Gated Channel Subfamily A Member 1 | Protein Coding | 44 | GC12P008111 |
| NOS3 | Nitric Oxide Synthase 3 | Protein Coding | 51 | GC07P150990 |
| PSEN2 | Presenilin 2 | Protein Coding | 49 | GC01P226870 |
| ENPP1 | Ectonucleotide Pyrophosphatase/Phosphodiesterase 1 | Protein Coding | 47 | GC06P131808 |
| DNMT3A | DNA Methyltransferase 3 Alpha | Protein Coding | 51 | GC02M025228 |
| SALL4 | Spalt Like Transcription Factor 4 | Protein Coding | 43 | GC20M051784 |
| NDUFA1 | NADH:Ubiquinone Oxidoreductase Subunit A1 | Protein Coding | 44 | GC0XP119871 |
| BRCA2 | BRCA2 DNA Repair Associated | Protein Coding | 49 | GC13P032315 |
| SAG | S-Antigen Visual Arrestin | Protein Coding | 44 | GC02P233311 |
| NDUFS8 | NADH:Ubiquinone Oxidoreductase Core Subunit S8 | Protein Coding | 45 | GC11P068030 |
| GNRHR | Gonadotropin Releasing Hormone Receptor | Protein Coding | 47 | GC04M067737 |
| PLP1 | Proteolipid Protein 1 | Protein Coding | 43 | GC0XP103773 |
| LRRK2 | Leucine Rich Repeat Kinase 2 | Protein Coding | 49 | GC12P040196 |
| TRPV4 | Transient Receptor Potential Cation Channel Subfamily V Member 4 | Protein Coding | 49 | GC12M109783 |
| SUCLG1 | Succinate-CoA Ligase GDP/ADP-Forming Subunit Alpha | Protein Coding | 45 | GC02M084423 |
| ETFDH | Electron Transfer Flavoprotein Dehydrogenase | Protein Coding | 44 | GC04P158672 |
| SSBP1 | Single Stranded DNA Binding Protein 1 | Protein Coding | 38 | GC07P145133 |
| TBK1 | TANK Binding Kinase 1 | Protein Coding | 49 | GC12P064451 |
| LMNB1 | Lamin B1 | Protein Coding | 47 | GC05P126776 |
| DMXL2 | Dmx Like 2 | Protein Coding | 36 | GC15M051447 |
| IQSEC2 | IQ Motif And Sec7 Domain ArfGEF 2 | Protein Coding | 37 | GC0XM053225 |
| MAP2K1 | Mitogen-Activated Protein Kinase Kinase 1 | Protein Coding | 54 | GC15P066386 |
| RAB7A | RAB7A, Member RAS Oncogene Family | Protein Coding | 48 | GC03P128749 |
| NDUFAF2 | NADH:Ubiquinone Oxidoreductase Complex Assembly Factor 2 | Protein Coding | 40 | GC05P060945 |
| VDR | Vitamin D Receptor | Protein Coding | 51 | GC12M047841 |
| ASCL1 | Achaete-Scute Family BHLH Transcription Factor 1 | Protein Coding | 44 | GC12P102957 |
| SMAD3 | SMAD Family Member 3 | Protein Coding | 49 | GC15P067063 |
| TBL1X | Transducin Beta Like 1 X-Linked | Protein Coding | 36 | GC0XP009463 |
| MMP2 | Matrix Metallopeptidase 2 | Protein Coding | 53 | GC16P055390 |
| MARS2 | Methionyl-TRNA Synthetase 2, Mitochondrial | Protein Coding | 42 | GC02P197705 |
| PLA2G6 | Phospholipase A2 Group VI | Protein Coding | 47 | GC22M046185 |
| GABRD | Gamma-Aminobutyric Acid Type A Receptor Subunit Delta | Protein Coding | 45 | GC01P002019 |
| H2AC18 | H2A Clustered Histone 18 | Protein Coding | 26 | GC01M149961 |
| MIR21 | MicroRNA 21 | RNA Gene | 24 | GC17P059841 |
| LONP1 | Lon Peptidase 1, Mitochondrial | Protein Coding | 43 | GC19M005691 |
| HTT | Huntingtin | Protein Coding | 43 | GC04P003041 |
| SPP1 | Secreted Phosphoprotein 1 | Protein Coding | 46 | GC04P087975 |
| CLN6 | CLN6 Transmembrane ER Protein | Protein Coding | 37 | GC15M068206 |
| TFB1M | Transcription Factor B1, Mitochondrial | Protein Coding | 42 | GC06M155247 |
| GALC | Galactosylceramidase | Protein Coding | 43 | GC14M087837 |
| NPHP1 | Nephrocystin 1 | Protein Coding | 43 | GC02M110122 |
| AFG3L2 | AFG3 Like Matrix AAA Peptidase Subunit 2 | Protein Coding | 44 | GC18M012328 |
| NDUFV2 | NADH:Ubiquinone Oxidoreductase Core Subunit V2 | Protein Coding | 44 | GC18P009092 |
| ATP1A2 | ATPase Na+/K+ Transporting Subunit Alpha 2 | Protein Coding | 47 | GC01P160115 |
| HLA-A | Major Histocompatibility Complex, Class I, A | Protein Coding | 46 | GC06P047265 |
| NDUFS4 | NADH:Ubiquinone Oxidoreductase Subunit S4 | Protein Coding | 44 | GC05P053560 |
| SACS | Sacsin Molecular Chaperone | Protein Coding | 36 | GC13M023328 |
| CDH1 | Cadherin 1 | Protein Coding | 50 | GC16P068737 |
| FOXG1 | Forkhead Box G1 | Protein Coding | 43 | GC14P028766 |
| BSCL2 | BSCL2 Lipid Droplet Biogenesis Associated, Seipin | Protein Coding | 42 | GC11M063438 |
| SGSH | N-Sulfoglucosamine Sulfohydrolase | Protein Coding | 44 | GC17M080206 |
| PCNT | Pericentrin | Protein Coding | 41 | GC21P046324 |
| GUSB | Glucuronidase Beta | Protein Coding | 47 | GC07M065960 |
| CACNA1S | Calcium Voltage-Gated Channel Subunit Alpha1 S | Protein Coding | 48 | GC01M201008 |
| FIG4 | FIG4 Phosphoinositide 5-Phosphatase | Protein Coding | 43 | GC06P109691 |
| MBL2 | Mannose Binding Lectin 2 | Protein Coding | 47 | GC10M052760 |
| MT-TQ | Mitochondrially Encoded TRNA-Gln (CAA/G) | RNA Gene | 12 | GCMTM004331 |
| TGFB2 | Transforming Growth Factor Beta 2 | Protein Coding | 50 | GC01P218345 |
| AP1S1 | Adaptor Related Protein Complex 1 Subunit Sigma 1 | Protein Coding | 39 | GC07P101154 |
| PTCH1 | Patched 1 | Protein Coding | 50 | GC09M095442 |
| HLA-B | Major Histocompatibility Complex, Class I, B | Protein Coding | 45 | GC06M031315 |
| TNFRSF1B | TNF Receptor Superfamily Member 1B | Protein Coding | 47 | GC01P012167 |
| NDUFS2 | NADH:Ubiquinone Oxidoreductase Core Subunit S2 | Protein Coding | 44 | GC01P161197 |
| MEN1 | Menin 1 | Protein Coding | 46 | GC11M064803 |
| ACTN2 | Actinin Alpha 2 | Protein Coding | 47 | GC01P236686 |
| ERBB2 | Erb-B2 Receptor Tyrosine Kinase 2 | Protein Coding | 54 | GC17P039687 |
| CASP8 | Caspase 8 | Protein Coding | 52 | GC02P201233 |
| IGF2 | Insulin Like Growth Factor 2 | Protein Coding | 48 | GC11M002130 |
| KCNJ13 | Potassium Inwardly Rectifying Channel Subfamily J Member 13 | Protein Coding | 44 | GC02M232765 |
| ARX | Aristaless Related Homeobox | Protein Coding | 42 | GC0XM025021 |
| CHCHD10 | Coiled-Coil-Helix-Coiled-Coil-Helix Domain Containing 10 | Protein Coding | 39 | GC22M023765 |
| CASK | Calcium/Calmodulin Dependent Serine Protein Kinase | Protein Coding | 48 | GC0XM041514 |
| GRN | Granulin Precursor | Protein Coding | 47 | GC17P044345 |
| SDHC | Succinate Dehydrogenase Complex Subunit C | Protein Coding | 44 | GC01P161314 |
| FARS2 | Phenylalanyl-TRNA Synthetase 2, Mitochondrial | Protein Coding | 44 | GC06P005261 |
| NAGLU | N-Acetyl-Alpha-Glucosaminidase | Protein Coding | 43 | GC17P042535 |
| TAC3 | Tachykinin Precursor 3 | Protein Coding | 45 | GC12M057009 |
| SOST | Sclerostin | Protein Coding | 44 | GC17M043753 |
| MEOX1 | Mesenchyme Homeobox 1 | Protein Coding | 42 | GC17M043640 |
| PRKG1 | Protein Kinase CGMP-Dependent 1 | Protein Coding | 51 | GC10P050991 |
| MT-ATP8 | Mitochondrially Encoded ATP Synthase Membrane Subunit 8 | Protein Coding | 28 | GCMTP008368 |
| SLC2A1 | Solute Carrier Family 2 Member 1 | Protein Coding | 52 | GC01M042925 |
| TH | Tyrosine Hydroxylase | Protein Coding | 51 | GC11M002163 |
| TGM1 | Transglutaminase 1 | Protein Coding | 45 | GC14M024249 |
| TSC2 | TSC Complex Subunit 2 | Protein Coding | 50 | GC16P002436 |
| NDUFV1 | NADH:Ubiquinone Oxidoreductase Core Subunit V1 | Protein Coding | 45 | GC11P067632 |
| TMEM126B | Transmembrane Protein 126B | Protein Coding | 39 | GC11P085628 |
| POMGNT1 | Protein O-Linked Mannose N-Acetylglucosaminyltransferase 1 (Beta 1,2-) | Protein Coding | 45 | GC01M046188 |
| DSPP | Dentin Sialophosphoprotein | Protein Coding | 37 | GC04P087608 |
| PRNP | Prion Protein | Protein Coding | 47 | GC20P004686 |
| DCTN1 | Dynactin Subunit 1 | Protein Coding | 46 | GC02M074361 |
| HBB | Hemoglobin Subunit Beta | Protein Coding | 45 | GC11M005352 |
| PRTN3 | Proteinase 3 | Protein Coding | 44 | GC19P000840 |
| NRAS | NRAS Proto-Oncogene, GTPase | Protein Coding | 50 | GC01M114704 |
| NSD1 | Nuclear Receptor Binding SET Domain Protein 1 | Protein Coding | 43 | GC05P177134 |
| COX15 | Cytochrome C Oxidase Assembly Homolog COX15 | Protein Coding | 42 | GC10M099696 |
| GJC2 | Gap Junction Protein Gamma 2 | Protein Coding | 41 | GC01P228175 |
| FOXE3 | Forkhead Box E3 | Protein Coding | 35 | GC01P047416 |
| DCX | Doublecortin | Protein Coding | 45 | GC0XM111293 |
| PDGFRB | Platelet Derived Growth Factor Receptor Beta | Protein Coding | 55 | GC05M150113 |
| COQ8A | Coenzyme Q8A | Protein Coding | 36 | GC01P226899 |
| CST3 | Cystatin C | Protein Coding | 44 | GC20M023608 |
| SERPINF1 | Serpin Family F Member 1 | Protein Coding | 44 | GC17P001761 |
| CASR | Calcium Sensing Receptor | Protein Coding | 50 | GC03P122183 |
| RBP3 | Retinol Binding Protein 3 | Protein Coding | 40 | GC10P047348 |
| GABRA1 | Gamma-Aminobutyric Acid Type A Receptor Subunit Alpha1 | Protein Coding | 46 | GC05P161847 |
| GRIN2B | Glutamate Ionotropic Receptor NMDA Type Subunit 2B | Protein Coding | 50 | GC12M013437 |
| EDNRA | Endothelin Receptor Type A | Protein Coding | 49 | GC04P147480 |
| CLN3 | CLN3 Lysosomal/Endosomal Transmembrane Protein, Battenin | Protein Coding | 43 | GC16M028466 |
| GLB1 | Galactosidase Beta 1 | Protein Coding | 48 | GC03M033013 |
| GRIN1 | Glutamate Ionotropic Receptor NMDA Type Subunit 1 | Protein Coding | 49 | GC09P137138 |
| TIMP3 | TIMP Metallopeptidase Inhibitor 3 | Protein Coding | 45 | GC22P032800 |
| MPO | Myeloperoxidase | Protein Coding | 50 | GC17M058269 |
| LORICRIN | Loricrin Cornified Envelope Precursor Protein | Protein Coding | 27 | GC01P153262 |
| ENG | Endoglin | Protein Coding | 46 | GC09M127815 |
| HSD17B10 | Hydroxysteroid 17-Beta Dehydrogenase 10 | Protein Coding | 45 | GC0XM053431 |
| B2M | Beta-2-Microglobulin | Protein Coding | 48 | GC15P044711 |
| DNAH8 | Dynein Axonemal Heavy Chain 8 | Protein Coding | 37 | GC06P047481 |
| KCNV2 | Potassium Voltage-Gated Channel Modifier Subfamily V Member 2 | Protein Coding | 40 | GC09P002717 |
| EGR2 | Early Growth Response 2 | Protein Coding | 43 | GC10M062811 |
| C4A | Complement C4A (Rodgers Blood Group) | Protein Coding | 42 | GC06P047332 |
| TAZ | Tafazzin | Protein Coding | 44 | GC0XP154411 |
| CD40LG | CD40 Ligand | Protein Coding | 47 | GC0XP136649 |
| ACTC1 | Actin Alpha Cardiac Muscle 1 | Protein Coding | 42 | GC15M034788 |
| HUWE1 | HECT, UBA And WWE Domain Containing E3 Ubiquitin Protein Ligase 1 | Protein Coding | 44 | GC0XM053532 |
| CACNA1D | Calcium Voltage-Gated Channel Subunit Alpha1 D | Protein Coding | 47 | GC03P053328 |
| EMD | Emerin | Protein Coding | 45 | GC0XP154379 |
| DCN | Decorin | Protein Coding | 47 | GC12M091140 |
| ABCD1 | ATP Binding Cassette Subfamily D Member 1 | Protein Coding | 45 | GC0XP153724 |
| RDH5 | Retinol Dehydrogenase 5 | Protein Coding | 46 | GC12P055720 |
| CACNA1F | Calcium Voltage-Gated Channel Subunit Alpha1 F | Protein Coding | 45 | GC0XM049205 |
| NR2E3 | Nuclear Receptor Subfamily 2 Group E Member 3 | Protein Coding | 41 | GC15P071792 |
| AGK | Acylglycerol Kinase | Protein Coding | 41 | GC07P141551 |
| KCNQ1OT1 | KCNQ1 Opposite Strand/Antisense Transcript 1 | RNA Gene | 25 | GC11M002661 |
| NAGA | Alpha-N-Acetylgalactosaminidase | Protein Coding | 43 | GC22M042058 |
| TSC1 | TSC Complex Subunit 1 | Protein Coding | 48 | GC09M132891 |
| DLX6 | Distal-Less Homeobox 6 | Protein Coding | 39 | GC07P097005 |
| RTEL1 | Regulator Of Telomere Elongation Helicase 1 | Protein Coding | 40 | GC20P063658 |
| LAMA4 | Laminin Subunit Alpha 4 | Protein Coding | 44 | GC06M112107 |
| CDKL5 | Cyclin Dependent Kinase Like 5 | Protein Coding | 43 | GC0XP018425 |
| NRL | Neural Retina Leucine Zipper | Protein Coding | 41 | GC14M024078 |
| GATA4 | GATA Binding Protein 4 | Protein Coding | 48 | GC08P011676 |
| CXCL8 | C-X-C Motif Chemokine Ligand 8 | Protein Coding | 41 | GC04P073740 |
| SERPINA3 | Serpin Family A Member 3 | Protein Coding | 43 | GC14P094612 |
| NTRK1 | Neurotrophic Receptor Tyrosine Kinase 1 | Protein Coding | 48 | GC01P156786 |
| SETBP1 | SET Binding Protein 1 | Protein Coding | 40 | GC18P044680 |
| ERCC3 | ERCC Excision Repair 3, TFIIH Core Complex Helicase Subunit | Protein Coding | 47 | GC02M127257 |
| SMC3 | Structural Maintenance Of Chromosomes 3 | Protein Coding | 45 | GC10P110567 |
| ACAD9 | Acyl-CoA Dehydrogenase Family Member 9 | Protein Coding | 43 | GC03P130611 |
| SMARCA4 | SWI/SNF Related, Matrix Associated, Actin Dependent Regulator Of Chromatin, Subfamily A, Member 4 | Protein Coding | 50 | GC19P010932 |
| CASP3 | Caspase 3 | Protein Coding | 50 | GC04M184627 |
| ATP13A2 | ATPase Cation Transporting 13A2 | Protein Coding | 42 | GC01M016985 |
| PON1 | Paraoxonase 1 | Protein Coding | 45 | GC07M095297 |
| CNTNAP2 | Contactin Associated Protein 2 | Protein Coding | 44 | GC07P146116 |
| SPG11 | SPG11 Vesicle Trafficking Associated, Spatacsin | Protein Coding | 39 | GC15M044562 |
| TREX1 | Three Prime Repair Exonuclease 1 | Protein Coding | 42 | GC03P048466 |
| RYR1 | Ryanodine Receptor 1 | Protein Coding | 47 | GC19P038528 |
| ALG13 | ALG13 UDP-N-Acetylglucosaminyltransferase Subunit | Protein Coding | 38 | GC0XP111665 |
| HLA-DPB1 | Major Histocompatibility Complex, Class II, DP Beta 1 | Protein Coding | 43 | GC06P047346 |
| GJA5 | Gap Junction Protein Alpha 5 | Protein Coding | 45 | GC01M147756 |
| PRRT2 | Proline Rich Transmembrane Protein 2 | Protein Coding | 39 | GC16P029811 |
| TCF4 | Transcription Factor 4 | Protein Coding | 46 | GC18M055222 |
| TECTB | Tectorin Beta | Protein Coding | 31 | GC10P112283 |
| RBM20 | RNA Binding Motif Protein 20 | Protein Coding | 35 | GC10P110644 |
| CD19 | CD19 Molecule | Protein Coding | 49 | GC16P029083 |
| MGME1 | Mitochondrial Genome Maintenance Exonuclease 1 | Protein Coding | 35 | GC20P017952 |
| PHOX2B | Paired Like Homeobox 2B | Protein Coding | 43 | GC04M041746 |
| MT-TE | Mitochondrially Encoded TRNA-Glu (GAA/G) | RNA Gene | 12 | GCMTM014676 |
| LMNB2 | Lamin B2 | Protein Coding | 43 | GC19M002439 |
| DNAJC3 | DnaJ Heat Shock Protein Family (Hsp40) Member C3 | Protein Coding | 40 | GC13P095677 |
| CRYAB | Crystallin Alpha B | Protein Coding | 45 | GC11M111908 |
| ANKH | ANKH Inorganic Pyrophosphate Transport Regulator | Protein Coding | 40 | GC05M014706 |
| PLN | Phospholamban | Protein Coding | 44 | GC06P118548 |
| NFKB1 | Nuclear Factor Kappa B Subunit 1 | Protein Coding | 52 | GC04P102501 |
| PINK1 | PTEN Induced Kinase 1 | Protein Coding | 47 | GC01P020634 |
| CALR | Calreticulin | Protein Coding | 51 | GC19P012938 |
| SCN2B | Sodium Voltage-Gated Channel Beta Subunit 2 | Protein Coding | 43 | GC11M118163 |
| MYOD1 | Myogenic Differentiation 1 | Protein Coding | 46 | GC11P017741 |
| MAG | Myelin Associated Glycoprotein | Protein Coding | 45 | GC19P035292 |
| CYCS | Cytochrome C, Somatic | Protein Coding | 48 | GC07M025118 |
| SCN10A | Sodium Voltage-Gated Channel Alpha Subunit 10 | Protein Coding | 45 | GC03M038713 |
| SOD2 | Superoxide Dismutase 2 | Protein Coding | 51 | GC06M159669 |
| PMM2 | Phosphomannomutase 2 | Protein Coding | 47 | GC16P008788 |
| TMEM132E | Transmembrane Protein 132E | Protein Coding | 35 | GC17P034580 |
| PIK3C2A | Phosphatidylinositol-4-Phosphate 3-Kinase Catalytic Subunit Type 2 Alpha | Protein Coding | 47 | GC11M017191 |
| IMPG2 | Interphotoreceptor Matrix Proteoglycan 2 | Protein Coding | 38 | GC03M101222 |
| CTSD | Cathepsin D | Protein Coding | 52 | GC11M001752 |
| GMPPB | GDP-Mannose Pyrophosphorylase B | Protein Coding | 43 | GC03M049716 |
| NRXN1 | Neurexin 1 | Protein Coding | 47 | GC02M049918 |
| ASAH1 | N-Acylsphingosine Amidohydrolase 1 | Protein Coding | 46 | GC08M018055 |
| STAT1 | Signal Transducer And Activator Of Transcription 1 | Protein Coding | 53 | GC02M190908 |
| SNAP29 | Synaptosome Associated Protein 29 | Protein Coding | 42 | GC22P020859 |
| HLA-DQA1 | Major Histocompatibility Complex, Class II, DQ Alpha 1 | Protein Coding | 42 | GC06P047340 |
| ELANE | Elastase, Neutrophil Expressed | Protein Coding | 46 | GC19P000854 |
| CARS2 | Cysteinyl-TRNA Synthetase 2, Mitochondrial | Protein Coding | 41 | GC13M110641 |
| CNGB3 | Cyclic Nucleotide Gated Channel Subunit Beta 3 | Protein Coding | 40 | GC08M086553 |
| MYOC | Myocilin | Protein Coding | 42 | GC01M171604 |
| LDLR | Low Density Lipoprotein Receptor | Protein Coding | 49 | GC19P011061 |
| LRP4 | LDL Receptor Related Protein 4 | Protein Coding | 43 | GC11M061111 |
| HADH | Hydroxyacyl-CoA Dehydrogenase | Protein Coding | 47 | GC04P107989 |
| CX3CR1 | C-X3-C Motif Chemokine Receptor 1 | Protein Coding | 44 | GC03M039279 |
| ZFYVE26 | Zinc Finger FYVE-Type Containing 26 | Protein Coding | 39 | GC14M067727 |
| KCND3 | Potassium Voltage-Gated Channel Subfamily D Member 3 | Protein Coding | 46 | GC01M111770 |
| NDUFB3 | NADH:Ubiquinone Oxidoreductase Subunit B3 | Protein Coding | 42 | GC02P201071 |
| OPTN | Optineurin | Protein Coding | 44 | GC10P013099 |
| HADHB | Hydroxyacyl-CoA Dehydrogenase Trifunctional Multienzyme Complex Subunit Beta | Protein Coding | 47 | GC02P026243 |
| IQCB1 | IQ Motif Containing B1 | Protein Coding | 41 | GC03M121769 |
| TTPA | Alpha Tocopherol Transfer Protein | Protein Coding | 41 | GC08M063048 |
| SMPD1 | Sphingomyelin Phosphodiesterase 1 | Protein Coding | 47 | GC11P006390 |
| SYNE1 | Spectrin Repeat Containing Nuclear Envelope Protein 1 | Protein Coding | 41 | GC06M152121 |
| DCHS1 | Dachsous Cadherin-Related 1 | Protein Coding | 39 | GC11M006621 |
| ELOVL4 | ELOVL Fatty Acid Elongase 4 | Protein Coding | 45 | GC06M079914 |
| ABCA1 | ATP Binding Cassette Subfamily A Member 1 | Protein Coding | 48 | GC09M104781 |
| NDUFS7 | NADH:Ubiquinone Oxidoreductase Core Subunit S7 | Protein Coding | 46 | GC19P001490 |
| CSF1R | Colony Stimulating Factor 1 Receptor | Protein Coding | 52 | GC05M150053 |
| MEFV | MEFV Innate Immuity Regulator, Pyrin | Protein Coding | 43 | GC16M003281 |
| MYPN | Myopalladin | Protein Coding | 41 | GC10P068106 |
| PRKAG2 | Protein Kinase AMP-Activated Non-Catalytic Subunit Gamma 2 | Protein Coding | 50 | GC07M151556 |
| PNPLA2 | Patatin Like Phospholipase Domain Containing 2 | Protein Coding | 44 | GC11P000896 |
| SLC1A3 | Solute Carrier Family 1 Member 3 | Protein Coding | 50 | GC05P036606 |
| NDUFB11 | NADH:Ubiquinone Oxidoreductase Subunit B11 | Protein Coding | 39 | GC0XM047142 |
| TCAP | Titin-Cap | Protein Coding | 42 | GC17P039664 |
| C1S | Complement C1s | Protein Coding | 45 | GC12P008238 |
| HCN4 | Hyperpolarization Activated Cyclic Nucleotide Gated Potassium Channel 4 | Protein Coding | 47 | GC15M073319 |
| LOX | Lysyl Oxidase | Protein Coding | 44 | GC05M122063 |
| DEPDC5 | DEP Domain Containing 5, GATOR1 Subcomplex Subunit | Protein Coding | 40 | GC22P031753 |
| TBX1 | T-Box Transcription Factor 1 | Protein Coding | 42 | GC22P019747 |
| ABCC9 | ATP Binding Cassette Subfamily C Member 9 | Protein Coding | 44 | GC12M021797 |
| NOS1 | Nitric Oxide Synthase 1 | Protein Coding | 49 | GC12M117208 |
| LDB3 | LIM Domain Binding 3 | Protein Coding | 41 | GC10P086666 |
| CERS3 | Ceramide Synthase 3 | Protein Coding | 39 | GC15M103923 |
| DNM1 | Dynamin 1 | Protein Coding | 50 | GC09P128191 |
| KCNJ5 | Potassium Inwardly Rectifying Channel Subfamily J Member 5 | Protein Coding | 47 | GC11P128891 |
| AQP4 | Aquaporin 4 | Protein Coding | 45 | GC18M026852 |
| SLC25A13 | Solute Carrier Family 25 Member 13 | Protein Coding | 46 | GC07M096120 |
| RNU4ATAC | RNA, U4atac Small Nuclear (U12-Dependent Splicing) | RNA Gene | 20 | GC02P121532 |
| SOS1 | SOS Ras/Rac Guanine Nucleotide Exchange Factor 1 | Protein Coding | 47 | GC02M038981 |
| SEMA3D | Semaphorin 3D | Protein Coding | 39 | GC07M084995 |
| DNMT3B | DNA Methyltransferase 3 Beta | Protein Coding | 50 | GC20P032762 |
| PDSS1 | Decaprenyl Diphosphate Synthase Subunit 1 | Protein Coding | 42 | GC10P026697 |
| SCN4A | Sodium Voltage-Gated Channel Alpha Subunit 4 | Protein Coding | 44 | GC17M063938 |
| FTSJ1 | FtsJ RNA 2'-O-Methyltransferase 1 | Protein Coding | 41 | GC0XP048476 |
| NF2 | Neurofibromin 2 | Protein Coding | 48 | GC22P029603 |
| IL1A | Interleukin 1 Alpha | Protein Coding | 44 | GC02M112773 |
| NEU1 | Neuraminidase 1 | Protein Coding | 44 | GC06M031857 |
| YME1L1 | YME1 Like 1 ATPase | Protein Coding | 42 | GC10M027110 |
| CDC42 | Cell Division Cycle 42 | Protein Coding | 51 | GC01P022057 |
| RAC1 | Rac Family Small GTPase 1 | Protein Coding | 49 | GC07P006380 |
| KLHL7 | Kelch Like Family Member 7 | Protein Coding | 41 | GC07P023105 |
| GATAD1 | GATA Zinc Finger Domain Containing 1 | Protein Coding | 37 | GC07P092447 |
| SNX14 | Sorting Nexin 14 | Protein Coding | 39 | GC06M085505 |
| COX6B1 | Cytochrome C Oxidase Subunit 6B1 | Protein Coding | 43 | GC19P038224 |
| PARK7 | Parkinsonism Associated Deglycase | Protein Coding | 45 | GC01P007983 |
| AKAP9 | A-Kinase Anchoring Protein 9 | Protein Coding | 43 | GC07P091940 |
| NPC1 | NPC Intracellular Cholesterol Transporter 1 | Protein Coding | 47 | GC18M023506 |
| HMOX1 | Heme Oxygenase 1 | Protein Coding | 52 | GC22P035380 |
| PANK2 | Pantothenate Kinase 2 | Protein Coding | 43 | GC20P003887 |
| CNGA3 | Cyclic Nucleotide Gated Channel Subunit Alpha 3 | Protein Coding | 43 | GC02P098329 |
| MT-ND4L | Mitochondrially Encoded NADH:Ubiquinone Oxidoreductase Core Subunit 4L | Protein Coding | 28 | GCMTP010472 |
| AKT2 | AKT Serine/Threonine Kinase 2 | Protein Coding | 54 | GC19M040230 |
| RS1 | Retinoschisin 1 | Protein Coding | 40 | GC0XM018567 |
| FKTN | Fukutin | Protein Coding | 39 | GC09P105558 |
| PDGFRA | Platelet Derived Growth Factor Receptor Alpha | Protein Coding | 55 | GC04P054229 |
| NEXN | Nexilin F-Actin Binding Protein | Protein Coding | 39 | GC01P077898 |
| PTH | Parathyroid Hormone | Protein Coding | 47 | GC11M013492 |
| ATF6 | Activating Transcription Factor 6 | Protein Coding | 47 | GC01P161766 |
| HLA-C | Major Histocompatibility Complex, Class I, C | Protein Coding | 44 | GC06M031272 |
| DRD3 | Dopamine Receptor D3 | Protein Coding | 44 | GC03M114128 |
| KCNAB2 | Potassium Voltage-Gated Channel Subfamily A Regulatory Beta Subunit 2 | Protein Coding | 43 | GC01P006020 |
| AGRN | Agrin | Protein Coding | 44 | GC01P001020 |
| GAN | Gigaxonin | Protein Coding | 39 | GC16P081319 |
| FLNC | Filamin C | Protein Coding | 44 | GC07P128830 |
| MIR132 | MicroRNA 132 | RNA Gene | 21 | GC17M002049 |
| XYLT2 | Xylosyltransferase 2 | Protein Coding | 45 | GC17P050347 |
| LAMP2 | Lysosomal Associated Membrane Protein 2 | Protein Coding | 44 | GC0XM120426 |
| SUFU | SUFU Negative Regulator Of Hedgehog Signaling | Protein Coding | 43 | GC10P102503 |
| DKK1 | Dickkopf WNT Signaling Pathway Inhibitor 1 | Protein Coding | 45 | GC10P052314 |
| DLG3 | Discs Large MAGUK Scaffold Protein 3 | Protein Coding | 41 | GC0XP070444 |
| GSN | Gelsolin | Protein Coding | 48 | GC09P121201 |
| PRDM16 | PR/SET Domain 16 | Protein Coding | 45 | GC01P003068 |
| MAP2K2 | Mitogen-Activated Protein Kinase Kinase 2 | Protein Coding | 53 | GC19M004090 |
| APC | APC Regulator Of WNT Signaling Pathway | Protein Coding | 48 | GC05P112707 |
| SLC25A1 | Solute Carrier Family 25 Member 1 | Protein Coding | 46 | GC22M019176 |
| RB1 | RB Transcriptional Corepressor 1 | Protein Coding | 49 | GC13P048303 |
| MICOS13 | Mitochondrial Contact Site And Cristae Organizing System Subunit 13 | Protein Coding | 27 | GC19M005688 |
| FASLG | Fas Ligand | Protein Coding | 47 | GC01P172628 |
| CFTR | CF Transmembrane Conductance Regulator | Protein Coding | 51 | GC07P117287 |
| RNASET2 | Ribonuclease T2 | Protein Coding | 44 | GC06M166929 |
| CYP1B1 | Cytochrome P450 Family 1 Subfamily B Member 1 | Protein Coding | 48 | GC02M038034 |
| MIR142 | MicroRNA 142 | RNA Gene | 20 | GC17M058331 |
| CD36 | CD36 Molecule | Protein Coding | 48 | GC07P080369 |
| TANGO2 | Transport And Golgi Organization 2 Homolog | Protein Coding | 35 | GC22P020017 |
| PPARGC1A | PPARG Coactivator 1 Alpha | Protein Coding | 46 | GC04M023755 |
| MLH1 | MutL Homolog 1 | Protein Coding | 48 | GC03P036993 |
| OFD1 | OFD1 Centriole And Centriolar Satellite Protein | Protein Coding | 41 | GC0XP013734 |
| FAM136A | Family With Sequence Similarity 136 Member A | Protein Coding | 37 | GC02M070296 |
| ETFA | Electron Transfer Flavoprotein Subunit Alpha | Protein Coding | 45 | GC15M076215 |
| KCTD7 | Potassium Channel Tetramerization Domain Containing 7 | Protein Coding | 38 | GC07P066628 |
| VSX2 | Visual System Homeobox 2 | Protein Coding | 40 | GC14P074239 |
| CFAP410 | Cilia And Flagella Associated Protein 410 | Protein Coding | 29 | GC21M044330 |
| WRN | WRN RecQ Like Helicase | Protein Coding | 45 | GC08P031033 |
| AGTR2 | Angiotensin II Receptor Type 2 | Protein Coding | 43 | GC0XP116170 |
| APOH | Apolipoprotein H | Protein Coding | 44 | GC17M066212 |
| PRKAR1A | Protein Kinase CAMP-Dependent Type I Regulatory Subunit Alpha | Protein Coding | 51 | GC17P068414 |
| MIR17 | MicroRNA 17 | RNA Gene | 21 | GC13P091350 |
| ADA | Adenosine Deaminase | Protein Coding | 51 | GC20M044620 |
| JAK2 | Janus Kinase 2 | Protein Coding | 54 | GC09P004985 |
| DNM2 | Dynamin 2 | Protein Coding | 49 | GC19P010718 |
| IL4 | Interleukin 4 | Protein Coding | 46 | GC05P132673 |
| EPG5 | Ectopic P-Granules Autophagy Protein 5 Homolog | Protein Coding | 36 | GC18M045800 |
| ESRRB | Estrogen Related Receptor Beta | Protein Coding | 50 | GC14P076310 |
| SLC5A7 | Solute Carrier Family 5 Member 7 | Protein Coding | 44 | GC02P107969 |
| COL8A2 | Collagen Type VIII Alpha 2 Chain | Protein Coding | 41 | GC01M036095 |
| LAMB2 | Laminin Subunit Beta 2 | Protein Coding | 44 | GC03M049121 |
| ITGA2 | Integrin Subunit Alpha 2 | Protein Coding | 45 | GC05P052989 |
| KDSR | 3-Ketodihydrosphingosine Reductase | Protein Coding | 42 | GC18M063327 |
| BAZ1B | Bromodomain Adjacent To Zinc Finger Domain 1B | Protein Coding | 40 | GC07M073440 |
| YAP1 | Yes1 Associated Transcriptional Regulator | Protein Coding | 47 | GC11P102110 |
| HSPA4 | Heat Shock Protein Family A (Hsp70) Member 4 | Protein Coding | 41 | GC05P133051 |
| NOTCH2 | Notch Receptor 2 | Protein Coding | 50 | GC01M119911 |
| KIF5A | Kinesin Family Member 5A | Protein Coding | 44 | GC12P057549 |
| VPS13B | Vacuolar Protein Sorting 13 Homolog B | Protein Coding | 39 | GC08P099011 |
| FKBP10 | FKBP Prolyl Isomerase 10 | Protein Coding | 40 | GC17P041812 |
| GP6 | Glycoprotein VI Platelet | Protein Coding | 44 | GC19M055013 |
| CHRNB2 | Cholinergic Receptor Nicotinic Beta 2 Subunit | Protein Coding | 44 | GC01P154568 |
| LIMK1 | LIM Domain Kinase 1 | Protein Coding | 49 | GC07P074082 |
| CFI | Complement Factor I | Protein Coding | 46 | GC04M109740 |
| SLC6A2 | Solute Carrier Family 6 Member 2 | Protein Coding | 48 | GC16P055656 |
| UCHL1 | Ubiquitin C-Terminal Hydrolase L1 | Protein Coding | 51 | GC04P041256 |
| LMX1B | LIM Homeobox Transcription Factor 1 Beta | Protein Coding | 45 | GC09P126614 |
| MIR140 | MicroRNA 140 | RNA Gene | 22 | GC16P069934 |
| NDE1 | NudE Neurodevelopment Protein 1 | Protein Coding | 43 | GC16P015661 |
| MRAP | Melanocortin 2 Receptor Accessory Protein | Protein Coding | 37 | GC21P032291 |
| SYNE2 | Spectrin Repeat Containing Nuclear Envelope Protein 2 | Protein Coding | 39 | GC14P063761 |
| ASS1 | Argininosuccinate Synthase 1 | Protein Coding | 48 | GC09P130444 |
| KCNT1 | Potassium Sodium-Activated Channel Subfamily T Member 1 | Protein Coding | 43 | GC09P135702 |
| NDUFB8 | NADH:Ubiquinone Oxidoreductase Subunit B8 | Protein Coding | 43 | GC10M100523 |
| PCDH19 | Protocadherin 19 | Protein Coding | 40 | GC0XM100291 |
| FOXL2 | Forkhead Box L2 | Protein Coding | 40 | GC03M138944 |
| NR2F1 | Nuclear Receptor Subfamily 2 Group F Member 1 | Protein Coding | 45 | GC05P093583 |
| HBA2 | Hemoglobin Subunit Alpha 2 | Protein Coding | 42 | GC16P001373 |
| ABHD5 | Abhydrolase Domain Containing 5, Lysophosphatidic Acid Acyltransferase | Protein Coding | 45 | GC03P043707 |
| DTNA | Dystrobrevin Alpha | Protein Coding | 42 | GC18P034493 |
| MRPL44 | Mitochondrial Ribosomal Protein L44 | Protein Coding | 39 | GC02P223957 |
| SETX | Senataxin | Protein Coding | 41 | GC09M132261 |
| SMN2 | Survival Of Motor Neuron 2, Centromeric | Protein Coding | 42 | GC05P070049 |
| MTR | 5-Methyltetrahydrofolate-Homocysteine Methyltransferase | Protein Coding | 46 | GC01P236795 |
| ATXN7 | Ataxin 7 | Protein Coding | 40 | GC03P063864 |
| CLCN7 | Chloride Voltage-Gated Channel 7 | Protein Coding | 45 | GC16M001444 |
| ATN1 | Atrophin 1 | Protein Coding | 43 | GC12P008222 |
| PMPCA | Peptidase, Mitochondrial Processing Subunit Alpha | Protein Coding | 43 | GC09P136410 |
| DDHD1 | DDHD Domain Containing 1 | Protein Coding | 38 | GC14M053036 |
| TWIST1 | Twist Family BHLH Transcription Factor 1 | Protein Coding | 45 | GC07M019020 |
| AGPAT2 | 1-Acylglycerol-3-Phosphate O-Acyltransferase 2 | Protein Coding | 45 | GC09M136673 |
| CTSC | Cathepsin C | Protein Coding | 45 | GC11M088211 |
| NDUFB9 | NADH:Ubiquinone Oxidoreductase Subunit B9 | Protein Coding | 44 | GC08P124539 |
| ESR2 | Estrogen Receptor 2 | Protein Coding | 49 | GC14M064084 |
| GABRG2 | Gamma-Aminobutyric Acid Type A Receptor Subunit Gamma2 | Protein Coding | 46 | GC05P162000 |
| UBE3A | Ubiquitin Protein Ligase E3A | Protein Coding | 47 | GC15M025333 |
| ROBO3 | Roundabout Guidance Receptor 3 | Protein Coding | 43 | GC11P124865 |
| HOXB1 | Homeobox B1 | Protein Coding | 43 | GC17M048528 |
| BAG3 | BAG Cochaperone 3 | Protein Coding | 44 | GC10P119651 |
| TPH2 | Tryptophan Hydroxylase 2 | Protein Coding | 48 | GC12P071938 |
| GRK1 | G Protein-Coupled Receptor Kinase 1 | Protein Coding | 42 | GC13P113645 |
| NDUFAF3 | NADH:Ubiquinone Oxidoreductase Complex Assembly Factor 3 | Protein Coding | 40 | GC03P049020 |
| GRM7 | Glutamate Metabotropic Receptor 7 | Protein Coding | 44 | GC03P006770 |
| CHMP2B | Charged Multivesicular Body Protein 2B | Protein Coding | 44 | GC03P087277 |
| NDUFAF1 | NADH:Ubiquinone Oxidoreductase Complex Assembly Factor 1 | Protein Coding | 41 | GC15M041387 |
| CBL | Cbl Proto-Oncogene | Protein Coding | 50 | GC11P119206 |
| LMX1A | LIM Homeobox Transcription Factor 1 Alpha | Protein Coding | 42 | GC01M165171 |
| PDE6G | Phosphodiesterase 6G | Protein Coding | 44 | GC17M081650 |
| CDH2 | Cadherin 2 | Protein Coding | 50 | GC18M027950 |
| SLC7A14 | Solute Carrier Family 7 Member 14 | Protein Coding | 41 | GC03M170459 |
| CA4 | Carbonic Anhydrase 4 | Protein Coding | 47 | GC17P060149 |
| NSUN2 | NOP2/Sun RNA Methyltransferase 2 | Protein Coding | 42 | GC05M006599 |
| VCL | Vinculin | Protein Coding | 47 | GC10P073995 |
| NSD2 | Nuclear Receptor Binding SET Domain Protein 2 | Protein Coding | 35 | GC04P001872 |
| C12orf65 | Chromosome 12 Open Reading Frame 65 | Protein Coding | 36 | GC12P123232 |
| LZTR1 | Leucine Zipper Like Transcription Regulator 1 | Protein Coding | 43 | GC22P020983 |
| NPHP4 | Nephrocystin 4 | Protein Coding | 40 | GC01M005863 |
| FKRP | Fukutin Related Protein | Protein Coding | 40 | GC19P046746 |
| DCAF8 | DDB1 And CUL4 Associated Factor 8 | Protein Coding | 38 | GC01M160215 |
| IDS | Iduronate 2-Sulfatase | Protein Coding | 48 | GC0XM149476 |
| HTRA1 | HtrA Serine Peptidase 1 | Protein Coding | 43 | GC10P122461 |
| CSRP3 | Cysteine And Glycine Rich Protein 3 | Protein Coding | 41 | GC11M019160 |
| COX4I1 | Cytochrome C Oxidase Subunit 4I1 | Protein Coding | 44 | GC16P085798 |
| THPO | Thrombopoietin | Protein Coding | 42 | GC03M184371 |
| SLC18A3 | Solute Carrier Family 18 Member A3 | Protein Coding | 43 | GC10P049610 |
| PLAU | Plasminogen Activator, Urokinase | Protein Coding | 51 | GC10P073909 |
| MT-TN | Mitochondrially Encoded TRNA-Asn (AAU/C) | RNA Gene | 12 | GCMTM005659 |
| CLIP2 | CAP-Gly Domain Containing Linker Protein 2 | Protein Coding | 37 | GC07P074289 |
| MMP9 | Matrix Metallopeptidase 9 | Protein Coding | 52 | GC20P046008 |
| IL2 | Interleukin 2 | Protein Coding | 45 | GC04M122451 |
| COL4A2 | Collagen Type IV Alpha 2 Chain | Protein Coding | 44 | GC13P110305 |
| SLC39A14 | Solute Carrier Family 39 Member 14 | Protein Coding | 43 | GC08P022367 |
| ANKRD1 | Ankyrin Repeat Domain 1 | Protein Coding | 42 | GC10M090912 |
| ATXN2 | Ataxin 2 | Protein Coding | 42 | GC12M111443 |
| CD4 | CD4 Molecule | Protein Coding | 49 | GC12P006786 |
| MORC2 | MORC Family CW-Type Zinc Finger 2 | Protein Coding | 40 | GC22M030925 |
| PDE4D | Phosphodiesterase 4D | Protein Coding | 48 | GC05M058969 |
| GAA | Glucosidase Alpha, Acid | Protein Coding | 47 | GC17P080101 |
| ZNF41 | Zinc Finger Protein 41 | Protein Coding | 40 | GC0XM047444 |
| TNNC1 | Troponin C1, Slow Skeletal And Cardiac Type | Protein Coding | 45 | GC03M052452 |
| PRDM10 | PR/SET Domain 10 | Protein Coding | 34 | GC11M129899 |
| FBLN5 | Fibulin 5 | Protein Coding | 44 | GC14M091869 |
| MT-TC | Mitochondrially Encoded TRNA-Cys (UGU/C) | RNA Gene | 10 | GCMTM005763 |
| KCNA2 | Potassium Voltage-Gated Channel Subfamily A Member 2 | Protein Coding | 45 | GC01M110519 |
| SMN1 | Survival Of Motor Neuron 1, Telomeric | Protein Coding | 41 | GC05P070924 |
| TMEM106B | Transmembrane Protein 106B | Protein Coding | 38 | GC07P012217 |
| LPL | Lipoprotein Lipase | Protein Coding | 49 | GC08P019901 |
| DDX41 | DEAD-Box Helicase 41 | Protein Coding | 43 | GC05M177511 |
| DYSF | Dysferlin | Protein Coding | 43 | GC02P071453 |
| GRIA3 | Glutamate Ionotropic Receptor AMPA Type Subunit 3 | Protein Coding | 50 | GC0XP123184 |
| ABCC6 | ATP Binding Cassette Subfamily C Member 6 | Protein Coding | 45 | GC16M016148 |
| LEMD3 | LEM Domain Containing 3 | Protein Coding | 41 | GC12P065169 |
| FOXP3 | Forkhead Box P3 | Protein Coding | 46 | GC0XM049250 |
| NR3C2 | Nuclear Receptor Subfamily 3 Group C Member 2 | Protein Coding | 48 | GC04M148078 |
| LRP6 | LDL Receptor Related Protein 6 | Protein Coding | 48 | GC12M013893 |
| ALPL | Alkaline Phosphatase, Biomineralization Associated | Protein Coding | 50 | GC01P021508 |
| MT-TP | Mitochondrially Encoded TRNA-Pro (CCN) | RNA Gene | 10 | GCMTM015957 |
| BCOR | BCL6 Corepressor | Protein Coding | 41 | GC0XM040049 |
| TXNRD2 | Thioredoxin Reductase 2 | Protein Coding | 46 | GC22M019863 |
| SUCLA2 | Succinate-CoA Ligase ADP-Forming Subunit Beta | Protein Coding | 46 | GC13M047745 |
| MLXIPL | MLX Interacting Protein Like | Protein Coding | 41 | GC07M073593 |
| ODC1 | Ornithine Decarboxylase 1 | Protein Coding | 47 | GC02M010432 |
| PAFAH1B1 | Platelet Activating Factor Acetylhydrolase 1b Regulatory Subunit 1 | Protein Coding | 45 | GC17P002593 |
| TMPO | Thymopoietin | Protein Coding | 45 | GC12P098515 |
| POMC | Proopiomelanocortin | Protein Coding | 48 | GC02M025160 |
| CA2 | Carbonic Anhydrase 2 | Protein Coding | 51 | GC08P085463 |
| DLL1 | Delta Like Canonical Notch Ligand 1 | Protein Coding | 45 | GC06M170282 |
| TTC21B | Tetratricopeptide Repeat Domain 21B | Protein Coding | 38 | GC02M165905 |
| CALM2 | Calmodulin 2 | Protein Coding | 44 | GC02M047124 |
| ALK | ALK Receptor Tyrosine Kinase | Protein Coding | 51 | GC02M029156 |
| GTF2I | General Transcription Factor IIi | Protein Coding | 42 | GC07P074658 |
| WDR45 | WD Repeat Domain 45 | Protein Coding | 40 | GC0XM049074 |
| NR5A1 | Nuclear Receptor Subfamily 5 Group A Member 1 | Protein Coding | 50 | GC09M124481 |
| PLEC | Plectin | Protein Coding | 42 | GC08M143916 |
| HCFC1 | Host Cell Factor C1 | Protein Coding | 46 | GC0XM153947 |
| ZEB2 | Zinc Finger E-Box Binding Homeobox 2 | Protein Coding | 48 | GC02M144384 |
| IFT43 | Intraflagellar Transport 43 | Protein Coding | 37 | GC14P075902 |
| DIABLO | Diablo IAP-Binding Mitochondrial Protein | Protein Coding | 47 | GC12M122208 |
| KCNA5 | Potassium Voltage-Gated Channel Subfamily A Member 5 | Protein Coding | 45 | GC12P005043 |
| MIR125A | MicroRNA 125a | RNA Gene | 21 | GC19P051720 |
| ATP7A | ATPase Copper Transporting Alpha | Protein Coding | 45 | GC0XP077927 |
| HTRA2 | HtrA Serine Peptidase 2 | Protein Coding | 47 | GC02P074529 |
| C19orf12 | Chromosome 19 Open Reading Frame 12 | Protein Coding | 36 | GC19M029699 |
| RFC2 | Replication Factor C Subunit 2 | Protein Coding | 44 | GC07M074231 |
| TFAP2A | Transcription Factor AP-2 Alpha | Protein Coding | 47 | GC06M010393 |
| NKX2-1 | NK2 Homeobox 1 | Protein Coding | 46 | GC14M036516 |
| MBP | Myelin Basic Protein | Protein Coding | 44 | GC18M076978 |
| VAMP1 | Vesicle Associated Membrane Protein 1 | Protein Coding | 44 | GC12M006462 |
| EHHADH | Enoyl-CoA Hydratase And 3-Hydroxyacyl CoA Dehydrogenase | Protein Coding | 44 | GC03M185190 |
| ATOH1 | Atonal BHLH Transcription Factor 1 | Protein Coding | 38 | GC04P093828 |
| IL7R | Interleukin 7 Receptor | Protein Coding | 47 | GC05P035852 |
| PNKD | PNKD Metallo-Beta-Lactamase Domain Containing | Protein Coding | 42 | GC02P218270 |
| TPM2 | Tropomyosin 2 | Protein Coding | 44 | GC09M035672 |
| TCF7L2 | Transcription Factor 7 Like 2 | Protein Coding | 45 | GC10P112950 |
| NMNAT1 | Nicotinamide Nucleotide Adenylyltransferase 1 | Protein Coding | 48 | GC01P009944 |
| TBL2 | Transducin Beta Like 2 | Protein Coding | 39 | GC07M073568 |
| GTF2IRD1 | GTF2I Repeat Domain Containing 1 | Protein Coding | 42 | GC07P074461 |
| CD8A | CD8a Molecule | Protein Coding | 46 | GC02M086784 |
| CAPN3 | Calpain 3 | Protein Coding | 47 | GC15P042359 |
| RPS6KA3 | Ribosomal Protein S6 Kinase A3 | Protein Coding | 52 | GC0XM020149 |
| ATP8B1 | ATPase Phospholipid Transporting 8B1 | Protein Coding | 40 | GC18M057646 |
| CREB1 | CAMP Responsive Element Binding Protein 1 | Protein Coding | 48 | GC02P207529 |
| DOLK | Dolichol Kinase | Protein Coding | 39 | GC09M128945 |
| MIR204 | MicroRNA 204 | RNA Gene | 21 | GC09M070809 |
| DHDDS | Dehydrodolichyl Diphosphate Synthase Subunit | Protein Coding | 43 | GC01P026432 |
| PHGDH | Phosphoglycerate Dehydrogenase | Protein Coding | 49 | GC01P119660 |
| C4B | Complement C4B (Chido Blood Group) | Protein Coding | 41 | GC06P032014 |
| MYO9A | Myosin IXA | Protein Coding | 39 | GC15M071822 |
| KMT2B | Lysine Methyltransferase 2B | Protein Coding | 39 | GC19P038227 |
| CDKN1A | Cyclin Dependent Kinase Inhibitor 1A | Protein Coding | 50 | GC06P047460 |
| KCNMA1 | Potassium Calcium-Activated Channel Subfamily M Alpha 1 | Protein Coding | 49 | GC10M076869 |
| PNPLA8 | Patatin Like Phospholipase Domain Containing 8 | Protein Coding | 42 | GC07M108470 |
| PLIN1 | Perilipin 1 | Protein Coding | 44 | GC15M089664 |
| HNF1B | HNF1 Homeobox B | Protein Coding | 44 | GC17M037686 |
| SETD5 | SET Domain Containing 5 | Protein Coding | 38 | GC03P009402 |
| KCNH1 | Potassium Voltage-Gated Channel Subfamily H Member 1 | Protein Coding | 46 | GC01M210678 |
| GTF2H5 | General Transcription Factor IIH Subunit 5 | Protein Coding | 40 | GC06P158168 |
| ADCY10 | Adenylate Cyclase 10 | Protein Coding | 45 | GC01M167809 |
| PRL | Prolactin | Protein Coding | 44 | GC06M022230 |
| CDKN1C | Cyclin Dependent Kinase Inhibitor 1C | Protein Coding | 47 | GC11M002887 |
| SIRT1 | Sirtuin 1 | Protein Coding | 49 | GC10P067884 |
| ATOH7 | Atonal BHLH Transcription Factor 7 | Protein Coding | 39 | GC10M068230 |
| CHRNA4 | Cholinergic Receptor Nicotinic Alpha 4 Subunit | Protein Coding | 48 | GC20M063343 |
| ITGB4 | Integrin Subunit Beta 4 | Protein Coding | 48 | GC17P075721 |
| ACTA1 | Actin Alpha 1, Skeletal Muscle | Protein Coding | 47 | GC01M229431 |
| SALL2 | Spalt Like Transcription Factor 2 | Protein Coding | 42 | GC14M021521 |
| CREBBP | CREB Binding Protein | Protein Coding | 52 | GC16M003726 |
| CABP4 | Calcium Binding Protein 4 | Protein Coding | 37 | GC11P067453 |
| GNB1 | G Protein Subunit Beta 1 | Protein Coding | 45 | GC01M001785 |
| GHR | Growth Hormone Receptor | Protein Coding | 47 | GC05P042429 |
| MIR29A | MicroRNA 29a | RNA Gene | 21 | GC07M130876 |
| CNOT1 | CCR4-NOT Transcription Complex Subunit 1 | Protein Coding | 40 | GC16M058519 |
| ST3GAL5 | ST3 Beta-Galactoside Alpha-2,3-Sialyltransferase 5 | Protein Coding | 48 | GC02M085839 |
| COQ4 | Coenzyme Q4 | Protein Coding | 39 | GC09P128322 |
| HAND2 | Heart And Neural Crest Derivatives Expressed 2 | Protein Coding | 43 | GC04M173524 |
| TBL1XR1 | TBL1X Receptor 1 | Protein Coding | 44 | GC03M177019 |
| XK | X-Linked Kx Blood Group | Protein Coding | 40 | GC0XP037685 |
| FGF2 | Fibroblast Growth Factor 2 | Protein Coding | 47 | GC04P122826 |
| SLC25A46 | Solute Carrier Family 25 Member 46 | Protein Coding | 39 | GC05P110738 |
| RAB39B | RAB39B, Member RAS Oncogene Family | Protein Coding | 39 | GC0XM155259 |
| SORL1 | Sortilin Related Receptor 1 | Protein Coding | 43 | GC11P121452 |
| PNPLA1 | Patatin Like Phospholipase Domain Containing 1 | Protein Coding | 37 | GC06P047454 |
| CLCN4 | Chloride Voltage-Gated Channel 4 | Protein Coding | 43 | GC0XP010085 |
| PGM1 | Phosphoglucomutase 1 | Protein Coding | 48 | GC01P063593 |
| BGN | Biglycan | Protein Coding | 43 | GC0XP153494 |
| IL7 | Interleukin 7 | Protein Coding | 42 | GC08M078689 |
| VAC14 | VAC14 Component Of PIKFYVE Complex | Protein Coding | 43 | GC16M070688 |
| SDCCAG8 | SHH Signaling And Ciliogenesis Regulator SDCCAG8 | Protein Coding | 41 | GC01P243255 |
| IDH2 | Isocitrate Dehydrogenase (NADP(+)) 2 | Protein Coding | 52 | GC15M090083 |
| GJC3 | Gap Junction Protein Gamma 3 | Protein Coding | 39 | GC07M099923 |
| MAX | MYC Associated Factor X | Protein Coding | 48 | GC14M065009 |
| ADIPOQ | Adiponectin, C1Q And Collagen Domain Containing | Protein Coding | 45 | GC03P186842 |
| EIF2AK3 | Eukaryotic Translation Initiation Factor 2 Alpha Kinase 3 | Protein Coding | 48 | GC02M088637 |
| RBM8A | RNA Binding Motif Protein 8A | Protein Coding | 40 | GC01M145921 |
| MAOA | Monoamine Oxidase A | Protein Coding | 50 | GC0XP043654 |
| RAX2 | Retina And Anterior Neural Fold Homeobox 2 | Protein Coding | 35 | GC19M003769 |
| PLOD1 | Procollagen-Lysine,2-Oxoglutarate 5-Dioxygenase 1 | Protein Coding | 41 | GC01P011934 |
| CDK4 | Cyclin Dependent Kinase 4 | Protein Coding | 54 | GC12M057743 |
| MOCS1 | Molybdenum Cofactor Synthesis 1 | Protein Coding | 40 | GC06M039899 |
| COX8A | Cytochrome C Oxidase Subunit 8A | Protein Coding | 41 | GC11P063977 |
| MT-TL2 | Mitochondrially Encoded TRNA-Leu (CUN) 2 | RNA Gene | 13 | GCMTP012268 |
| SERPINA1 | Serpin Family A Member 1 | Protein Coding | 49 | GC14M094376 |
| CACNA2D4 | Calcium Voltage-Gated Channel Auxiliary Subunit Alpha2delta 4 | Protein Coding | 42 | GC12M001771 |
| MTTP | Microsomal Triglyceride Transfer Protein | Protein Coding | 44 | GC04P099563 |
| HSPA8 | Heat Shock Protein Family A (Hsp70) Member 8 | Protein Coding | 47 | GC11M123057 |
| ITGA2B | Integrin Subunit Alpha 2b | Protein Coding | 50 | GC17M044388 |
| RGS9BP | Regulator Of G Protein Signaling 9 Binding Protein | Protein Coding | 35 | GC19P032675 |
| NRTN | Neurturin | Protein Coding | 40 | GC19P005805 |
| TPM1 | Tropomyosin 1 | Protein Coding | 48 | GC15P073930 |
| ALDH18A1 | Aldehyde Dehydrogenase 18 Family Member A1 | Protein Coding | 45 | GC10M095605 |
| PRKD1 | Protein Kinase D1 | Protein Coding | 50 | GC14M029576 |
| SYT2 | Synaptotagmin 2 | Protein Coding | 43 | GC01M202559 |
| ARMS2 | Age-Related Maculopathy Susceptibility 2 | Protein Coding | 30 | GC10P122454 |
| BLOC1S1 | Biogenesis Of Lysosomal Organelles Complex 1 Subunit 1 | Protein Coding | 36 | GC12P055718 |
| CDKN1B | Cyclin Dependent Kinase Inhibitor 1B | Protein Coding | 48 | GC12P012716 |
| SGCA | Sarcoglycan Alpha | Protein Coding | 41 | GC17P050164 |
| TRIM8 | Tripartite Motif Containing 8 | Protein Coding | 38 | GC10P102643 |
| TNC | Tenascin C | Protein Coding | 48 | GC09M115019 |
| SGCD | Sarcoglycan Delta | Protein Coding | 45 | GC05P155686 |
| SLC19A3 | Solute Carrier Family 19 Member 3 | Protein Coding | 46 | GC02M227685 |
| SPTAN1 | Spectrin Alpha, Non-Erythrocytic 1 | Protein Coding | 47 | GC09P128552 |
| FN1 | Fibronectin 1 | Protein Coding | 50 | GC02M215360 |
| CDKN2B | Cyclin Dependent Kinase Inhibitor 2B | Protein Coding | 47 | GC09M022002 |
| ALG2 | ALG2 Alpha-1,3/1,6-Mannosyltransferase | Protein Coding | 41 | GC09M099216 |
| NDUFAF5 | NADH:Ubiquinone Oxidoreductase Complex Assembly Factor 5 | Protein Coding | 36 | GC20P013786 |
| PKD2 | Polycystin 2, Transient Receptor Potential Cation Channel | Protein Coding | 46 | GC04P088007 |
| MIR223 | MicroRNA 223 | RNA Gene | 21 | GC0XP066018 |
| NDUFS3 | NADH:Ubiquinone Oxidoreductase Core Subunit S3 | Protein Coding | 46 | GC11P047567 |
| KIF1A | Kinesin Family Member 1A | Protein Coding | 43 | GC02M240713 |
| CALCA | Calcitonin Related Polypeptide Alpha | Protein Coding | 43 | GC11M014945 |
| PNPO | Pyridoxamine 5'-Phosphate Oxidase | Protein Coding | 45 | GC17P047941 |
| IL17A | Interleukin 17A | Protein Coding | 42 | GC06P052186 |
| TPM3 | Tropomyosin 3 | Protein Coding | 47 | GC01M154127 |
| SLC25A24 | Solute Carrier Family 25 Member 24 | Protein Coding | 43 | GC01M108134 |
| TBX5 | T-Box Transcription Factor 5 | Protein Coding | 45 | GC12M114353 |
| WDR19 | WD Repeat Domain 19 | Protein Coding | 38 | GC04P039184 |
| APPL1 | Adaptor Protein, Phosphotyrosine Interacting With PH Domain And Leucine Zipper 1 | Protein Coding | 45 | GC03P057227 |
| SMARCB1 | SWI/SNF Related, Matrix Associated, Actin Dependent Regulator Of Chromatin, Subfamily B, Member 1 | Protein Coding | 45 | GC22P023786 |
| DDX3X | DEAD-Box Helicase 3 X-Linked | Protein Coding | 47 | GC0XP041333 |
| VCAN | Versican | Protein Coding | 47 | GC05P083471 |
| WDR4 | WD Repeat Domain 4 | Protein Coding | 38 | GC21M042843 |
| IL18 | Interleukin 18 | Protein Coding | 44 | GC11M112143 |
| MIR214 | MicroRNA 214 | RNA Gene | 20 | GC01M172234 |
| UBB | Ubiquitin B | Protein Coding | 43 | GC17P016380 |
| DNAI1 | Dynein Axonemal Intermediate Chain 1 | Protein Coding | 41 | GC09P034457 |
| ETFB | Electron Transfer Flavoprotein Subunit Beta | Protein Coding | 45 | GC19M051345 |
| DNASE1L3 | Deoxyribonuclease 1 Like 3 | Protein Coding | 43 | GC03M058192 |
| SMARCA2 | SWI/SNF Related, Matrix Associated, Actin Dependent Regulator Of Chromatin, Subfamily A, Member 2 | Protein Coding | 49 | GC09P001980 |
| PSTPIP1 | Proline-Serine-Threonine Phosphatase Interacting Protein 1 | Protein Coding | 45 | GC15P076993 |
| MIR20A | MicroRNA 20a | RNA Gene | 19 | GC13P091434 |
| NEUROD1 | Neuronal Differentiation 1 | Protein Coding | 44 | GC02M181673 |
| GDI1 | GDP Dissociation Inhibitor 1 | Protein Coding | 43 | GC0XP154436 |
| CLCN1 | Chloride Voltage-Gated Channel 1 | Protein Coding | 44 | GC07P143316 |
| TBX20 | T-Box Transcription Factor 20 | Protein Coding | 41 | GC07M035237 |
| KCNJ18 | Potassium Inwardly Rectifying Channel Subfamily J Member 18 | Protein Coding | 26 | GC17P026827 |
| GATA2 | GATA Binding Protein 2 | Protein Coding | 47 | GC03M128479 |
| CD28 | CD28 Molecule | Protein Coding | 47 | GC02P203706 |
| IGF1R | Insulin Like Growth Factor 1 Receptor | Protein Coding | 54 | GC15P098648 |
| PERP | P53 Apoptosis Effector Related To PMP22 | Protein Coding | 41 | GC06M138088 |
| SLC9A1 | Solute Carrier Family 9 Member A1 | Protein Coding | 51 | GC01M027109 |
| GSTM1 | Glutathione S-Transferase Mu 1 | Protein Coding | 41 | GC01P109687 |
| KRT14 | Keratin 14 | Protein Coding | 47 | GC17M041582 |
| ICAM1 | Intercellular Adhesion Molecule 1 | Protein Coding | 50 | GC19P010270 |
| OCLN | Occludin | Protein Coding | 44 | GC05P069492 |
| PQBP1 | Polyglutamine Binding Protein 1 | Protein Coding | 40 | GC0XP048890 |
| ACADL | Acyl-CoA Dehydrogenase Long Chain | Protein Coding | 43 | GC02M210187 |
| TUBA1A | Tubulin Alpha 1a | Protein Coding | 48 | GC12M049184 |
| NPRL3 | NPR3 Like, GATOR1 Complex Subunit | Protein Coding | 38 | GC16M000084 |
| IREB2 | Iron Responsive Element Binding Protein 2 | Protein Coding | 43 | GC15P078437 |
| TMEM127 | Transmembrane Protein 127 | Protein Coding | 37 | GC02M096248 |
| POMT1 | Protein O-Mannosyltransferase 1 | Protein Coding | 45 | GC09P131502 |
| NDUFS1 | NADH:Ubiquinone Oxidoreductase Core Subunit S1 | Protein Coding | 45 | GC02M206114 |
| HPRT1 | Hypoxanthine Phosphoribosyltransferase 1 | Protein Coding | 48 | GC0XP134460 |
| PET117 | PET117 Cytochrome C Oxidase Chaperone | Protein Coding | 29 | GC20P018119 |
| RHOBTB2 | Rho Related BTB Domain Containing 2 | Protein Coding | 40 | GC08P022987 |
| SGCG | Sarcoglycan Gamma | Protein Coding | 42 | GC13P023160 |
| OAT | Ornithine Aminotransferase | Protein Coding | 47 | GC10M124397 |
| GAPDH | Glyceraldehyde-3-Phosphate Dehydrogenase | Protein Coding | 48 | GC12P008161 |
| RETN | Resistin | Protein Coding | 43 | GC19P007669 |
| MDH2 | Malate Dehydrogenase 2 | Protein Coding | 48 | GC07P076048 |
| HIF1A | Hypoxia Inducible Factor 1 Subunit Alpha | Protein Coding | 47 | GC14P061695 |
| RUNX1 | RUNX Family Transcription Factor 1 | Protein Coding | 48 | GC21M034787 |
| SCARB2 | Scavenger Receptor Class B Member 2 | Protein Coding | 44 | GC04M076158 |
| GMPPA | GDP-Mannose Pyrophosphorylase A | Protein Coding | 42 | GC02P219498 |
| PAH | Phenylalanine Hydroxylase | Protein Coding | 48 | GC12M102836 |
| CASP1 | Caspase 1 | Protein Coding | 50 | GC11M105025 |
| GSK3B | Glycogen Synthase Kinase 3 Beta | Protein Coding | 50 | GC03M119821 |
| XYLT1 | Xylosyltransferase 1 | Protein Coding | 43 | GC16M017101 |
| PLAT | Plasminogen Activator, Tissue Type | Protein Coding | 49 | GC08M042174 |
| NDUFS6 | NADH:Ubiquinone Oxidoreductase Subunit S6 | Protein Coding | 43 | GC05P001801 |
| IDH1 | Isocitrate Dehydrogenase (NADP(+)) 1 | Protein Coding | 52 | GC02M208236 |
| TERT | Telomerase Reverse Transcriptase | Protein Coding | 51 | GC05M001253 |
| TNFRSF13B | TNF Receptor Superfamily Member 13B | Protein Coding | 46 | GC17M016929 |
| MPLKIP | M-Phase Specific PLK1 Interacting Protein | Protein Coding | 36 | GC07M040126 |
| IFIH1 | Interferon Induced With Helicase C Domain 1 | Protein Coding | 47 | GC02M162267 |
| RTN4IP1 | Reticulon 4 Interacting Protein 1 | Protein Coding | 41 | GC06M106571 |
| SARDH | Sarcosine Dehydrogenase | Protein Coding | 42 | GC09M133663 |
| S100B | S100 Calcium Binding Protein B | Protein Coding | 45 | GC21M047431 |
| RGS9 | Regulator Of G Protein Signaling 9 | Protein Coding | 44 | GC17P065137 |
| MPL | MPL Proto-Oncogene, Thrombopoietin Receptor | Protein Coding | 48 | GC01P043337 |
| ACVR1 | Activin A Receptor Type 1 | Protein Coding | 51 | GC02M157736 |
| HIBCH | 3-Hydroxyisobutyryl-CoA Hydrolase | Protein Coding | 43 | GC02M190189 |
| LITAF | Lipopolysaccharide Induced TNF Factor | Protein Coding | 44 | GC16M011547 |
| TRAK1 | Trafficking Kinesin Protein 1 | Protein Coding | 40 | GC03P042016 |
| MAP3K20 | Mitogen-Activated Protein Kinase Kinase Kinase 20 | Protein Coding | 39 | GC02P173076 |
| MIR483 | MicroRNA 483 | RNA Gene | 18 | GC11M002188 |
| ELAC2 | ElaC Ribonuclease Z 2 | Protein Coding | 42 | GC17M012991 |
| ACY1 | Aminoacylase 1 | Protein Coding | 46 | GC03P051983 |
| PC | Pyruvate Carboxylase | Protein Coding | 47 | GC11M066848 |
| DRD2 | Dopamine Receptor D2 | Protein Coding | 50 | GC11M113409 |
| VIM | Vimentin | Protein Coding | 50 | GC10P017227 |
| IGFBP3 | Insulin Like Growth Factor Binding Protein 3 | Protein Coding | 45 | GC07M045912 |
| LARS1 | Leucyl-TRNA Synthetase 1 | Protein Coding | 36 | GC05M146114 |
| MYOT | Myotilin | Protein Coding | 41 | GC05P137867 |
| SRSF2 | Serine And Arginine Rich Splicing Factor 2 | Protein Coding | 40 | GC17M076734 |
| IL1RAPL1 | Interleukin 1 Receptor Accessory Protein Like 1 | Protein Coding | 41 | GC0XP028605 |
| FLAD1 | Flavin Adenine Dinucleotide Synthetase 1 | Protein Coding | 41 | GC01P154983 |
| COX14 | Cytochrome C Oxidase Assembly Factor COX14 | Protein Coding | 37 | GC12P050111 |
| KCNJ6 | Potassium Inwardly Rectifying Channel Subfamily J Member 6 | Protein Coding | 46 | GC21M037607 |
| REEP1 | Receptor Accessory Protein 1 | Protein Coding | 39 | GC02M086213 |
| TMEM231 | Transmembrane Protein 231 | Protein Coding | 37 | GC16M075536 |
| RAI1 | Retinoic Acid Induced 1 | Protein Coding | 40 | GC17P017682 |
| ASXL1 | ASXL Transcriptional Regulator 1 | Protein Coding | 43 | GC20P032359 |
| MYC | MYC Proto-Oncogene, BHLH Transcription Factor | Protein Coding | 51 | GC08P127735 |
| CASP9 | Caspase 9 | Protein Coding | 48 | GC01M015491 |
| CNKSR2 | Connector Enhancer Of Kinase Suppressor Of Ras 2 | Protein Coding | 39 | GC0XP021392 |
| NR0B1 | Nuclear Receptor Subfamily 0 Group B Member 1 | Protein Coding | 46 | GC0XM030304 |
| NPTX2 | Neuronal Pentraxin 2 | Protein Coding | 39 | GC07P098620 |
| TG | Thyroglobulin | Protein Coding | 42 | GC08P132866 |
| HCCS | Holocytochrome C Synthase | Protein Coding | 41 | GC0XP011111 |
| COL5A2 | Collagen Type V Alpha 2 Chain | Protein Coding | 41 | GC02M189031 |
| GOSR2 | Golgi SNAP Receptor Complex Member 2 | Protein Coding | 43 | GC17P046924 |
| PNP | Purine Nucleoside Phosphorylase | Protein Coding | 47 | GC14P020468 |
| FCGR3B | Fc Fragment Of IgG Receptor IIIb | Protein Coding | 42 | GC01M161623 |
| KMT2C | Lysine Methyltransferase 2C | Protein Coding | 41 | GC07M152134 |
| PROP1 | PROP Paired-Like Homeobox 1 | Protein Coding | 40 | GC05M177992 |
| FOXRED1 | FAD Dependent Oxidoreductase Domain Containing 1 | Protein Coding | 40 | GC11P126269 |
| ATL1 | Atlastin GTPase 1 | Protein Coding | 41 | GC14P050532 |
| KCNJ16 | Potassium Inwardly Rectifying Channel Subfamily J Member 16 | Protein Coding | 39 | GC17P070053 |
| MCIDAS | Multiciliate Differentiation And DNA Synthesis Associated Cell Cycle Protein | Protein Coding | 29 | GC05M055219 |
| FHL2 | Four And A Half LIM Domains 2 | Protein Coding | 45 | GC02M105343 |
| RPS26 | Ribosomal Protein S26 | Protein Coding | 42 | GC12P056043 |
| IGBP1 | Immunoglobulin Binding Protein 1 | Protein Coding | 41 | GC0XP070133 |
| PDE1C | Phosphodiesterase 1C | Protein Coding | 45 | GC07M031616 |
| NOS2 | Nitric Oxide Synthase 2 | Protein Coding | 49 | GC17M027756 |
| ADPRS | ADP-Ribosylserine Hydrolase | Protein Coding | 30 | GC01P036089 |
| IL13 | Interleukin 13 | Protein Coding | 44 | GC05P132656 |
| UQCRFS1 | Ubiquinol-Cytochrome C Reductase, Rieske Iron-Sulfur Polypeptide 1 | Protein Coding | 45 | GC19M029205 |
| ZIC2 | Zic Family Member 2 | Protein Coding | 42 | GC13P099981 |
| TPO | Thyroid Peroxidase | Protein Coding | 48 | GC02P001374 |
| LRSAM1 | Leucine Rich Repeat And Sterile Alpha Motif Containing 1 | Protein Coding | 39 | GC09P127451 |
| PPOX | Protoporphyrinogen Oxidase | Protein Coding | 43 | GC01P161167 |
| NODAL | Nodal Growth Differentiation Factor | Protein Coding | 42 | GC10M070431 |
| CHRNA2 | Cholinergic Receptor Nicotinic Alpha 2 Subunit | Protein Coding | 46 | GC08M027459 |
| DISP1 | Dispatched RND Transporter Family Member 1 | Protein Coding | 37 | GC01P222814 |
| MFSD8 | Major Facilitator Superfamily Domain Containing 8 | Protein Coding | 37 | GC04M127917 |
| APRT | Adenine Phosphoribosyltransferase | Protein Coding | 47 | GC16M088810 |
| RNASEH2B | Ribonuclease H2 Subunit B | Protein Coding | 37 | GC13P050909 |
| TSEN54 | TRNA Splicing Endonuclease Subunit 54 | Protein Coding | 38 | GC17P075515 |
| GTF2E2 | General Transcription Factor IIE Subunit 2 | Protein Coding | 43 | GC08M030578 |
| ALDH7A1 | Aldehyde Dehydrogenase 7 Family Member A1 | Protein Coding | 47 | GC05M126541 |
| ARSH | Arylsulfatase Family Member H | Protein Coding | 34 | GC0XP003006 |
| REN | Renin | Protein Coding | 48 | GC01M204154 |
| NEBL | Nebulette | Protein Coding | 39 | GC10M020779 |
| MRAS | Muscle RAS Oncogene Homolog | Protein Coding | 45 | GC03P138347 |
| SLC39A8 | Solute Carrier Family 39 Member 8 | Protein Coding | 43 | GC04M102252 |
| RNASEH2C | Ribonuclease H2 Subunit C | Protein Coding | 39 | GC11M065714 |
| PES1 | Pescadillo Ribosomal Biogenesis Factor 1 | Protein Coding | 38 | GC22M030576 |
| BICD2 | BICD Cargo Adaptor 2 | Protein Coding | 40 | GC09M092711 |
| COA3 | Cytochrome C Oxidase Assembly Factor 3 | Protein Coding | 34 | GC17M042795 |
| TIMMDC1 | Translocase Of Inner Mitochondrial Membrane Domain Containing 1 | Protein Coding | 36 | GC03P119498 |
| LTBP2 | Latent Transforming Growth Factor Beta Binding Protein 2 | Protein Coding | 43 | GC14M074498 |
| MMP14 | Matrix Metallopeptidase 14 | Protein Coding | 51 | GC14P025277 |
| GREM1 | Gremlin 1, DAN Family BMP Antagonist | Protein Coding | 44 | GC15P032720 |
| FASTKD2 | FAST Kinase Domains 2 | Protein Coding | 38 | GC02P206766 |
| NTF3 | Neurotrophin 3 | Protein Coding | 43 | GC12P005432 |
| ADA2 | Adenosine Deaminase 2 | Protein Coding | 34 | GC22M017179 |
| KDM4C | Lysine Demethylase 4C | Protein Coding | 40 | GC09P006720 |
| TPH1 | Tryptophan Hydroxylase 1 | Protein Coding | 44 | GC11M018040 |
| PTPRC | Protein Tyrosine Phosphatase Receptor Type C | Protein Coding | 51 | GC01P198607 |
| GPT | Glutamic--Pyruvic Transaminase | Protein Coding | 41 | GC08P144502 |
| ICOSLG | Inducible T Cell Costimulator Ligand | Protein Coding | 39 | GC21M044222 |
| TFAP2B | Transcription Factor AP-2 Beta | Protein Coding | 44 | GC06P050894 |
| NPC2 | NPC Intracellular Cholesterol Transporter 2 | Protein Coding | 41 | GC14M074476 |
| AMACR | Alpha-Methylacyl-CoA Racemase | Protein Coding | 45 | GC05M033986 |
| NUBPL | Nucleotide Binding Protein Like | Protein Coding | 39 | GC14P031489 |
| MIP | Major Intrinsic Protein Of Lens Fiber | Protein Coding | 41 | GC12M056449 |
| IFNA1 | Interferon Alpha 1 | Protein Coding | 39 | GC09P021494 |
| MIF | Macrophage Migration Inhibitory Factor | Protein Coding | 49 | GC22P023894 |
| MGP | Matrix Gla Protein | Protein Coding | 42 | GC12M014881 |
| MIR199B | MicroRNA 199b | RNA Gene | 19 | GC09M128244 |
| ATXN8OS | ATXN8 Opposite Strand LncRNA | RNA Gene | 26 | GC13P070107 |
| MAOB | Monoamine Oxidase B | Protein Coding | 43 | GC0XM043766 |
| FLVCR1 | FLVCR Heme Transporter 1 | Protein Coding | 40 | GC01P212858 |
| LETM1 | Leucine Zipper And EF-Hand Containing Transmembrane Protein 1 | Protein Coding | 41 | GC04M001781 |
| STX1A | Syntaxin 1A | Protein Coding | 47 | GC07M073700 |
| MIR144 | MicroRNA 144 | RNA Gene | 16 | GC17M029965 |
| CPLX1 | Complexin 1 | Protein Coding | 43 | GC04M000784 |
| MT-TT | Mitochondrially Encoded TRNA-Thr (ACN) | RNA Gene | 15 | GCMTP015890 |
| MATR3 | Matrin 3 | Protein Coding | 41 | GC05P139274 |
| GATA6 | GATA Binding Protein 6 | Protein Coding | 47 | GC18P022169 |
| PKHD1 | PKHD1 Ciliary IPT Domain Containing Fibrocystin/Polyductin | Protein Coding | 39 | GC06M051588 |
| SOBP | Sine Oculis Binding Protein Homolog | Protein Coding | 38 | GC06P107489 |
| ENO2 | Enolase 2 | Protein Coding | 47 | GC12P006913 |
| STAT5B | Signal Transducer And Activator Of Transcription 5B | Protein Coding | 49 | GC17M042199 |
| ECHS1 | Enoyl-CoA Hydratase, Short Chain 1 | Protein Coding | 47 | GC10M133362 |
| TRPV3 | Transient Receptor Potential Cation Channel Subfamily V Member 3 | Protein Coding | 43 | GC17M003515 |
| CXCR4 | C-X-C Motif Chemokine Receptor 4 | Protein Coding | 52 | GC02M136114 |
| CIITA | Class II Major Histocompatibility Complex Transactivator | Protein Coding | 45 | GC16P010879 |
| EHMT1 | Euchromatic Histone Lysine Methyltransferase 1 | Protein Coding | 45 | GC09P137618 |
| NDUFAF4 | NADH:Ubiquinone Oxidoreductase Complex Assembly Factor 4 | Protein Coding | 42 | GC06M096889 |
| MRPS7 | Mitochondrial Ribosomal Protein S7 | Protein Coding | 39 | GC17P075262 |
| PLA2G2A | Phospholipase A2 Group IIA | Protein Coding | 45 | GC01M019975 |
| DNAH5 | Dynein Axonemal Heavy Chain 5 | Protein Coding | 40 | GC05M013745 |
| JUN | Jun Proto-Oncogene, AP-1 Transcription Factor Subunit | Protein Coding | 49 | GC01M058780 |
| ADAMTSL1 | ADAMTS Like 1 | Protein Coding | 41 | GC09P017906 |
| TGIF1 | TGFB Induced Factor Homeobox 1 | Protein Coding | 45 | GC18P003411 |
| ACADS | Acyl-CoA Dehydrogenase Short Chain | Protein Coding | 46 | GC12P120843 |
| COX5A | Cytochrome C Oxidase Subunit 5A | Protein Coding | 43 | GC15M074919 |
| GH1 | Growth Hormone 1 | Protein Coding | 44 | GC17M063917 |
| RNF113A | Ring Finger Protein 113A | Protein Coding | 37 | GC0XM119870 |
| UBA5 | Ubiquitin Like Modifier Activating Enzyme 5 | Protein Coding | 43 | GC03P132654 |
| TFAM | Transcription Factor A, Mitochondrial | Protein Coding | 43 | GC10P058385 |
| SERPINE1 | Serpin Family E Member 1 | Protein Coding | 50 | GC07P101127 |
| CHST3 | Carbohydrate Sulfotransferase 3 | Protein Coding | 42 | GC10P071964 |
| MIR155 | MicroRNA 155 | RNA Gene | 18 | GC21P025573 |
| FLII | FLII Actin Remodeling Protein | Protein Coding | 41 | GC17M018244 |
| FBXL4 | F-Box And Leucine Rich Repeat Protein 4 | Protein Coding | 39 | GC06M098868 |
| DNAJC6 | DnaJ Heat Shock Protein Family (Hsp40) Member C6 | Protein Coding | 43 | GC01P065248 |
| CLCN2 | Chloride Voltage-Gated Channel 2 | Protein Coding | 45 | GC03M184346 |
| NDUFA6 | NADH:Ubiquinone Oxidoreductase Subunit A6 | Protein Coding | 44 | GC22M042085 |
| ZNF335 | Zinc Finger Protein 335 | Protein Coding | 37 | GC20M045948 |
| MASP1 | Mannan Binding Lectin Serine Peptidase 1 | Protein Coding | 46 | GC03M187216 |
| MIR9-1 | MicroRNA 9-1 | RNA Gene | 20 | GC01M156420 |
| LTA | Lymphotoxin Alpha | Protein Coding | 42 | GC06P047303 |
| SLITRK1 | SLIT And NTRK Like Family Member 1 | Protein Coding | 40 | GC13M083877 |
| CYBB | Cytochrome B-245 Beta Chain | Protein Coding | 47 | GC0XP037780 |
| ACAN | Aggrecan | Protein Coding | 45 | GC15P088813 |
| ACHE | Acetylcholinesterase (Cartwright Blood Group) | Protein Coding | 45 | GC07M100889 |
| TMEM70 | Transmembrane Protein 70 | Protein Coding | 38 | GC08P073972 |
| SLC12A2 | Solute Carrier Family 12 Member 2 | Protein Coding | 46 | GC05P128083 |
| GALE | UDP-Galactose-4-Epimerase | Protein Coding | 45 | GC01M023795 |
| KLHL24 | Kelch Like Family Member 24 | Protein Coding | 35 | GC03P183635 |
| UNC13A | Unc-13 Homolog A | Protein Coding | 40 | GC19M017602 |
| MAPK3 | Mitogen-Activated Protein Kinase 3 | Protein Coding | 49 | GC16M030117 |
| CSF3 | Colony Stimulating Factor 3 | Protein Coding | 40 | GC17P040015 |
| INF2 | Inverted Formin 2 | Protein Coding | 40 | GC14P104788 |
| LAMA2 | Laminin Subunit Alpha 2 | Protein Coding | 43 | GC06P128863 |
| PHF6 | PHD Finger Protein 6 | Protein Coding | 40 | GC0XP134373 |
| DOK7 | Docking Protein 7 | Protein Coding | 39 | GC04P003465 |
| MIR198 | MicroRNA 198 | RNA Gene | 16 | GC03M120395 |
| TRAPPC11 | Trafficking Protein Particle Complex 11 | Protein Coding | 36 | GC04P183659 |
| HMGCL | 3-Hydroxy-3-Methylglutaryl-CoA Lyase | Protein Coding | 46 | GC01M023801 |
| ATP7B | ATPase Copper Transporting Beta | Protein Coding | 47 | GC13M051930 |
| PDGFB | Platelet Derived Growth Factor Subunit B | Protein Coding | 50 | GC22M045657 |
| CFHR1 | Complement Factor H Related 1 | Protein Coding | 41 | GC01P196788 |
| NUP155 | Nucleoporin 155 | Protein Coding | 44 | GC05M037288 |
| GJC1 | Gap Junction Protein Gamma 1 | Protein Coding | 42 | GC17M044800 |
| MIR499A | MicroRNA 499a | RNA Gene | 21 | GC20P034990 |
| VPS13A | Vacuolar Protein Sorting 13 Homolog A | Protein Coding | 40 | GC09P077177 |
| SLC35A2 | Solute Carrier Family 35 Member A2 | Protein Coding | 40 | GC0XM048903 |
| PNKP | Polynucleotide Kinase 3'-Phosphatase | Protein Coding | 45 | GC19M049861 |
| ARHGDIA | Rho GDP Dissociation Inhibitor Alpha | Protein Coding | 47 | GC17M081867 |
| ADK | Adenosine Kinase | Protein Coding | 50 | GC10P074152 |
| BACE1 | Beta-Secretase 1 | Protein Coding | 47 | GC11M117285 |
| AGL | Amylo-Alpha-1, 6-Glucosidase, 4-Alpha-Glucanotransferase | Protein Coding | 45 | GC01P099850 |
| FLG | Filaggrin | Protein Coding | 40 | GC01M152274 |
| WWOX | WW Domain Containing Oxidoreductase | Protein Coding | 47 | GC16P078099 |
| TARS1 | Threonyl-TRNA Synthetase 1 | Protein Coding | 36 | GC05P033441 |
| IRF5 | Interferon Regulatory Factor 5 | Protein Coding | 48 | GC07P128937 |
| NPPB | Natriuretic Peptide B | Protein Coding | 44 | GC01M011858 |
| HSP90AA1 | Heat Shock Protein 90 Alpha Family Class A Member 1 | Protein Coding | 48 | GC14M102080 |
| CSF2 | Colony Stimulating Factor 2 | Protein Coding | 44 | GC05P132073 |
| ZFPM2 | Zinc Finger Protein, FOG Family Member 2 | Protein Coding | 41 | GC08P104590 |
| ITGB6 | Integrin Subunit Beta 6 | Protein Coding | 47 | GC02M160099 |
| FLCN | Folliculin | Protein Coding | 41 | GC17M017206 |
| GTPBP3 | GTP Binding Protein 3, Mitochondrial | Protein Coding | 40 | GC19P023303 |
| MIR146B | MicroRNA 146b | RNA Gene | 19 | GC10P102436 |
| MIR342 | MicroRNA 342 | RNA Gene | 19 | GC14P100109 |
| SIN3A | SIN3 Transcription Regulator Family Member A | Protein Coding | 45 | GC15M075369 |
| POMT2 | Protein O-Mannosyltransferase 2 | Protein Coding | 43 | GC14M077274 |
| NPY | Neuropeptide Y | Protein Coding | 45 | GC07P024290 |
| RHOD | Ras Homolog Family Member D | Protein Coding | 39 | GC11P067057 |
| CITED2 | Cbp/P300 Interacting Transactivator With Glu/Asp Rich Carboxy-Terminal Domain 2 | Protein Coding | 44 | GC06M139371 |
| SELL | Selectin L | Protein Coding | 42 | GC01M169690 |
| MT-RNR2 | Mitochondrially Encoded 16S RRNA | RNA Gene | 18 | GCMTP001674 |
| ERBB4 | Erb-B2 Receptor Tyrosine Kinase 4 | Protein Coding | 55 | GC02M211375 |
| PECAM1 | Platelet And Endothelial Cell Adhesion Molecule 1 | Protein Coding | 40 | GC17M064319 |
| ASPA | Aspartoacylase | Protein Coding | 44 | GC17P003472 |
| HLA-DPA1 | Major Histocompatibility Complex, Class II, DP Alpha 1 | Protein Coding | 40 | GC06M033064 |
| MC2R | Melanocortin 2 Receptor | Protein Coding | 47 | GC18M017331 |
| IL1R1 | Interleukin 1 Receptor Type 1 | Protein Coding | 45 | GC02P102136 |
| LAMP1 | Lysosomal Associated Membrane Protein 1 | Protein Coding | 43 | GC13P113297 |
| MIRLET7B | MicroRNA Let-7b | RNA Gene | 20 | GC22P046119 |
| SHOC2 | SHOC2 Leucine Rich Repeat Scaffold Protein | Protein Coding | 41 | GC10P110919 |
| CS | Citrate Synthase | Protein Coding | 44 | GC12M056271 |
| MAP2 | Microtubule Associated Protein 2 | Protein Coding | 42 | GC02P209424 |
| COL5A1 | Collagen Type V Alpha 1 Chain | Protein Coding | 45 | GC09P134641 |
| TSHZ1 | Teashirt Zinc Finger Homeobox 1 | Protein Coding | 40 | GC18P075210 |
| MALAT1 | Metastasis Associated Lung Adenocarcinoma Transcript 1 | RNA Gene | 24 | GC11P065806 |
| PLOD3 | Procollagen-Lysine,2-Oxoglutarate 5-Dioxygenase 3 | Protein Coding | 45 | GC07M101205 |
| CYP27A1 | Cytochrome P450 Family 27 Subfamily A Member 1 | Protein Coding | 47 | GC02P218781 |
| SCP2 | Sterol Carrier Protein 2 | Protein Coding | 47 | GC01P052927 |
| NES | Nestin | Protein Coding | 39 | GC01M156668 |
| HMBS | Hydroxymethylbilane Synthase | Protein Coding | 44 | GC11P119084 |
| ITPR1 | Inositol 1,4,5-Trisphosphate Receptor Type 1 | Protein Coding | 48 | GC03P004486 |
| UBQLN2 | Ubiquilin 2 | Protein Coding | 42 | GC0XP056563 |
| RPS27A | Ribosomal Protein S27a | Protein Coding | 43 | GC02P055231 |
| SLC4A4 | Solute Carrier Family 4 Member 4 | Protein Coding | 46 | GC04P071063 |
| GLYCTK | Glycerate Kinase | Protein Coding | 42 | GC03P052288 |
| WDTC1 | WD And Tetratricopeptide Repeats 1 | Protein Coding | 34 | GC01P027245 |
| NEK1 | NIMA Related Kinase 1 | Protein Coding | 43 | GC04M169393 |
| WARS1 | Tryptophanyl-TRNA Synthetase 1 | Protein Coding | 37 | GC14M100334 |
| NDUFA11 | NADH:Ubiquinone Oxidoreductase Subunit A11 | Protein Coding | 37 | GC19M005891 |
| AQP1 | Aquaporin 1 (Colton Blood Group) | Protein Coding | 45 | GC07P030911 |
| WAS | WASP Actin Nucleation Promoting Factor | Protein Coding | 48 | GC0XP048676 |
| MB | Myoglobin | Protein Coding | 43 | GC22M035606 |
| KIF1C | Kinesin Family Member 1C | Protein Coding | 42 | GC17P004998 |
| AQP5 | Aquaporin 5 | Protein Coding | 45 | GC12P049961 |
| AQP2 | Aquaporin 2 | Protein Coding | 46 | GC12P049950 |
| FGF23 | Fibroblast Growth Factor 23 | Protein Coding | 45 | GC12M004368 |
| AMELX | Amelogenin X-Linked | Protein Coding | 36 | GC0XP011293 |
| LIFR | LIF Receptor Subunit Alpha | Protein Coding | 47 | GC05M038475 |
| GUF1 | GUF1 Homolog, GTPase | Protein Coding | 37 | GC04P044680 |
| PHF21A | PHD Finger Protein 21A | Protein Coding | 41 | GC11M061099 |
| GNA11 | G Protein Subunit Alpha 11 | Protein Coding | 47 | GC19P003094 |
| BGLAP | Bone Gamma-Carboxyglutamate Protein | Protein Coding | 40 | GC01P156242 |
| MIR10B | MicroRNA 10b | RNA Gene | 21 | GC02P176150 |
| CANX | Calnexin | Protein Coding | 44 | GC05P179678 |
| PPARA | Peroxisome Proliferator Activated Receptor Alpha | Protein Coding | 45 | GC22P046150 |
| CRYBB2 | Crystallin Beta B2 | Protein Coding | 40 | GC22P025213 |
| GGT1 | Gamma-Glutamyltransferase 1 | Protein Coding | 46 | GC22P024927 |
| EPHA4 | EPH Receptor A4 | Protein Coding | 50 | GC02M221418 |
| CXCL12 | C-X-C Motif Chemokine Ligand 12 | Protein Coding | 45 | GC10M044294 |
| CDK8 | Cyclin Dependent Kinase 8 | Protein Coding | 47 | GC13P026254 |
| PABPN1 | Poly(A) Binding Protein Nuclear 1 | Protein Coding | 44 | GC14P025379 |
| TIMP1 | TIMP Metallopeptidase Inhibitor 1 | Protein Coding | 45 | GC0XP047583 |
| DNAH9 | Dynein Axonemal Heavy Chain 9 | Protein Coding | 39 | GC17P011598 |
| STAR | Steroidogenic Acute Regulatory Protein | Protein Coding | 46 | GC08M038145 |
| MAPK14 | Mitogen-Activated Protein Kinase 14 | Protein Coding | 51 | GC06P047451 |
| VDAC1 | Voltage Dependent Anion Channel 1 | Protein Coding | 45 | GC05M133975 |
| HACE1 | HECT Domain And Ankyrin Repeat Containing E3 Ubiquitin Protein Ligase 1 | Protein Coding | 41 | GC06M104728 |
| PLEKHM2 | Pleckstrin Homology And RUN Domain Containing M2 | Protein Coding | 35 | GC01P015691 |
| NAA50 | N-Alpha-Acetyltransferase 50, NatE Catalytic Subunit | Protein Coding | 37 | GC03M113716 |
| ATCAY | ATCAY Kinesin Light Chain Interacting Caytaxin | Protein Coding | 38 | GC19P003880 |
| PAM16 | Presequence Translocase Associated Motor 16 | Protein Coding | 36 | GC16M004332 |
| TGFBI | Transforming Growth Factor Beta Induced | Protein Coding | 45 | GC05P136027 |
| NCF1 | Neutrophil Cytosolic Factor 1 | Protein Coding | 48 | GC07P074773 |
| NEXMIF | Neurite Extension And Migration Factor | Protein Coding | 27 | GC0XM074733 |
| PVALB | Parvalbumin | Protein Coding | 39 | GC22M036800 |
| ADCYAP1 | Adenylate Cyclase Activating Polypeptide 1 | Protein Coding | 41 | GC18P000895 |
| NUS1 | NUS1 Dehydrodolichyl Diphosphate Synthase Subunit | Protein Coding | 39 | GC06P117675 |
| IL5 | Interleukin 5 | Protein Coding | 44 | GC05M132541 |
| COL13A1 | Collagen Type XIII Alpha 1 Chain | Protein Coding | 41 | GC10P069801 |
| CHKB | Choline Kinase Beta | Protein Coding | 45 | GC22M050578 |
| M6PR | Mannose-6-Phosphate Receptor, Cation Dependent | Protein Coding | 43 | GC12M008955 |
| RARS2 | Arginyl-TRNA Synthetase 2, Mitochondrial | Protein Coding | 42 | GC06M087514 |
| GJB5 | Gap Junction Protein Beta 5 | Protein Coding | 40 | GC01P034755 |
| NME8 | NME/NM23 Family Member 8 | Protein Coding | 40 | GC07P037889 |
| MSX2 | Msh Homeobox 2 | Protein Coding | 47 | GC05P174724 |
| OTULIN | OTU Deubiquitinase With Linear Linkage Specificity | Protein Coding | 38 | GC05P014667 |
| MIPEP | Mitochondrial Intermediate Peptidase | Protein Coding | 42 | GC13M023730 |
| KLF4 | Kruppel Like Factor 4 | Protein Coding | 45 | GC09M107484 |
| AP1S3 | Adaptor Related Protein Complex 1 Subunit Sigma 3 | Protein Coding | 40 | GC02M223751 |
| KCNJ12 | Potassium Inwardly Rectifying Channel Subfamily J Member 12 | Protein Coding | 43 | GC17P026750 |
| TIMM50 | Translocase Of Inner Mitochondrial Membrane 50 | Protein Coding | 38 | GC19P039480 |
| MAPK8 | Mitogen-Activated Protein Kinase 8 | Protein Coding | 50 | GC10P048306 |
| TBP | TATA-Box Binding Protein | Protein Coding | 48 | GC06P170554 |
| ARV1 | ARV1 Homolog, Fatty Acid Homeostasis Modulator | Protein Coding | 36 | GC01P230978 |
| PRKCB | Protein Kinase C Beta | Protein Coding | 47 | GC16P023892 |
| AIF1 | Allograft Inflammatory Factor 1 | Protein Coding | 39 | GC06P047304 |
| DYNC1I1 | Dynein Cytoplasmic 1 Intermediate Chain 1 | Protein Coding | 40 | GC07P095772 |
| CHGA | Chromogranin A | Protein Coding | 42 | GC14P092923 |
| SP1 | Sp1 Transcription Factor | Protein Coding | 44 | GC12P053380 |
| CXCL10 | C-X-C Motif Chemokine Ligand 10 | Protein Coding | 44 | GC04M076021 |
| MT-TA | Mitochondrially Encoded TRNA-Ala (GCN) | RNA Gene | 10 | GCMTM005589 |
| GJA3 | Gap Junction Protein Alpha 3 | Protein Coding | 41 | GC13M020139 |
| NEFH | Neurofilament Heavy | Protein Coding | 45 | GC22P029480 |
| DBH | Dopamine Beta-Hydroxylase | Protein Coding | 50 | GC09P133636 |
| CRYZL1 | Crystallin Zeta Like 1 | Protein Coding | 35 | GC21M033589 |
| KRIT1 | KRIT1 Ankyrin Repeat Containing | Protein Coding | 41 | GC07M092198 |
| TLR9 | Toll Like Receptor 9 | Protein Coding | 45 | GC03M052222 |
| PANK4 | Pantothenate Kinase 4 (Inactive) | Protein Coding | 40 | GC01M002508 |
| F3 | Coagulation Factor III, Tissue Factor | Protein Coding | 45 | GC01M094530 |
| WASHC5 | WASH Complex Subunit 5 | Protein Coding | 32 | GC08M128215 |
| TACR1 | Tachykinin Receptor 1 | Protein Coding | 45 | GC02M075010 |
| KCNE4 | Potassium Voltage-Gated Channel Subfamily E Regulatory Subunit 4 | Protein Coding | 36 | GC02P223051 |
| ABCG5 | ATP Binding Cassette Subfamily G Member 5 | Protein Coding | 44 | GC02M043806 |
| CAMK2G | Calcium/Calmodulin Dependent Protein Kinase II Gamma | Protein Coding | 47 | GC10M073812 |
| LGI1 | Leucine Rich Glioma Inactivated 1 | Protein Coding | 43 | GC10P093757 |
| SDHAF2 | Succinate Dehydrogenase Complex Assembly Factor 2 | Protein Coding | 41 | GC11P061430 |
| ABCB1 | ATP Binding Cassette Subfamily B Member 1 | Protein Coding | 51 | GC07M087504 |
| SELE | Selectin E | Protein Coding | 44 | GC01M169722 |
| PTGS2 | Prostaglandin-Endoperoxide Synthase 2 | Protein Coding | 48 | GC01M186640 |
| ABCC2 | ATP Binding Cassette Subfamily C Member 2 | Protein Coding | 47 | GC10P099782 |
| ANK3 | Ankyrin 3 | Protein Coding | 44 | GC10M060026 |
| NUP188 | Nucleoporin 188 | Protein Coding | 35 | GC09P128947 |
| ETHE1 | ETHE1 Persulfide Dioxygenase | Protein Coding | 42 | GC19M043506 |
| DEAF1 | DEAF1 Transcription Factor | Protein Coding | 40 | GC11M000644 |
| HNRNPA1 | Heterogeneous Nuclear Ribonucleoprotein A1 | Protein Coding | 45 | GC12P054280 |
| DNAH11 | Dynein Axonemal Heavy Chain 11 | Protein Coding | 41 | GC07P021543 |
| SLC9A6 | Solute Carrier Family 9 Member A6 | Protein Coding | 46 | GC0XP135985 |
| TAC1 | Tachykinin Precursor 1 | Protein Coding | 43 | GC07P097731 |
| EPO | Erythropoietin | Protein Coding | 41 | GC07P100720 |
| IL2RA | Interleukin 2 Receptor Subunit Alpha | Protein Coding | 50 | GC10M006010 |
| SGCE | Sarcoglycan Epsilon | Protein Coding | 42 | GC07M094585 |
| EYA3 | EYA Transcriptional Coactivator And Phosphatase 3 | Protein Coding | 37 | GC01M027970 |
| CD68 | CD68 Molecule | Protein Coding | 40 | GC17P007579 |
| IFNB1 | Interferon Beta 1 | Protein Coding | 41 | GC09M021077 |
| KCNQ1-AS1 | KCNQ1 Antisense RNA 1 | RNA Gene | 15 | GC11M002864 |
| EDC3 | Enhancer Of MRNA Decapping 3 | Protein Coding | 41 | GC15M074631 |
| BUB1B | BUB1 Mitotic Checkpoint Serine/Threonine Kinase B | Protein Coding | 49 | GC15P040161 |
| MDM2 | MDM2 Proto-Oncogene | Protein Coding | 52 | GC12P068808 |
| BMP7 | Bone Morphogenetic Protein 7 | Protein Coding | 45 | GC20M057168 |
| LAMA1 | Laminin Subunit Alpha 1 | Protein Coding | 45 | GC18M006941 |
| ALG10B | ALG10 Alpha-1,2-Glucosyltransferase B | Protein Coding | 33 | GC12P038316 |
| RIT1 | Ras Like Without CAAX 1 | Protein Coding | 45 | GC01M155897 |
| HOXA13 | Homeobox A13 | Protein Coding | 43 | GC07M027263 |
| PHOX2A | Paired Like Homeobox 2A | Protein Coding | 41 | GC11M072239 |
| PRICKLE2 | Prickle Planar Cell Polarity Protein 2 | Protein Coding | 39 | GC03M064079 |
| KANSL1 | KAT8 Regulatory NSL Complex Subunit 1 | Protein Coding | 39 | GC17M046031 |
| KDR | Kinase Insert Domain Receptor | Protein Coding | 53 | GC04M055078 |
| SLC17A5 | Solute Carrier Family 17 Member 5 | Protein Coding | 44 | GC06M073593 |
| CD34 | CD34 Molecule | Protein Coding | 43 | GC01M207880 |
| RAP1A | RAP1A, Member Of RAS Oncogene Family | Protein Coding | 47 | GC01P111542 |
| FIP1L1 | Factor Interacting With PAPOLA And CPSF1 | Protein Coding | 38 | GC04P053386 |
| TP73 | Tumor Protein P73 | Protein Coding | 45 | GC01P003652 |
| CNTF | Ciliary Neurotrophic Factor | Protein Coding | 41 | GC11P058622 |
| GLE1 | GLE1 RNA Export Mediator | Protein Coding | 40 | GC09P128504 |
| KCNN3 | Potassium Calcium-Activated Channel Subfamily N Member 3 | Protein Coding | 44 | GC01M154697 |
| OTC | Ornithine Carbamoyltransferase | Protein Coding | 47 | GC0XP038353 |
| SCN3A | Sodium Voltage-Gated Channel Alpha Subunit 3 | Protein Coding | 47 | GC02M165087 |
| RTN4 | Reticulon 4 | Protein Coding | 44 | GC02M054934 |
| CDKN2B-AS1 | CDKN2B Antisense RNA 1 | RNA Gene | 21 | GC09P021994 |
| CHRNB1 | Cholinergic Receptor Nicotinic Beta 1 Subunit | Protein Coding | 43 | GC17P008018 |
| LEMD2 | LEM Domain Nuclear Envelope Protein 2 | Protein Coding | 39 | GC06M033772 |
| HDAC9 | Histone Deacetylase 9 | Protein Coding | 46 | GC07P018086 |
| RAPSN | Receptor Associated Protein Of The Synapse | Protein Coding | 42 | GC11M061127 |
| KCNJ3 | Potassium Inwardly Rectifying Channel Subfamily J Member 3 | Protein Coding | 45 | GC02P154698 |
| NGFR | Nerve Growth Factor Receptor | Protein Coding | 45 | GC17P049495 |
| TAB2 | TGF-Beta Activated Kinase 1 (MAP3K7) Binding Protein 2 | Protein Coding | 47 | GC06P149218 |
| SDR9C7 | Short Chain Dehydrogenase/Reductase Family 9C Member 7 | Protein Coding | 39 | GC12M056923 |
| SDHAF1 | Succinate Dehydrogenase Complex Assembly Factor 1 | Protein Coding | 35 | GC19P035995 |
| CD44 | CD44 Molecule (Indian Blood Group) | Protein Coding | 47 | GC11P035139 |
| LRRC6 | Leucine Rich Repeat Containing 6 | Protein Coding | 37 | GC08M132570 |
| CCNF | Cyclin F | Protein Coding | 39 | GC16P002429 |
| SFTA3 | Surfactant Associated 3 | RNA Gene | 32 | GC14M036474 |
| OPA6 | Optic Atrophy 6 (Autosomal Recessive) | Genetic Locus | 3 | GC08U900809 |
| RSPH9 | Radial Spoke Head Component 9 | Protein Coding | 37 | GC06P047536 |
| LRP12 | LDL Receptor Related Protein 12 | Protein Coding | 40 | GC08M104489 |
| GM2A | GM2 Ganglioside Activator | Protein Coding | 43 | GC05P151229 |
| MIAT | Myocardial Infarction Associated Transcript | RNA Gene | 23 | GC22P026646 |
| U2AF1 | U2 Small Nuclear RNA Auxiliary Factor 1 | Protein Coding | 41 | GC21M043092 |
| TCF20 | Transcription Factor 20 | Protein Coding | 37 | GC22M042160 |
| YARS2 | Tyrosyl-TRNA Synthetase 2 | Protein Coding | 44 | GC12M032725 |
| CBSL | Cystathionine Beta-Synthase Like | Protein Coding | 17 | GC21M006445 |
| ENO1 | Enolase 1 | Protein Coding | 47 | GC01M008861 |
| BCL6 | BCL6 Transcription Repressor | Protein Coding | 45 | GC03M187721 |
| CD79A | CD79a Molecule | Protein Coding | 46 | GC19P041877 |
| CLU | Clusterin | Protein Coding | 46 | GC08M027596 |
| MAGI2 | Membrane Associated Guanylate Kinase, WW And PDZ Domain Containing 2 | Protein Coding | 43 | GC07M078017 |
| CTCF | CCCTC-Binding Factor | Protein Coding | 46 | GC16P067563 |
| FEV | FEV Transcription Factor, ETS Family Member | Protein Coding | 35 | GC02M218981 |
| TLR10 | Toll Like Receptor 10 | Protein Coding | 40 | GC04M038773 |
| BCL11A | BAF Chromatin Remodeling Complex Subunit BCL11A | Protein Coding | 43 | GC02M060451 |
| BCL2L1 | BCL2 Like 1 | Protein Coding | 47 | GC20M031664 |
| SUOX | Sulfite Oxidase | Protein Coding | 45 | GC12P055997 |
| AOC3 | Amine Oxidase Copper Containing 3 | Protein Coding | 44 | GC17P042851 |
| FKBP1B | FKBP Prolyl Isomerase 1B | Protein Coding | 40 | GC02P024033 |
| NCAM1 | Neural Cell Adhesion Molecule 1 | Protein Coding | 45 | GC11P112961 |
| GSTP1 | Glutathione S-Transferase Pi 1 | Protein Coding | 50 | GC11P067583 |
| FGF13 | Fibroblast Growth Factor 13 | Protein Coding | 41 | GC0XM138615 |
| GFI1 | Growth Factor Independent 1 Transcriptional Repressor | Protein Coding | 41 | GC01M092474 |
| SERPINF2 | Serpin Family F Member 2 | Protein Coding | 44 | GC17P001742 |
| SHBG | Sex Hormone Binding Globulin | Protein Coding | 40 | GC17P007613 |
| RPS3A | Ribosomal Protein S3A | Protein Coding | 41 | GC04P151099 |
| PIK3CG | Phosphatidylinositol-4,5-Bisphosphate 3-Kinase Catalytic Subunit Gamma | Protein Coding | 48 | GC07P106865 |
| CCDC141 | Coiled-Coil Domain Containing 141 | Protein Coding | 36 | GC02M178829 |
| PSMD4 | Proteasome 26S Subunit, Non-ATPase 4 | Protein Coding | 44 | GC01P151227 |
| ITGA4 | Integrin Subunit Alpha 4 | Protein Coding | 48 | GC02P181456 |
| HELLS | Helicase, Lymphoid Specific | Protein Coding | 45 | GC10P094501 |
| CHRND | Cholinergic Receptor Nicotinic Delta Subunit | Protein Coding | 42 | GC02P232525 |
| TIMM17A | Translocase Of Inner Mitochondrial Membrane 17A | Protein Coding | 40 | GC01P201955 |
| DNAAF6 | Dynein Axonemal Assembly Factor 6 | Protein Coding | 28 | GC0XP107207 |
| CHRNG | Cholinergic Receptor Nicotinic Gamma Subunit | Protein Coding | 40 | GC02P232539 |
| NDUFB10 | NADH:Ubiquinone Oxidoreductase Subunit B10 | Protein Coding | 41 | GC16P002430 |
| EZH2 | Enhancer Of Zeste 2 Polycomb Repressive Complex 2 Subunit | Protein Coding | 54 | GC07M148807 |
| FMN1 | Formin 1 | Protein Coding | 38 | GC15M032765 |
| HMGB1 | High Mobility Group Box 1 | Protein Coding | 44 | GC13M030456 |
| OPA8 | Optic Atrophy 8 (Autosomal Dominant) | Genetic Locus | 2 | GC16U902270 |
| SRA1 | Steroid Receptor RNA Activator 1 | Protein Coding | 37 | GC05M140537 |
| KCNJ4 | Potassium Inwardly Rectifying Channel Subfamily J Member 4 | Protein Coding | 44 | GC22M038426 |
| IGFBP2 | Insulin Like Growth Factor Binding Protein 2 | Protein Coding | 43 | GC02P216632 |
| DAG1 | Dystroglycan 1 | Protein Coding | 46 | GC03P049482 |
| HMGCR | 3-Hydroxy-3-Methylglutaryl-CoA Reductase | Protein Coding | 45 | GC05P075336 |
| RBFOX3 | RNA Binding Fox-1 Homolog 3 | Protein Coding | 35 | GC17M079089 |
| EFEMP2 | EGF Containing Fibulin Extracellular Matrix Protein 2 | Protein Coding | 43 | GC11M065867 |
| OXT | Oxytocin/Neurophysin I Prepropeptide | Protein Coding | 40 | GC20P003068 |
| ACTN4 | Actinin Alpha 4 | Protein Coding | 46 | GC19P038647 |
| BLK | BLK Proto-Oncogene, Src Family Tyrosine Kinase | Protein Coding | 51 | GC08P011486 |
| PRORP | Protein Only RNase P Catalytic Subunit | Protein Coding | 27 | GC14P035123 |
| LGALS4 | Galectin 4 | Protein Coding | 38 | GC19M042441 |
| FBXW7 | F-Box And WD Repeat Domain Containing 7 | Protein Coding | 44 | GC04M152321 |
| MLX | MAX Dimerization Protein MLX | Protein Coding | 43 | GC17P042567 |
| MTFP1 | Mitochondrial Fission Process 1 | Protein Coding | 32 | GC22P030426 |
| NAT9 | N-Acetyltransferase 9 (Putative) | Protein Coding | 37 | GC17M074770 |
| FRRS1L | Ferric Chelate Reductase 1 Like | Protein Coding | 34 | GC09M109130 |
| MIR24-1 | MicroRNA 24-1 | RNA Gene | 18 | GC09P095086 |
| IBSP | Integrin Binding Sialoprotein | Protein Coding | 37 | GC04P087799 |
| NRG1 | Neuregulin 1 | Protein Coding | 46 | GC08P031639 |
| TRH | Thyrotropin Releasing Hormone | Protein Coding | 42 | GC03P129974 |
| SLC18A2 | Solute Carrier Family 18 Member A2 | Protein Coding | 47 | GC10P117241 |
| AFP | Alpha Fetoprotein | Protein Coding | 45 | GC04P073431 |
| AAAS | Aladin WD Repeat Nucleoporin | Protein Coding | 41 | GC12M053307 |
| RAB5A | RAB5A, Member RAS Oncogene Family | Protein Coding | 45 | GC03P019963 |
| NUMA1 | Nuclear Mitotic Apparatus Protein 1 | Protein Coding | 43 | GC11M072002 |
| TBCD | Tubulin Folding Cofactor D | Protein Coding | 41 | GC17P082752 |
| IMMT | Inner Membrane Mitochondrial Protein | Protein Coding | 39 | GC02M086144 |
| TRPM6 | Transient Receptor Potential Cation Channel Subfamily M Member 6 | Protein Coding | 45 | GC09M074725 |
| KCNJ1 | Potassium Inwardly Rectifying Channel Subfamily J Member 1 | Protein Coding | 47 | GC11M128741 |
| HEY2 | Hes Related Family BHLH Transcription Factor With YRPW Motif 2 | Protein Coding | 38 | GC06P125730 |
| CCR5 | C-C Motif Chemokine Receptor 5 | Protein Coding | 46 | GC03P046383 |
| OPN4 | Opsin 4 | Protein Coding | 40 | GC10P086654 |
| OPN1SW | Opsin 1, Short Wave Sensitive | Protein Coding | 40 | GC07M128772 |
| NCOA7 | Nuclear Receptor Coactivator 7 | Protein Coding | 36 | GC06P125781 |
| CD1C | CD1c Molecule | Protein Coding | 39 | GC01P158289 |
| GRB2 | Growth Factor Receptor Bound Protein 2 | Protein Coding | 49 | GC17M075318 |
| VAPB | VAMP Associated Protein B And C | Protein Coding | 45 | GC20P058389 |
| ABCB4 | ATP Binding Cassette Subfamily B Member 4 | Protein Coding | 45 | GC07M087401 |
| COQ9 | Coenzyme Q9 | Protein Coding | 41 | GC16P057447 |
| FGF4 | Fibroblast Growth Factor 4 | Protein Coding | 44 | GC11M069762 |
| CCL4 | C-C Motif Chemokine Ligand 4 | Protein Coding | 40 | GC17P036103 |
| TOP1 | DNA Topoisomerase I | Protein Coding | 48 | GC20P041028 |
| SLC17A6 | Solute Carrier Family 17 Member 6 | Protein Coding | 40 | GC11P022359 |
| MC4R | Melanocortin 4 Receptor | Protein Coding | 45 | GC18M060371 |
| EXO1 | Exonuclease 1 | Protein Coding | 43 | GC01P241847 |
| KCND2 | Potassium Voltage-Gated Channel Subfamily D Member 2 | Protein Coding | 43 | GC07P120273 |
| TIMM23 | Translocase Of Inner Mitochondrial Membrane 23 | Protein Coding | 31 | GC10P045972 |
| TIMM23B | Translocase Of Inner Mitochondrial Membrane 23 Homolog B | Protein Coding | 24 | GC10P049942 |
| HDAC1 | Histone Deacetylase 1 | Protein Coding | 49 | GC01P032292 |
| F2R | Coagulation Factor II Thrombin Receptor | Protein Coding | 46 | GC05P076716 |
| PCCB | Propionyl-CoA Carboxylase Subunit Beta | Protein Coding | 45 | GC03P136250 |
| MEF2C | Myocyte Enhancer Factor 2C | Protein Coding | 48 | GC05M088718 |
| TUSC3 | Tumor Suppressor Candidate 3 | Protein Coding | 41 | GC08P015417 |
| TSHR | Thyroid Stimulating Hormone Receptor | Protein Coding | 47 | GC14P080954 |
| MIR221 | MicroRNA 221 | RNA Gene | 20 | GC0XM045746 |
| PARL | Presenilin Associated Rhomboid Like | Protein Coding | 41 | GC03M183825 |
| CALB1 | Calbindin 1 | Protein Coding | 41 | GC08M090058 |
| CLPX | Caseinolytic Mitochondrial Matrix Peptidase Chaperone Subunit X | Protein Coding | 39 | GC15M065148 |
| LTBP4 | Latent Transforming Growth Factor Beta Binding Protein 4 | Protein Coding | 40 | GC19P040592 |
| RIPPLY3 | Ripply Transcriptional Repressor 3 | Protein Coding | 30 | GC21P037006 |
| EN1 | Engrailed Homeobox 1 | Protein Coding | 38 | GC02M118842 |
| CD40 | CD40 Molecule | Protein Coding | 48 | GC20P046118 |
| CD27 | CD27 Molecule | Protein Coding | 45 | GC12P008144 |
| FDFT1 | Farnesyl-Diphosphate Farnesyltransferase 1 | Protein Coding | 44 | GC08P011795 |
| SLC25A17 | Solute Carrier Family 25 Member 17 | Protein Coding | 39 | GC22M045405 |
| ABCD3 | ATP Binding Cassette Subfamily D Member 3 | Protein Coding | 44 | GC01P094418 |
| SMAD2 | SMAD Family Member 2 | Protein Coding | 47 | GC18M047809 |
| PIGA | Phosphatidylinositol Glycan Anchor Biosynthesis Class A | Protein Coding | 44 | GC0XM015319 |
| G6PD | Glucose-6-Phosphate Dehydrogenase | Protein Coding | 50 | GC0XM154531 |
| GAMT | Guanidinoacetate N-Methyltransferase | Protein Coding | 46 | GC19M001397 |
| CLCN5 | Chloride Voltage-Gated Channel 5 | Protein Coding | 42 | GC0XP049922 |
| KCNN4 | Potassium Calcium-Activated Channel Subfamily N Member 4 | Protein Coding | 48 | GC19M043767 |
| CYP21A2 | Cytochrome P450 Family 21 Subfamily A Member 2 | Protein Coding | 45 | GC06P047333 |
| TNFSF10 | TNF Superfamily Member 10 | Protein Coding | 46 | GC03M172505 |
| ERN1 | Endoplasmic Reticulum To Nucleus Signaling 1 | Protein Coding | 45 | GC17M064039 |
| PLAGL1 | PLAG1 Like Zinc Finger 1 | Protein Coding | 43 | GC06M143940 |
| PRIMPOL | Primase And DNA Directed Polymerase | Protein Coding | 33 | GC04P184649 |
| MED25 | Mediator Complex Subunit 25 | Protein Coding | 39 | GC19P049819 |
| LBR | Lamin B Receptor | Protein Coding | 47 | GC01M225401 |
| TJP1 | Tight Junction Protein 1 | Protein Coding | 43 | GC15M029699 |
| GHRH | Growth Hormone Releasing Hormone | Protein Coding | 40 | GC20M037251 |
| CXADR | CXADR Ig-Like Cell Adhesion Molecule | Protein Coding | 43 | GC21P017512 |
| EIF4G1 | Eukaryotic Translation Initiation Factor 4 Gamma 1 | Protein Coding | 45 | GC03P184314 |
| PCDH11X | Protocadherin 11 X-Linked | Protein Coding | 35 | GC0XP091779 |
| GCM2 | Glial Cells Missing Transcription Factor 2 | Protein Coding | 41 | GC06M010873 |
| PLAA | Phospholipase A2 Activating Protein | Protein Coding | 43 | GC09M026903 |
| ATPAF2 | ATP Synthase Mitochondrial F1 Complex Assembly Factor 2 | Protein Coding | 37 | GC17M017977 |
| NSUN6 | NOP2/Sun RNA Methyltransferase 6 | Protein Coding | 36 | GC10M018520 |
| CPOX | Coproporphyrinogen Oxidase | Protein Coding | 43 | GC03M098576 |
| GJA8 | Gap Junction Protein Alpha 8 | Protein Coding | 45 | GC01P147902 |
| SMARCA1 | SWI/SNF Related, Matrix Associated, Actin Dependent Regulator Of Chromatin, Subfamily A, Member 1 | Protein Coding | 39 | GC0XM129447 |
| DNAJC21 | DnaJ Heat Shock Protein Family (Hsp40) Member C21 | Protein Coding | 35 | GC05P034929 |
| TF | Transferrin | Protein Coding | 49 | GC03P133666 |
| NANOG | Nanog Homeobox | Protein Coding | 39 | GC12P007787 |
| SLC16A2 | Solute Carrier Family 16 Member 2 | Protein Coding | 45 | GC0XP074425 |
| IFNA2 | Interferon Alpha 2 | Protein Coding | 41 | GC09M021384 |
| ADM2 | Adrenomedullin 2 | Protein Coding | 33 | GC22P050481 |
| DR1 | Down-Regulator Of Transcription 1 | Protein Coding | 39 | GC01P093345 |
| POU4F1 | POU Class 4 Homeobox 1 | Protein Coding | 38 | GC13M078598 |
| RGS19 | Regulator Of G Protein Signaling 19 | Protein Coding | 40 | GC20M064073 |
| DICER1 | Dicer 1, Ribonuclease III | Protein Coding | 47 | GC14M095086 |
| CCDC65 | Coiled-Coil Domain Containing 65 | Protein Coding | 36 | GC12P048904 |
| TIMM13 | Translocase Of Inner Mitochondrial Membrane 13 | Protein Coding | 36 | GC19M002425 |
| L2HGDH | L-2-Hydroxyglutarate Dehydrogenase | Protein Coding | 40 | GC14M050237 |
| CHRNE | Cholinergic Receptor Nicotinic Epsilon Subunit | Protein Coding | 43 | GC17M004897 |
| BCHE | Butyrylcholinesterase | Protein Coding | 48 | GC03M165772 |
| AKT3 | AKT Serine/Threonine Kinase 3 | Protein Coding | 52 | GC01M243488 |
| DNAAF2 | Dynein Axonemal Assembly Factor 2 | Protein Coding | 37 | GC14M049625 |
| GABBR1 | Gamma-Aminobutyric Acid Type B Receptor Subunit 1 | Protein Coding | 47 | GC06M029555 |
| PPCS | Phosphopantothenoylcysteine Synthetase | Protein Coding | 39 | GC01P042456 |
| MRRF | Mitochondrial Ribosome Recycling Factor | Protein Coding | 39 | GC09P122264 |
| CASP2 | Caspase 2 | Protein Coding | 49 | GC07P144979 |
| WARS2 | Tryptophanyl TRNA Synthetase 2, Mitochondrial | Protein Coding | 44 | GC01M119031 |
| KCNK3 | Potassium Two Pore Domain Channel Subfamily K Member 3 | Protein Coding | 49 | GC02P026692 |
| CYP27B1 | Cytochrome P450 Family 27 Subfamily B Member 1 | Protein Coding | 47 | GC12M057757 |
| RHOA | Ras Homolog Family Member A | Protein Coding | 46 | GC03M049359 |
| PGF | Placental Growth Factor | Protein Coding | 43 | GC14M074941 |
| MIR148A | MicroRNA 148a | RNA Gene | 18 | GC07M025993 |
| ITGAV | Integrin Subunit Alpha V | Protein Coding | 46 | GC02P186589 |
| TRMT5 | TRNA Methyltransferase 5 | Protein Coding | 38 | GC14M060971 |
| TAOK3 | TAO Kinase 3 | Protein Coding | 40 | GC12M118149 |
| DNMT3L | DNA Methyltransferase 3 Like | Protein Coding | 41 | GC21M044246 |
| CHRNA1 | Cholinergic Receptor Nicotinic Alpha 1 Subunit | Protein Coding | 45 | GC02M174747 |
| SALL3 | Spalt Like Transcription Factor 3 | Protein Coding | 36 | GC18P078980 |
| SUN1 | Sad1 And UNC84 Domain Containing 1 | Protein Coding | 37 | GC07P000857 |
| RNASE3 | Ribonuclease A Family Member 3 | Protein Coding | 40 | GC14P020891 |
| PRPH | Peripherin | Protein Coding | 44 | GC12P049293 |
| PRKRA | Protein Activator Of Interferon Induced Protein Kinase EIF2AK2 | Protein Coding | 42 | GC02M178431 |
| UPF1 | UPF1 RNA Helicase And ATPase | Protein Coding | 40 | GC19P018831 |
| ANG | Angiogenin | Protein Coding | 45 | GC14P020830 |
| MX1 | MX Dynamin Like GTPase 1 | Protein Coding | 41 | GC21P041420 |
| RANBP2 | RAN Binding Protein 2 | Protein Coding | 45 | GC02P108719 |
| UHRF1 | Ubiquitin Like With PHD And Ring Finger Domains 1 | Protein Coding | 40 | GC19P004910 |
| DMPK | DM1 Protein Kinase | Protein Coding | 48 | GC19M045769 |
| ISL1 | ISL LIM Homeobox 1 | Protein Coding | 45 | GC05P051383 |
| PGK1 | Phosphoglycerate Kinase 1 | Protein Coding | 48 | GC0XP077928 |
| GLS | Glutaminase | Protein Coding | 46 | GC02P190880 |
| SUCO | SUN Domain Containing Ossification Factor | Protein Coding | 32 | GC01P172532 |
| PYGM | Glycogen Phosphorylase, Muscle Associated | Protein Coding | 46 | GC11M064746 |
| HSPG2 | Heparan Sulfate Proteoglycan 2 | Protein Coding | 45 | GC01M021822 |
| CFAP298 | Cilia And Flagella Associated Protein 298 | Protein Coding | 30 | GC21M032593 |
| RFT1 | RFT1 Homolog | Protein Coding | 38 | GC03M053071 |
| LAMA5 | Laminin Subunit Alpha 5 | Protein Coding | 42 | GC20M062307 |
| NUP133 | Nucleoporin 133 | Protein Coding | 40 | GC01M229441 |
| TMSB15A | Thymosin Beta 15a | Protein Coding | 29 | GC0XM102515 |
| CACNA1G | Calcium Voltage-Gated Channel Subunit Alpha1 G | Protein Coding | 48 | GC17P050561 |
| SLC52A1 | Solute Carrier Family 52 Member 1 | Protein Coding | 38 | GC17M005032 |
| NPS | Neuropeptide S | Protein Coding | 32 | GC10P127549 |
| NTS | Neurotensin | Protein Coding | 40 | GC12P085876 |
| DPM2 | Dolichyl-Phosphate Mannosyltransferase Subunit 2, Regulatory | Protein Coding | 37 | GC09M127935 |
| PYCR2 | Pyrroline-5-Carboxylate Reductase 2 | Protein Coding | 44 | GC01M225919 |
| DRD5 | Dopamine Receptor D5 | Protein Coding | 47 | GC04P009783 |
| ADSL | Adenylosuccinate Lyase | Protein Coding | 47 | GC22P040346 |
| PROS1 | Protein S | Protein Coding | 47 | GC03M093873 |
| RARA | Retinoic Acid Receptor Alpha | Protein Coding | 50 | GC17P040309 |
| RCVRN | Recoverin | Protein Coding | 38 | GC17M009896 |
| SLC9A3 | Solute Carrier Family 9 Member A3 | Protein Coding | 47 | GC05M000472 |
| GGCT | Gamma-Glutamylcyclotransferase | Protein Coding | 39 | GC07M030496 |
| LCOR | Ligand Dependent Nuclear Receptor Corepressor | Protein Coding | 37 | GC10P096832 |
| RASSF1 | Ras Association Domain Family Member 1 | Protein Coding | 44 | GC03M050329 |
| SPTBN4 | Spectrin Beta, Non-Erythrocytic 4 | Protein Coding | 39 | GC19P040466 |
| ITGA6 | Integrin Subunit Alpha 6 | Protein Coding | 49 | GC02P172427 |
| GNPAT | Glyceronephosphate O-Acyltransferase | Protein Coding | 45 | GC01P231241 |
| MARS1 | Methionyl-TRNA Synthetase 1 | Protein Coding | 35 | GC12P057476 |
| FIS1 | Fission, Mitochondrial 1 | Protein Coding | 39 | GC07M101239 |
| BANF1 | BAF Nuclear Assembly Factor 1 | Protein Coding | 42 | GC11P066002 |
| PLIN2 | Perilipin 2 | Protein Coding | 43 | GC09M019115 |
| WHCR | Wolf-Hirschhorn Syndrome Chromosome Region | Genetic Locus | 4 | GC04U990055 |
| CP | Ceruloplasmin | Protein Coding | 47 | GC03M149162 |
| LOC110806262 | Solute Carrier Family 6 Member 4 Gene Promoter | Biological Region | 1 | GC17P030235 |
| LEPR | Leptin Receptor | Protein Coding | 49 | GC01P065421 |
| DMAP1 | DNA Methyltransferase 1 Associated Protein 1 | Protein Coding | 37 | GC01P044214 |
| MFN1 | Mitofusin 1 | Protein Coding | 41 | GC03P179347 |
| HINT1 | Histidine Triad Nucleotide Binding Protein 1 | Protein Coding | 45 | GC05M131159 |
| OGDH | Oxoglutarate Dehydrogenase | Protein Coding | 45 | GC07P044606 |
| PSMD5 | Proteasome 26S Subunit, Non-ATPase 5 | Protein Coding | 38 | GC09M120815 |
| TNFSF13B | TNF Superfamily Member 13b | Protein Coding | 45 | GC13P108251 |
| LEF1 | Lymphoid Enhancer Binding Factor 1 | Protein Coding | 47 | GC04M108047 |
| DAO | D-Amino Acid Oxidase | Protein Coding | 45 | GC12P108859 |
| RNF213 | Ring Finger Protein 213 | Protein Coding | 39 | GC17P080260 |
| IGFBP1 | Insulin Like Growth Factor Binding Protein 1 | Protein Coding | 43 | GC07P046552 |
| MTO1 | Mitochondrial TRNA Translation Optimization 1 | Protein Coding | 42 | GC06P073461 |
| KCTD1 | Potassium Channel Tetramerization Domain Containing 1 | Protein Coding | 40 | GC18M026454 |
| FGF12 | Fibroblast Growth Factor 12 | Protein Coding | 43 | GC03M192139 |
| PDSS2 | Decaprenyl Diphosphate Synthase Subunit 2 | Protein Coding | 40 | GC06M107152 |
| L1CAM | L1 Cell Adhesion Molecule | Protein Coding | 46 | GC0XM153864 |
| MIB2 | Mindbomb E3 Ubiquitin Protein Ligase 2 | Protein Coding | 37 | GC01P001614 |
| ADAMTS2 | ADAM Metallopeptidase With Thrombospondin Type 1 Motif 2 | Protein Coding | 42 | GC05M179110 |
| SLC44A2 | Solute Carrier Family 44 Member 2 | Protein Coding | 40 | GC19P010602 |
| ALDH3A2 | Aldehyde Dehydrogenase 3 Family Member A2 | Protein Coding | 45 | GC17P019648 |
| VIP | Vasoactive Intestinal Peptide | Protein Coding | 44 | GC06P152750 |
| CCN2 | Cellular Communication Network Factor 2 | Protein Coding | 39 | GC06M131948 |
| AR | Androgen Receptor | Protein Coding | 53 | GC0XP067544 |
| PARS2 | Prolyl-TRNA Synthetase 2, Mitochondrial | Protein Coding | 41 | GC01M054756 |
| POC5 | POC5 Centriolar Protein | Protein Coding | 33 | GC05M075674 |
| DDX58 | DExD/H-Box Helicase 58 | Protein Coding | 47 | GC09M032455 |
| BMP6 | Bone Morphogenetic Protein 6 | Protein Coding | 43 | GC06P007726 |
| ERLIN2 | ER Lipid Raft Associated 2 | Protein Coding | 40 | GC08P037736 |
| CD86 | CD86 Molecule | Protein Coding | 43 | GC03P122055 |
| OTOS | Otospiralin | Protein Coding | 29 | GC02M240139 |
| GDF15 | Growth Differentiation Factor 15 | Protein Coding | 41 | GC19P023329 |
| SMARCA5 | SWI/SNF Related, Matrix Associated, Actin Dependent Regulator Of Chromatin, Subfamily A, Member 5 | Protein Coding | 40 | GC04P143513 |
| YY1 | YY1 Transcription Factor | Protein Coding | 48 | GC14P100238 |
| GABRR2 | Gamma-Aminobutyric Acid Type A Receptor Subunit Rho2 | Protein Coding | 40 | GC06M089257 |
| PIGG | Phosphatidylinositol Glycan Anchor Biosynthesis Class G | Protein Coding | 40 | GC04P000486 |
| MMP7 | Matrix Metallopeptidase 7 | Protein Coding | 48 | GC11M102425 |
| CCDC151 | Coiled-Coil Domain Containing 151 | Protein Coding | 37 | GC19M011420 |
| OTX1 | Orthodenticle Homeobox 1 | Protein Coding | 41 | GC02P063050 |
| ALPP | Alkaline Phosphatase, Placental | Protein Coding | 46 | GC02P232378 |
| OLIG2 | Oligodendrocyte Transcription Factor 2 | Protein Coding | 40 | GC21P033025 |
| NCOR1 | Nuclear Receptor Corepressor 1 | Protein Coding | 43 | GC17M016029 |
| FAR1 | Fatty Acyl-CoA Reductase 1 | Protein Coding | 43 | GC11P013668 |
| NGLY1 | N-Glycanase 1 | Protein Coding | 44 | GC03M025718 |
| SLC32A1 | Solute Carrier Family 32 Member 1 | Protein Coding | 42 | GC20P038724 |
| SPTSSA | Serine Palmitoyltransferase Small Subunit A | Protein Coding | 34 | GC14M034432 |
| CARD14 | Caspase Recruitment Domain Family Member 14 | Protein Coding | 43 | GC17P080170 |
| CENPI | Centromere Protein I | Protein Coding | 35 | GC0XP101098 |
| CELSR3 | Cadherin EGF LAG Seven-Pass G-Type Receptor 3 | Protein Coding | 39 | GC03M048641 |
| MIR16-1 | MicroRNA 16-1 | RNA Gene | 21 | GC13M050048 |
| AUH | AU RNA Binding Methylglutaconyl-CoA Hydratase | Protein Coding | 43 | GC09M091213 |
| HECTD4 | HECT Domain E3 Ubiquitin Protein Ligase 4 | Protein Coding | 31 | GC12M112160 |
| AMPH | Amphiphysin | Protein Coding | 44 | GC07M038666 |
| CLCN6 | Chloride Voltage-Gated Channel 6 | Protein Coding | 41 | GC01P011806 |
| PROCR | Protein C Receptor | Protein Coding | 42 | GC20P035182 |
| GRIK1 | Glutamate Ionotropic Receptor Kainate Type Subunit 1 | Protein Coding | 45 | GC21M029536 |
| CCDC78 | Coiled-Coil Domain Containing 78 | Protein Coding | 36 | GC16M001132 |
| NEAT1 | Nuclear Paraspeckle Assembly Transcript 1 | RNA Gene | 23 | GC11P065794 |
| SUN2 | Sad1 And UNC84 Domain Containing 2 | Protein Coding | 37 | GC22M045390 |
| MYBPC1 | Myosin Binding Protein C1 | Protein Coding | 43 | GC12P101568 |
| IL15 | Interleukin 15 | Protein Coding | 40 | GC04P141636 |
| BAMBI | BMP And Activin Membrane Bound Inhibitor | Protein Coding | 43 | GC10P028685 |
| MIR335 | MicroRNA 335 | RNA Gene | 18 | GC07P130496 |
| ZNHIT3 | Zinc Finger HIT-Type Containing 3 | Protein Coding | 36 | GC17P036486 |
| KAT8 | Lysine Acetyltransferase 8 | Protein Coding | 40 | GC16P031537 |
| CD38 | CD38 Molecule | Protein Coding | 45 | GC04P015779 |
| CHIT1 | Chitinase 1 | Protein Coding | 43 | GC01M203181 |
| BRD4 | Bromodomain Containing 4 | Protein Coding | 44 | GC19M015236 |
| CXCL13 | C-X-C Motif Chemokine Ligand 13 | Protein Coding | 41 | GC04P077511 |
| UCN | Urocortin | Protein Coding | 37 | GC02M027308 |
| PEX11A | Peroxisomal Biogenesis Factor 11 Alpha | Protein Coding | 37 | GC15M089677 |
| ADAM17 | ADAM Metallopeptidase Domain 17 | Protein Coding | 51 | GC02M009488 |
| AIM2 | Absent In Melanoma 2 | Protein Coding | 41 | GC01M159062 |
| DYNLL1 | Dynein Light Chain LC8-Type 1 | Protein Coding | 42 | GC12P120469 |
| ABCG2 | ATP Binding Cassette Subfamily G Member 2 (Junior Blood Group) | Protein Coding | 50 | GC04M088090 |
| DEFB1 | Defensin Beta 1 | Protein Coding | 39 | GC08M006870 |
| NEDD4 | NEDD4 E3 Ubiquitin Protein Ligase | Protein Coding | 45 | GC15M055826 |
| CDK6 | Cyclin Dependent Kinase 6 | Protein Coding | 54 | GC07M092604 |
| MAP2K7 | Mitogen-Activated Protein Kinase Kinase 7 | Protein Coding | 45 | GC19P007903 |
| NNT | Nicotinamide Nucleotide Transhydrogenase | Protein Coding | 45 | GC05P043603 |
| NRP1 | Neuropilin 1 | Protein Coding | 47 | GC10M033177 |
| FTH1 | Ferritin Heavy Chain 1 | Protein Coding | 50 | GC11M061959 |
| SERPIND1 | Serpin Family D Member 1 | Protein Coding | 45 | GC22P020836 |
| YWHAQ | Tyrosine 3-Monooxygenase/Tryptophan 5-Monooxygenase Activation Protein Theta | Protein Coding | 47 | GC02M009583 |
| SOCS1 | Suppressor Of Cytokine Signaling 1 | Protein Coding | 43 | GC16M011255 |
| YWHAE | Tyrosine 3-Monooxygenase/Tryptophan 5-Monooxygenase Activation Protein Epsilon | Protein Coding | 50 | GC17M001346 |
| CPS1 | Carbamoyl-Phosphate Synthase 1 | Protein Coding | 45 | GC02P210477 |
| IRF3 | Interferon Regulatory Factor 3 | Protein Coding | 47 | GC19M049659 |
| POU1F1 | POU Class 1 Homeobox 1 | Protein Coding | 43 | GC03M087259 |
| SYK | Spleen Associated Tyrosine Kinase | Protein Coding | 50 | GC09P091171 |
| RPA1 | Replication Protein A1 | Protein Coding | 45 | GC17P001829 |
| PRKCH | Protein Kinase C Eta | Protein Coding | 50 | GC14P061187 |
| MTM1 | Myotubularin 1 | Protein Coding | 44 | GC0XP150562 |
| ACAA1 | Acetyl-CoA Acyltransferase 1 | Protein Coding | 43 | GC03M038103 |
| PREPL | Prolyl Endopeptidase Like | Protein Coding | 41 | GC02M044281 |
| MBD2 | Methyl-CpG Binding Domain Protein 2 | Protein Coding | 40 | GC18M054151 |
| PARP1 | Poly(ADP-Ribose) Polymerase 1 | Protein Coding | 49 | GC01M226360 |
| GAL | Galanin And GMAP Prepropeptide | Protein Coding | 44 | GC11P068684 |
| SLC11A2 | Solute Carrier Family 11 Member 2 | Protein Coding | 47 | GC12M050952 |
| PDLIM1 | PDZ And LIM Domain 1 | Protein Coding | 40 | GC10M095237 |
| TCF21 | Transcription Factor 21 | Protein Coding | 36 | GC06P133889 |
| KL | Klotho | Protein Coding | 44 | GC13P033016 |
| TNFRSF10C | TNF Receptor Superfamily Member 10c | Protein Coding | 39 | GC08P023102 |
| IL2RG | Interleukin 2 Receptor Subunit Gamma | Protein Coding | 48 | GC0XM071108 |
| SNCG | Synuclein Gamma | Protein Coding | 42 | GC10P086957 |
| TLR8 | Toll Like Receptor 8 | Protein Coding | 47 | GC0XP012924 |
| CXCL9 | C-X-C Motif Chemokine Ligand 9 | Protein Coding | 39 | GC04M076001 |
| ATP12A | ATPase H+/K+ Transporting Non-Gastric Alpha2 Subunit | Protein Coding | 43 | GC13P024680 |
| MTERF1 | Mitochondrial Transcription Termination Factor 1 | Protein Coding | 34 | GC07M091692 |
| CLOCK | Clock Circadian Regulator | Protein Coding | 43 | GC04M055427 |
| KCNH5 | Potassium Voltage-Gated Channel Subfamily H Member 5 | Protein Coding | 43 | GC14M062699 |
| CHCHD2 | Coiled-Coil-Helix-Coiled-Coil-Helix Domain Containing 2 | Protein Coding | 39 | GC07M056101 |
| P4HB | Prolyl 4-Hydroxylase Subunit Beta | Protein Coding | 49 | GC17M081843 |
| RASA1 | RAS P21 Protein Activator 1 | Protein Coding | 46 | GC05P087267 |
| ATP4A | ATPase H+/K+ Transporting Subunit Alpha | Protein Coding | 41 | GC19M042544 |
| SLC5A6 | Solute Carrier Family 5 Member 6 | Protein Coding | 44 | GC02M027201 |
| GFPT1 | Glutamine--Fructose-6-Phosphate Transaminase 1 | Protein Coding | 46 | GC02M069283 |
| CHKA | Choline Kinase Alpha | Protein Coding | 41 | GC11M068052 |
| ATP1B1 | ATPase Na+/K+ Transporting Subunit Beta 1 | Protein Coding | 47 | GC01P169105 |
| VPS13C | Vacuolar Protein Sorting 13 Homolog C | Protein Coding | 35 | GC15M061852 |
| NR4A2 | Nuclear Receptor Subfamily 4 Group A Member 2 | Protein Coding | 46 | GC02M156324 |
| TLR7 | Toll Like Receptor 7 | Protein Coding | 46 | GC0XP012867 |
| UMOD | Uromodulin | Protein Coding | 41 | GC16M020344 |
| CHGB | Chromogranin B | Protein Coding | 39 | GC20P005911 |
| GSTT1 | Glutathione S-Transferase Theta 1 | Protein Coding | 32 | GC22Mi00270 |
| CNBP | CCHC-Type Zinc Finger Nucleic Acid Binding Protein | Protein Coding | 41 | GC03M129167 |
| SPTSSB | Serine Palmitoyltransferase Small Subunit B | Protein Coding | 32 | GC03M161344 |
| HSPA1A | Heat Shock Protein Family A (Hsp70) Member 1A | Protein Coding | 43 | GC06P047326 |
| TNNI2 | Troponin I2, Fast Skeletal Type | Protein Coding | 44 | GC11P001839 |
| RCC1L | RCC1 Like | Protein Coding | 24 | GC07M075029 |
| EZR | Ezrin | Protein Coding | 45 | GC06M158765 |
| GOLGA2 | Golgin A2 | Protein Coding | 40 | GC09M128255 |
| DLL3 | Delta Like Canonical Notch Ligand 3 | Protein Coding | 41 | GC19P039498 |
| ZBTB24 | Zinc Finger And BTB Domain Containing 24 | Protein Coding | 38 | GC06M109462 |
| CERS1 | Ceramide Synthase 1 | Protein Coding | 41 | GC19M018868 |
| OMA1 | OMA1 Zinc Metallopeptidase | Protein Coding | 37 | GC01M058415 |
| STK36 | Serine/Threonine Kinase 36 | Protein Coding | 43 | GC02P218672 |
| VCAM1 | Vascular Cell Adhesion Molecule 1 | Protein Coding | 45 | GC01P100719 |
| MMD | Monocyte To Macrophage Differentiation Associated | Protein Coding | 35 | GC17M055392 |
| MESP2 | Mesoderm Posterior BHLH Transcription Factor 2 | Protein Coding | 35 | GC15P089764 |
| GRK7 | G Protein-Coupled Receptor Kinase 7 | Protein Coding | 39 | GC03P141778 |
| NLRP6 | NLR Family Pyrin Domain Containing 6 | Protein Coding | 38 | GC11P000269 |
| PFN1 | Profilin 1 | Protein Coding | 47 | GC17M004945 |
| LMO7 | LIM Domain 7 | Protein Coding | 40 | GC13P075620 |
| CTSB | Cathepsin B | Protein Coding | 51 | GC08M011842 |
| AKAP1 | A-Kinase Anchoring Protein 1 | Protein Coding | 39 | GC17P057085 |
| CASQ1 | Calsequestrin 1 | Protein Coding | 43 | GC01P160190 |
| MIR23B | MicroRNA 23b | RNA Gene | 20 | GC09P095085 |
| RND2 | Rho Family GTPase 2 | Protein Coding | 36 | GC17P043995 |
| SLC27A5 | Solute Carrier Family 27 Member 5 | Protein Coding | 43 | GC19M058479 |
| MGMT | O-6-Methylguanine-DNA Methyltransferase | Protein Coding | 50 | GC10P129467 |
| JAG2 | Jagged Canonical Notch Ligand 2 | Protein Coding | 42 | GC14M105140 |
| VARS2 | Valyl-TRNA Synthetase 2, Mitochondrial | Protein Coding | 43 | GC06P047290 |
| COQ8B | Coenzyme Q8B | Protein Coding | 32 | GC19M042476 |
| GORASP1 | Golgi Reassembly Stacking Protein 1 | Protein Coding | 40 | GC03M039096 |
| SLC12A1 | Solute Carrier Family 12 Member 1 | Protein Coding | 46 | GC15P048191 |
| LPIN2 | Lipin 2 | Protein Coding | 42 | GC18M002906 |
| MFF | Mitochondrial Fission Factor | Protein Coding | 37 | GC02P227325 |
| MSN | Moesin | Protein Coding | 47 | GC0XP065588 |
| B3GAT3 | Beta-1,3-Glucuronyltransferase 3 | Protein Coding | 45 | GC11M063429 |
| PHB | Prohibitin | Protein Coding | 47 | GC17M049404 |
| GGT2 | Gamma-Glutamyltransferase 2 | Protein Coding | 31 | GC22M021207 |
| TRMT61B | TRNA Methyltransferase 61B | Protein Coding | 33 | GC02M028814 |
| CKAP5 | Cytoskeleton Associated Protein 5 | Protein Coding | 40 | GC11M061109 |
| PLAUR | Plasminogen Activator, Urokinase Receptor | Protein Coding | 44 | GC19M043646 |
| PTHLH | Parathyroid Hormone Like Hormone | Protein Coding | 45 | GC12M027959 |
| CYP4F22 | Cytochrome P450 Family 4 Subfamily F Member 22 | Protein Coding | 39 | GC19P015508 |
| UCP2 | Uncoupling Protein 2 | Protein Coding | 45 | GC11M073974 |
| ATXN10 | Ataxin 10 | Protein Coding | 42 | GC22P045673 |
| TRMT11 | TRNA Methyltransferase 11 Homolog | Protein Coding | 37 | GC06P125986 |
| TRMT112 | TRNA Methyltransferase Subunit 11-2 | Protein Coding | 36 | GC11M064316 |
| NTRK3 | Neurotrophic Receptor Tyrosine Kinase 3 | Protein Coding | 51 | GC15M087859 |
| FKBP1A | FKBP Prolyl Isomerase 1A | Protein Coding | 46 | GC20M001369 |
| TLDC2 | TBC/LysM-Associated Domain Containing 2 | Protein Coding | 28 | GC20P036876 |
| PPL | Periplakin | Protein Coding | 40 | GC16M004872 |
| DMRTA1 | DMRT Like Family A1 | Protein Coding | 36 | GC09P022436 |
| IL1RAPL2 | Interleukin 1 Receptor Accessory Protein Like 2 | Protein Coding | 37 | GC0XP104566 |
| LOC107988032 | Xq28 Proximal FLNA-EMD Recombination Region | Biological Region | 1 | GC0XP154335 |
| TARID | TCF21 Antisense RNA Inducing Promoter Demethylation | RNA Gene | 15 | GC06M133502 |
| GIGYF2 | GRB10 Interacting GYF Protein 2 | Protein Coding | 39 | GC02P232698 |
| ZIC3 | Zic Family Member 3 | Protein Coding | 46 | GC0XP137566 |
| RICTOR | RPTOR Independent Companion Of MTOR Complex 2 | Protein Coding | 44 | GC05M038939 |
| MNX1 | Motor Neuron And Pancreas Homeobox 1 | Protein Coding | 41 | GC07M156994 |
| PNMA2 | PNMA Family Member 2 | Protein Coding | 36 | GC08M026504 |
| CASP5 | Caspase 5 | Protein Coding | 44 | GC11M104995 |
| RARB | Retinoic Acid Receptor Beta | Protein Coding | 50 | GC03P024830 |
| RBPJ | Recombination Signal Binding Protein For Immunoglobulin Kappa J Region | Protein Coding | 47 | GC04P026165 |
| IFNAR1 | Interferon Alpha And Beta Receptor Subunit 1 | Protein Coding | 45 | GC21P033324 |
| LUZP1 | Leucine Zipper Protein 1 | Protein Coding | 34 | GC01M023085 |
| PUS1 | Pseudouridine Synthase 1 | Protein Coding | 41 | GC12P131929 |
| AMPD1 | Adenosine Monophosphate Deaminase 1 | Protein Coding | 45 | GC01M114673 |
| MRGPRG-AS1 | MRGPRG Antisense RNA 1 | RNA Gene | 20 | GC11P003218 |
| PTGDS | Prostaglandin D2 Synthase | Protein Coding | 45 | GC09P136982 |
| AQP3 | Aquaporin 3 (Gill Blood Group) | Protein Coding | 47 | GC09M033431 |
| CDKN2C | Cyclin Dependent Kinase Inhibitor 2C | Protein Coding | 45 | GC01P050960 |
| PTGFR | Prostaglandin F Receptor | Protein Coding | 44 | GC01P078303 |
| SSTR2 | Somatostatin Receptor 2 | Protein Coding | 47 | GC17P073165 |
| TSG101 | Tumor Susceptibility 101 | Protein Coding | 43 | GC11M018468 |
| ASL | Argininosuccinate Lyase | Protein Coding | 45 | GC07P066075 |
| HP | Haptoglobin | Protein Coding | 44 | GC16P072089 |
| SHKBP1 | SH3KBP1 Binding Protein 1 | Protein Coding | 36 | GC19P040576 |
| GGTLC3 | Gamma-Glutamyltransferase Light Chain Family Member 3 | Protein Coding | 20 | GC22M018516 |
| LPIN1 | Lipin 1 | Protein Coding | 48 | GC02P011649 |
| RHOT1 | Ras Homolog Family Member T1 | Protein Coding | 43 | GC17P032142 |
| MIEF1 | Mitochondrial Elongation Factor 1 | Protein Coding | 32 | GC22P039501 |
| PRKCA | Protein Kinase C Alpha | Protein Coding | 50 | GC17P066302 |
| ETS1 | ETS Proto-Oncogene 1, Transcription Factor | Protein Coding | 49 | GC11M128458 |
| DPP4 | Dipeptidyl Peptidase 4 | Protein Coding | 50 | GC02M161992 |
| MIEF2 | Mitochondrial Elongation Factor 2 | Protein Coding | 33 | GC17P018262 |
| AQP6 | Aquaporin 6 | Protein Coding | 37 | GC12P049967 |
| ADRA1A | Adrenoceptor Alpha 1A | Protein Coding | 47 | GC08M026747 |
| CSGALNACT1 | Chondroitin Sulfate N-Acetylgalactosaminyltransferase 1 | Protein Coding | 40 | GC08M019404 |
| NCS1 | Neuronal Calcium Sensor 1 | Protein Coding | 41 | GC09P130172 |
| NUP153 | Nucleoporin 153 | Protein Coding | 40 | GC06M017615 |
| GPD1 | Glycerol-3-Phosphate Dehydrogenase 1 | Protein Coding | 45 | GC12P050105 |
| PGR | Progesterone Receptor | Protein Coding | 50 | GC11M100943 |
| EMC1 | ER Membrane Protein Complex Subunit 1 | Protein Coding | 35 | GC01M019215 |
| FGF1 | Fibroblast Growth Factor 1 | Protein Coding | 48 | GC05M142555 |
| PPIF | Peptidylprolyl Isomerase F | Protein Coding | 43 | GC10P083661 |
| IMMP2L | Inner Mitochondrial Membrane Peptidase Subunit 2 | Protein Coding | 39 | GC07M110663 |
| DNA2 | DNA Replication Helicase/Nuclease 2 | Protein Coding | 41 | GC10M068414 |
| CDH10 | Cadherin 10 | Protein Coding | 39 | GC05M024522 |
| PRKCE | Protein Kinase C Epsilon | Protein Coding | 50 | GC02P045651 |
| SLC26A1 | Solute Carrier Family 26 Member 1 | Protein Coding | 40 | GC04M000979 |
| PLEK | Pleckstrin | Protein Coding | 40 | GC02P068365 |
| ROS1 | ROS Proto-Oncogene 1, Receptor Tyrosine Kinase | Protein Coding | 45 | GC06M117287 |
| NSUN3 | NOP2/Sun RNA Methyltransferase 3 | Protein Coding | 36 | GC03P094062 |
| CUTC | CutC Copper Transporter | Protein Coding | 37 | GC10P099702 |
| NLGN4X | Neuroligin 4 X-Linked | Protein Coding | 40 | GC0XM005840 |
| STH | Saitohin | Protein Coding | 30 | GC17P045999 |
| AKAP13 | A-Kinase Anchoring Protein 13 | Protein Coding | 44 | GC15P085381 |
| RTN2 | Reticulon 2 | Protein Coding | 40 | GC19M045485 |
| ADCY3 | Adenylate Cyclase 3 | Protein Coding | 47 | GC02M024819 |
| ID1 | Inhibitor Of DNA Binding 1, HLH Protein | Protein Coding | 43 | GC20P031605 |
| LEXM | Lymphocyte Expansion Molecule | Protein Coding | 24 | GC01P054807 |
| NFATC1 | Nuclear Factor Of Activated T Cells 1 | Protein Coding | 47 | GC18P079395 |
| DLG4 | Discs Large MAGUK Scaffold Protein 4 | Protein Coding | 47 | GC17M007189 |
| TRIT1 | TRNA Isopentenyltransferase 1 | Protein Coding | 41 | GC01M039842 |
| AUTS2 | Activator Of Transcription And Developmental Regulator AUTS2 | Protein Coding | 39 | GC07P069598 |
| POTEF | POTE Ankyrin Domain Family Member F | Protein Coding | 28 | GC02M130073 |
| MRPS6 | Mitochondrial Ribosomal Protein S6 | Protein Coding | 37 | GC21P034110 |
| AQP7 | Aquaporin 7 | Protein Coding | 44 | GC09M033384 |
| TRPM8 | Transient Receptor Potential Cation Channel Subfamily M Member 8 | Protein Coding | 43 | GC02P233917 |
| SRSF1 | Serine And Arginine Rich Splicing Factor 1 | Protein Coding | 41 | GC17M058000 |
| TCF3 | Transcription Factor 3 | Protein Coding | 45 | GC19M001609 |
| TFB2M | Transcription Factor B2, Mitochondrial | Protein Coding | 37 | GC01M246540 |
| SLC25A3 | Solute Carrier Family 25 Member 3 | Protein Coding | 45 | GC12P098593 |
| MNT | MAX Network Transcriptional Repressor | Protein Coding | 36 | GC17M002384 |
| UBC | Ubiquitin C | Protein Coding | 43 | GC12M124911 |
| COA6 | Cytochrome C Oxidase Assembly Factor 6 | Protein Coding | 36 | GC01P234374 |
| LBX1 | Ladybird Homeobox 1 | Protein Coding | 37 | GC10M101226 |
| ALG6 | ALG6 Alpha-1,3-Glucosyltransferase | Protein Coding | 41 | GC01P063367 |
| COLQ | Collagen Like Tail Subunit Of Asymmetric Acetylcholinesterase | Protein Coding | 39 | GC03M015815 |
| IFT81 | Intraflagellar Transport 81 | Protein Coding | 39 | GC12P110124 |
| REL | REL Proto-Oncogene, NF-KB Subunit | Protein Coding | 46 | GC02P060881 |
| USP6 | Ubiquitin Specific Peptidase 6 | Protein Coding | 40 | GC17P005116 |
| RHOV | Ras Homolog Family Member V | Protein Coding | 33 | GC15M040872 |
| CAMP | Cathelicidin Antimicrobial Peptide | Protein Coding | 41 | GC03P048266 |
| MYO1H | Myosin IH | Protein Coding | 35 | GC12P109347 |
| ALG14 | ALG14 UDP-N-Acetylglucosaminyltransferase Subunit | Protein Coding | 41 | GC01M094974 |
| LFNG | LFNG O-Fucosylpeptide 3-Beta-N-Acetylglucosaminyltransferase | Protein Coding | 46 | GC07P002512 |
| STAG3 | Stromal Antigen 3 | Protein Coding | 41 | GC07P100177 |
| TRPV1 | Transient Receptor Potential Cation Channel Subfamily V Member 1 | Protein Coding | 46 | GC17M003565 |
| FOXP1 | Forkhead Box P1 | Protein Coding | 45 | GC03M070926 |
| GNE | Glucosamine (UDP-N-Acetyl)-2-Epimerase/N-Acetylmannosamine Kinase | Protein Coding | 43 | GC09M036214 |
| SPTBN1 | Spectrin Beta, Non-Erythrocytic 1 | Protein Coding | 43 | GC02P054456 |
| CCDC114 | Coiled-Coil Domain Containing 114 | Protein Coding | 36 | GC19M048296 |
| IVL | Involucrin | Protein Coding | 37 | GC01P152881 |
| ERBB3 | Erb-B2 Receptor Tyrosine Kinase 3 | Protein Coding | 54 | GC12P056094 |
| RORB | RAR Related Orphan Receptor B | Protein Coding | 46 | GC09P074497 |
| IRF4 | Interferon Regulatory Factor 4 | Protein Coding | 43 | GC06P000391 |
| SYN1 | Synapsin I | Protein Coding | 44 | GC0XM047571 |
| YWHAB | Tyrosine 3-Monooxygenase/Tryptophan 5-Monooxygenase Activation Protein Beta | Protein Coding | 48 | GC20P044885 |
| SLC10A7 | Solute Carrier Family 10 Member 7 | Protein Coding | 38 | GC04M146253 |
| GABARAP | GABA Type A Receptor-Associated Protein | Protein Coding | 44 | GC17M007240 |
| ORM1 | Orosomucoid 1 | Protein Coding | 39 | GC09P114323 |
| G6PC3 | Glucose-6-Phosphatase Catalytic Subunit 3 | Protein Coding | 41 | GC17P044070 |
| MAN1B1 | Mannosidase Alpha Class 1B Member 1 | Protein Coding | 46 | GC09P137086 |
| SIM1 | SIM BHLH Transcription Factor 1 | Protein Coding | 40 | GC06M100386 |
| LYZ | Lysozyme | Protein Coding | 47 | GC12P069348 |
| PHB2 | Prohibitin 2 | Protein Coding | 40 | GC12M006965 |
| KDM1A | Lysine Demethylase 1A | Protein Coding | 47 | GC01P023019 |
| APOC3 | Apolipoprotein C3 | Protein Coding | 43 | GC11P116829 |
| FLT1 | Fms Related Receptor Tyrosine Kinase 1 | Protein Coding | 51 | GC13M028300 |
| FZD9 | Frizzled Class Receptor 9 | Protein Coding | 44 | GC07P073433 |
| HRH4 | Histamine Receptor H4 | Protein Coding | 45 | GC18P024460 |
| ATP11A | ATPase Phospholipid Transporting 11A | Protein Coding | 41 | GC13P112690 |
| PDLIM3 | PDZ And LIM Domain 3 | Protein Coding | 37 | GC04M185500 |
| LMO1 | LIM Domain Only 1 | Protein Coding | 43 | GC11M008224 |
| RHOB | Ras Homolog Family Member B | Protein Coding | 44 | GC02P020447 |
| KIF21A | Kinesin Family Member 21A | Protein Coding | 39 | GC12M039293 |
| DEFB4A | Defensin Beta 4A | Protein Coding | 37 | GC08P007895 |
| XPO1 | Exportin 1 | Protein Coding | 45 | GC02M061445 |
| PPIA | Peptidylprolyl Isomerase A | Protein Coding | 47 | GC07P044807 |
| EDA | Ectodysplasin A | Protein Coding | 41 | GC0XP069618 |
| GJA4 | Gap Junction Protein Alpha 4 | Protein Coding | 43 | GC01P034792 |
| STC1 | Stanniocalcin 1 | Protein Coding | 40 | GC08M023841 |
| GGCX | Gamma-Glutamyl Carboxylase | Protein Coding | 46 | GC02M085544 |
| ACO1 | Aconitase 1 | Protein Coding | 44 | GC09P032374 |
| STUB1 | STIP1 Homology And U-Box Containing Protein 1 | Protein Coding | 45 | GC16P001405 |
| UPK3A | Uroplakin 3A | Protein Coding | 37 | GC22P045284 |
| IGHE | Immunoglobulin Heavy Constant Epsilon | Protein Coding | 26 | GC14M109515 |
| CFL1 | Cofilin 1 | Protein Coding | 45 | GC11M065823 |
| CRABP1 | Cellular Retinoic Acid Binding Protein 1 | Protein Coding | 41 | GC15P078340 |
| TECPR2 | Tectonin Beta-Propeller Repeat Containing 2 | Protein Coding | 35 | GC14P102362 |
| ATRIP | ATR Interacting Protein | Protein Coding | 41 | GC03P048449 |
| GRIN2C | Glutamate Ionotropic Receptor NMDA Type Subunit 2C | Protein Coding | 44 | GC17M074842 |
| SYNE3 | Spectrin Repeat Containing Nuclear Envelope Family Member 3 | Protein Coding | 34 | GC14M095408 |
| CLDN4 | Claudin 4 | Protein Coding | 41 | GC07P073799 |
| SPAST | Spastin | Protein Coding | 40 | GC02P032063 |
| CD22 | CD22 Molecule | Protein Coding | 45 | GC19P035319 |
| DGKE | Diacylglycerol Kinase Epsilon | Protein Coding | 48 | GC17P056834 |
| SLC35A3 | Solute Carrier Family 35 Member A3 | Protein Coding | 42 | GC01P099968 |
| XRCC3 | X-Ray Repair Cross Complementing 3 | Protein Coding | 41 | GC14M103697 |
| LAX1 | Lymphocyte Transmembrane Adaptor 1 | Protein Coding | 36 | GC01P203768 |
| GFRA1 | GDNF Family Receptor Alpha 1 | Protein Coding | 44 | GC10M116056 |
| HES7 | Hes Family BHLH Transcription Factor 7 | Protein Coding | 37 | GC17M008120 |
| DDC | Dopa Decarboxylase | Protein Coding | 52 | GC07M050458 |
| DENR | Density Regulated Re-Initiation And Release Factor | Protein Coding | 37 | GC12P122752 |
| FOXM1 | Forkhead Box M1 | Protein Coding | 44 | GC12M002857 |
| GNAS-AS1 | GNAS Antisense RNA 1 | RNA Gene | 22 | GC20M058846 |
| TNXB | Tenascin XB | Protein Coding | 43 | GC06M032635 |
| ATF4 | Activating Transcription Factor 4 | Protein Coding | 46 | GC22P039525 |
| HACL1 | 2-Hydroxyacyl-CoA Lyase 1 | Protein Coding | 40 | GC03M015823 |
| RAC3 | Rac Family Small GTPase 3 | Protein Coding | 45 | GC17P082031 |
| THBS1 | Thrombospondin 1 | Protein Coding | 44 | GC15P039581 |
| TRPC3 | Transient Receptor Potential Cation Channel Subfamily C Member 3 | Protein Coding | 48 | GC04M121879 |
| POU3F2 | POU Class 3 Homeobox 2 | Protein Coding | 43 | GC06P098834 |
| GPX1 | Glutathione Peroxidase 1 | Protein Coding | 48 | GC03M049368 |
| NDUFAF7 | NADH:Ubiquinone Oxidoreductase Complex Assembly Factor 7 | Protein Coding | 34 | GC02P037231 |
| FGF21 | Fibroblast Growth Factor 21 | Protein Coding | 40 | GC19P048766 |
| TMLHE | Trimethyllysine Hydroxylase, Epsilon | Protein Coding | 43 | GC0XM155489 |
| MAVS | Mitochondrial Antiviral Signaling Protein | Protein Coding | 40 | GC20P003827 |
| SLC6A8 | Solute Carrier Family 6 Member 8 | Protein Coding | 45 | GC0XP153688 |
| COX6C | Cytochrome C Oxidase Subunit 6C | Protein Coding | 39 | GC08M099899 |
| RELN | Reelin | Protein Coding | 43 | GC07M103471 |
| TNFRSF25 | TNF Receptor Superfamily Member 25 | Protein Coding | 43 | GC01M006460 |
| HSPE1 | Heat Shock Protein Family E (Hsp10) Member 1 | Protein Coding | 40 | GC02P197501 |
| NOP2 | NOP2 Nucleolar Protein | Protein Coding | 36 | GC12M006556 |
| VILL | Villin Like | Protein Coding | 35 | GC03P037989 |
| HMOX2 | Heme Oxygenase 2 | Protein Coding | 47 | GC16P004474 |
| YWHAZ | Tyrosine 3-Monooxygenase/Tryptophan 5-Monooxygenase Activation Protein Zeta | Protein Coding | 48 | GC08M100917 |
| SPI1 | Spi-1 Proto-Oncogene | Protein Coding | 44 | GC11M061125 |
| SDCBP | Syndecan Binding Protein | Protein Coding | 41 | GC08P058539 |
| FAM3D | FAM3 Metabolism Regulating Signaling Molecule D | Protein Coding | 37 | GC03M058633 |
| FECH | Ferrochelatase | Protein Coding | 45 | GC18M057544 |
| MARCHF5 | Membrane Associated Ring-CH-Type Finger 5 | Protein Coding | 31 | GC10P092292 |
| BIRC5 | Baculoviral IAP Repeat Containing 5 | Protein Coding | 47 | GC17P078214 |
| POU5F1 | POU Class 5 Homeobox 1 | Protein Coding | 47 | GC06M031184 |
| PLA2G4A | Phospholipase A2 Group IVA | Protein Coding | 49 | GC01P186798 |
| THRB | Thyroid Hormone Receptor Beta | Protein Coding | 50 | GC03M024117 |
| HIP1 | Huntingtin Interacting Protein 1 | Protein Coding | 41 | GC07M075533 |
| GUK1 | Guanylate Kinase 1 | Protein Coding | 42 | GC01P228139 |
| IER3 | Immediate Early Response 3 | Protein Coding | 38 | GC06M030743 |
| RCE1 | Ras Converting CAAX Endopeptidase 1 | Protein Coding | 38 | GC11P066842 |
| GML | Glycosylphosphatidylinositol Anchored Molecule Like | Protein Coding | 32 | GC08P142834 |
| BUD23 | BUD23 RRNA Methyltransferase And Ribosome Maturation Factor | Protein Coding | 30 | GC07P073685 |
| LIN9 | Lin-9 DREAM MuvB Core Complex Component | Protein Coding | 37 | GC01M226231 |
| H1-4 | H1.4 Linker Histone, Cluster Member | Protein Coding | 33 | GC06P028871 |
| ID3 | Inhibitor Of DNA Binding 3, HLH Protein | Protein Coding | 41 | GC01M023557 |
| POLRMT | RNA Polymerase Mitochondrial | Protein Coding | 41 | GC19M000617 |
| UHRF2 | Ubiquitin Like With PHD And Ring Finger Domains 2 | Protein Coding | 39 | GC09P006405 |
| HBS1L | HBS1 Like Translational GTPase | Protein Coding | 39 | GC06M134960 |
| CRYGC | Crystallin Gamma C | Protein Coding | 40 | GC02M208128 |
| HEYL | Hes Related Family BHLH Transcription Factor With YRPW Motif Like | Protein Coding | 39 | GC01M039623 |
| SUGCT | Succinyl-CoA:Glutarate-CoA Transferase | Protein Coding | 36 | GC07P040134 |
| CLDN3 | Claudin 3 | Protein Coding | 40 | GC07M073768 |
| MRM2 | Mitochondrial RRNA Methyltransferase 2 | Protein Coding | 31 | GC07M002234 |
| CDH4 | Cadherin 4 | Protein Coding | 41 | GC20P061252 |
| UBR5 | Ubiquitin Protein Ligase E3 Component N-Recognin 5 | Protein Coding | 42 | GC08M102252 |
| LYRM4 | LYR Motif Containing 4 | Protein Coding | 38 | GC06M005032 |
| ADI1 | Acireductone Dioxygenase 1 | Protein Coding | 41 | GC02M003501 |
| SELENON | Selenoprotein N | Protein Coding | 32 | GC01P025800 |
| PTTG1 | PTTG1 Regulator Of Sister Chromatid Separation, Securin | Protein Coding | 43 | GC05P160422 |
| CRYBA1 | Crystallin Beta A1 | Protein Coding | 40 | GC17P029246 |
| CRYGS | Crystallin Gamma S | Protein Coding | 40 | GC03M186538 |
| ABCB11 | ATP Binding Cassette Subfamily B Member 11 | Protein Coding | 45 | GC02M168922 |
| MYRF | Myelin Regulatory Factor | Protein Coding | 34 | GC11P061753 |
| TPMT | Thiopurine S-Methyltransferase | Protein Coding | 47 | GC06M018128 |
| TARM1 | T Cell-Interacting, Activating Receptor On Myeloid Cells 1 | Protein Coding | 31 | GC19M054069 |
| RBBP8 | RB Binding Protein 8, Endonuclease | Protein Coding | 44 | GC18P022798 |
| CA1 | Carbonic Anhydrase 1 | Protein Coding | 47 | GC08M085327 |
| PER3 | Period Circadian Regulator 3 | Protein Coding | 43 | GC01P007785 |
| HTR2B | 5-Hydroxytryptamine Receptor 2B | Protein Coding | 43 | GC02M231108 |
| CSN1S1 | Casein Alpha S1 | Protein Coding | 34 | GC04P069932 |
| DNAJA3 | DnaJ Heat Shock Protein Family (Hsp40) Member A3 | Protein Coding | 40 | GC16P004425 |
| EFHC2 | EF-Hand Domain Containing 2 | Protein Coding | 35 | GC0XM044146 |
| PAPOLG | Poly(A) Polymerase Gamma | Protein Coding | 39 | GC02P060756 |
| LIF | LIF Interleukin 6 Family Cytokine | Protein Coding | 43 | GC22M030240 |
| JAM3 | Junctional Adhesion Molecule 3 | Protein Coding | 43 | GC11P134068 |
| MUL1 | Mitochondrial E3 Ubiquitin Protein Ligase 1 | Protein Coding | 38 | GC01M020499 |
| STAT5A | Signal Transducer And Activator Of Transcription 5A | Protein Coding | 45 | GC17P042287 |
| DAXX | Death Domain Associated Protein | Protein Coding | 44 | GC06M033318 |
| INHA | Inhibin Subunit Alpha | Protein Coding | 43 | GC02P219569 |
| ASB14 | Ankyrin Repeat And SOCS Box Containing 14 | Protein Coding | 32 | GC03M057278 |
| MLYCD | Malonyl-CoA Decarboxylase | Protein Coding | 43 | GC16P083899 |
| FYN | FYN Proto-Oncogene, Src Family Tyrosine Kinase | Protein Coding | 48 | GC06M111660 |
| RUNX1T1 | RUNX1 Partner Transcriptional Co-Repressor 1 | Protein Coding | 39 | GC08M091954 |
| PPP1CA | Protein Phosphatase 1 Catalytic Subunit Alpha | Protein Coding | 48 | GC11M067415 |
| SENP8 | SUMO Peptidase Family Member, NEDD8 Specific | Protein Coding | 39 | GC15P072938 |
| TLE1 | TLE Family Member 1, Transcriptional Corepressor | Protein Coding | 44 | GC09M081583 |
| KCNIP2 | Potassium Voltage-Gated Channel Interacting Protein 2 | Protein Coding | 40 | GC10M101825 |
| RAB3GAP1 | RAB3 GTPase Activating Protein Catalytic Subunit 1 | Protein Coding | 40 | GC02P135052 |
| GYG1 | Glycogenin 1 | Protein Coding | 45 | GC03P148991 |
| RAB11A | RAB11A, Member RAS Oncogene Family | Protein Coding | 46 | GC15P072880 |
| NDUFB6 | NADH:Ubiquinone Oxidoreductase Subunit B6 | Protein Coding | 40 | GC09M032553 |
| VRK2 | VRK Serine/Threonine Kinase 2 | Protein Coding | 41 | GC02P057907 |
| CA3 | Carbonic Anhydrase 3 | Protein Coding | 41 | GC08P085373 |
| STN1 | STN1 Subunit Of CST Complex | Protein Coding | 32 | GC10M103878 |
| NSUN5 | NOP2/Sun RNA Methyltransferase 5 | Protein Coding | 38 | GC07M073302 |
| PTGDR | Prostaglandin D2 Receptor | Protein Coding | 47 | GC14P052267 |
| CSPG5 | Chondroitin Sulfate Proteoglycan 5 | Protein Coding | 38 | GC03M047562 |
| A2M | Alpha-2-Macroglobulin | Protein Coding | 45 | GC12M009067 |
| LTA4H | Leukotriene A4 Hydrolase | Protein Coding | 46 | GC12M096000 |
| LRRC39 | Leucine Rich Repeat Containing 39 | Protein Coding | 35 | GC01M100148 |
| STAT2 | Signal Transducer And Activator Of Transcription 2 | Protein Coding | 47 | GC12M056341 |
| PNMT | Phenylethanolamine N-Methyltransferase | Protein Coding | 44 | GC17P039667 |
| CHRFAM7A | CHRNA7 (Exons 5-10) And FAM7A (Exons A-E) Fusion | Protein Coding | 32 | GC15M030360 |
| CUL2 | Cullin 2 | Protein Coding | 42 | GC10M035046 |
| MIR455 | MicroRNA 455 | RNA Gene | 18 | GC09P114209 |
| CLPB | Caseinolytic Mitochondrial Matrix Peptidase Chaperone Subunit B | Protein Coding | 43 | GC11M072292 |
| SLC35C2 | Solute Carrier Family 35 Member C2 | Protein Coding | 37 | GC20M046345 |
| AHCY | Adenosylhomocysteinase | Protein Coding | 50 | GC20M034276 |
| ACYP2 | Acylphosphatase 2 | Protein Coding | 40 | GC02P053970 |
| ACTN1 | Actinin Alpha 1 | Protein Coding | 50 | GC14M068874 |
| CYP11A1 | Cytochrome P450 Family 11 Subfamily A Member 1 | Protein Coding | 48 | GC15M074337 |
| PIGW | Phosphatidylinositol Glycan Anchor Biosynthesis Class W | Protein Coding | 37 | GC17P036534 |
| HPSE | Heparanase | Protein Coding | 44 | GC04M083292 |
| MAS1L | MAS1 Proto-Oncogene Like, G Protein-Coupled Receptor | Protein Coding | 35 | GC06M029504 |
| RHOF | Ras Homolog Family Member F, Filopodia Associated | Protein Coding | 37 | GC12M121777 |
| TIMM17B | Translocase Of Inner Mitochondrial Membrane 17B | Protein Coding | 37 | GC0XM048893 |
| ZBTB38 | Zinc Finger And BTB Domain Containing 38 | Protein Coding | 36 | GC03P141324 |
| SYNM | Synemin | Protein Coding | 37 | GC15P099098 |
| EIF4H | Eukaryotic Translation Initiation Factor 4H | Protein Coding | 41 | GC07P074174 |
| XDH | Xanthine Dehydrogenase | Protein Coding | 47 | GC02M031294 |
| NSUN7 | NOP2/Sun RNA Methyltransferase Family Member 7 | Protein Coding | 34 | GC04P040751 |
| MCU | Mitochondrial Calcium Uniporter | Protein Coding | 36 | GC10P072692 |
| TRAPPC10 | Trafficking Protein Particle Complex 10 | Protein Coding | 37 | GC21P044012 |
| MCL1 | MCL1 Apoptosis Regulator, BCL2 Family Member | Protein Coding | 47 | GC01M150707 |
| RXRB | Retinoid X Receptor Beta | Protein Coding | 47 | GC06M033193 |
| ZAP70 | Zeta Chain Of T Cell Receptor Associated Protein Kinase 70 | Protein Coding | 51 | GC02P097696 |
| FOXO3 | Forkhead Box O3 | Protein Coding | 44 | GC06P108559 |
| CPA6 | Carboxypeptidase A6 | Protein Coding | 43 | GC08M067422 |
| CYP11B1 | Cytochrome P450 Family 11 Subfamily B Member 1 | Protein Coding | 48 | GC08M142872 |
| NDST1 | N-Deacetylase And N-Sulfotransferase 1 | Protein Coding | 45 | GC05P150484 |
| PGC | Progastricsin | Protein Coding | 42 | GC06M041736 |
| LOC107303338 | 3p25 FANCD2 Alu-Mediated Recombination Region | Biological Region | 1 | GC03P010048 |
| ANGPTL6 | Angiopoietin Like 6 | Protein Coding | 39 | GC19M010092 |
| CDH15 | Cadherin 15 | Protein Coding | 43 | GC16P089171 |
| SLPI | Secretory Leukocyte Peptidase Inhibitor | Protein Coding | 39 | GC20M045252 |
| PICALM | Phosphatidylinositol Binding Clathrin Assembly Protein | Protein Coding | 43 | GC11M085957 |
| SREBF2 | Sterol Regulatory Element Binding Transcription Factor 2 | Protein Coding | 43 | GC22P041833 |
| NFATC4 | Nuclear Factor Of Activated T Cells 4 | Protein Coding | 44 | GC14P024365 |
| NSF | N-Ethylmaleimide Sensitive Factor, Vesicle Fusing ATPase | Protein Coding | 44 | GC17P046590 |
| FGB | Fibrinogen Beta Chain | Protein Coding | 45 | GC04P154564 |
| USP30 | Ubiquitin Specific Peptidase 30 | Protein Coding | 39 | GC12P109027 |
| FAM199X | Family With Sequence Similarity 199, X-Linked | Protein Coding | 30 | GC0XP104166 |
| GALNT17 | Polypeptide N-Acetylgalactosaminyltransferase 17 | Protein Coding | 31 | GC07P071133 |
| VAMP2 | Vesicle Associated Membrane Protein 2 | Protein Coding | 43 | GC17M008854 |
| HNRNPH2 | Heterogeneous Nuclear Ribonucleoprotein H2 | Protein Coding | 37 | GC0XP101408 |
| REXO2 | RNA Exonuclease 2 | Protein Coding | 37 | GC11P114439 |
| ABHD11-AS1 | ABHD11 Antisense RNA 1 (Tail To Tail) | RNA Gene | 16 | GC07P073735 |
| CYP17A1 | Cytochrome P450 Family 17 Subfamily A Member 1 | Protein Coding | 48 | GC10M102830 |
| CAP2 | Cyclase Associated Actin Cytoskeleton Regulatory Protein 2 | Protein Coding | 40 | GC06P017393 |
| RND1 | Rho Family GTPase 1 | Protein Coding | 38 | GC12M048857 |
| NR1H2 | Nuclear Receptor Subfamily 1 Group H Member 2 | Protein Coding | 48 | GC19P050329 |
| LAS1L | LAS1 Like Ribosome Biogenesis Factor | Protein Coding | 37 | GC0XM065512 |
| RNASEL | Ribonuclease L | Protein Coding | 44 | GC01M182542 |
| SEC63 | SEC63 Homolog, Protein Translocation Regulator | Protein Coding | 43 | GC06M107867 |
| MMUT | Methylmalonyl-CoA Mutase | Protein Coding | 35 | GC06M049430 |
| SHOX2 | Short Stature Homeobox 2 | Protein Coding | 39 | GC03M158095 |
| STRADB | STE20 Related Adaptor Beta | Protein Coding | 39 | GC02P201387 |
| HNMT | Histamine N-Methyltransferase | Protein Coding | 45 | GC02P137964 |
| SAMD12 | Sterile Alpha Motif Domain Containing 12 | Protein Coding | 37 | GC08M118131 |
| SLC38A3 | Solute Carrier Family 38 Member 3 | Protein Coding | 39 | GC03P050205 |
| RPS6KA2 | Ribosomal Protein S6 Kinase A2 | Protein Coding | 46 | GC06M166409 |
| FST | Follistatin | Protein Coding | 46 | GC05P053480 |
| TLX2 | T Cell Leukemia Homeobox 2 | Protein Coding | 36 | GC02P074514 |
| PMCH | Pro-Melanin Concentrating Hormone | Protein Coding | 36 | GC12M102196 |
| MIOX | Myo-Inositol Oxygenase | Protein Coding | 39 | GC22P050486 |
| SH2D3A | SH2 Domain Containing 3A | Protein Coding | 35 | GC19M006752 |
| HDAC3 | Histone Deacetylase 3 | Protein Coding | 49 | GC05M141583 |
| ABHD11 | Abhydrolase Domain Containing 11 | Protein Coding | 36 | GC07M073736 |
| MCM6 | Minichromosome Maintenance Complex Component 6 | Protein Coding | 43 | GC02M135839 |
| MARVELD3 | MARVEL Domain Containing 3 | Protein Coding | 34 | GC16P071626 |
| STT3B | STT3 Oligosaccharyltransferase Complex Catalytic Subunit B | Protein Coding | 44 | GC03P031550 |
| MIR379 | MicroRNA 379 | RNA Gene | 16 | GC14P104795 |
| FKBP6 | FKBP Prolyl Isomerase 6 | Protein Coding | 39 | GC07P073328 |
| PHYHIP | Phytanoyl-CoA 2-Hydroxylase Interacting Protein | Protein Coding | 35 | GC08M022219 |
| PRSS57 | Serine Protease 57 | Protein Coding | 31 | GC19M000686 |
| VIPR1 | Vasoactive Intestinal Peptide Receptor 1 | Protein Coding | 45 | GC03P042490 |
| ABCE1 | ATP Binding Cassette Subfamily E Member 1 | Protein Coding | 38 | GC04P145097 |
| GTF2IRD2 | GTF2I Repeat Domain Containing 2 | Protein Coding | 32 | GC07M074796 |
| KLHL40 | Kelch Like Family Member 40 | Protein Coding | 37 | GC03P042685 |
| CYP7A1 | Cytochrome P450 Family 7 Subfamily A Member 1 | Protein Coding | 42 | GC08M058476 |
| ELOB | Elongin B | Protein Coding | 32 | GC16M002772 |
| CGA | Glycoprotein Hormones, Alpha Polypeptide | Protein Coding | 43 | GC06M087085 |
| FAT1 | FAT Atypical Cadherin 1 | Protein Coding | 39 | GC04M186587 |
| TLX3 | T Cell Leukemia Homeobox 3 | Protein Coding | 38 | GC05P171309 |
| TBX2 | T-Box Transcription Factor 2 | Protein Coding | 45 | GC17P061399 |
| TSHB | Thyroid Stimulating Hormone Subunit Beta | Protein Coding | 42 | GC01P115029 |
| PPP1R13L | Protein Phosphatase 1 Regulatory Subunit 13 Like | Protein Coding | 39 | GC19M045379 |
| QSOX2 | Quiescin Sulfhydryl Oxidase 2 | Protein Coding | 36 | GC09M136206 |
| BCL2L11 | BCL2 Like 11 | Protein Coding | 45 | GC02P111119 |
| UCA1 | Urothelial Cancer Associated 1 | RNA Gene | 24 | GC19P015828 |
| CHD1 | Chromodomain Helicase DNA Binding Protein 1 | Protein Coding | 45 | GC05M098853 |
| MYL4 | Myosin Light Chain 4 | Protein Coding | 44 | GC17P047189 |
| MXD4 | MAX Dimerization Protein 4 | Protein Coding | 36 | GC04M002295 |
| TMEM233 | Transmembrane Protein 233 | Protein Coding | 27 | GC12P119594 |
| SLC18A1 | Solute Carrier Family 18 Member A1 | Protein Coding | 45 | GC08M020144 |
| HBG2 | Hemoglobin Subunit Gamma 2 | Protein Coding | 42 | GC11M005372 |
| TTC7A | Tetratricopeptide Repeat Domain 7A | Protein Coding | 38 | GC02P046906 |
| NPL | N-Acetylneuraminate Pyruvate Lyase | Protein Coding | 39 | GC01P182758 |
| STAG3L2 | Stromal Antigen 3-Like 2 (Pseudogene) | Pseudogene | 21 | GC07M074843 |
| LOC106029312 | Williams-Beuren Syndrome Medial Block B Recombination Region | Biological Region | 1 | GC07P074733 |
| PRKAB1 | Protein Kinase AMP-Activated Non-Catalytic Subunit Beta 1 | Protein Coding | 47 | GC12P119632 |
| RPS6KA1 | Ribosomal Protein S6 Kinase A1 | Protein Coding | 50 | GC01P026540 |
| SH2D3C | SH2 Domain Containing 3C | Protein Coding | 39 | GC09M127738 |
| PRKCSH | Protein Kinase C Substrate 80K-H | Protein Coding | 43 | GC19P011435 |
| NISCH | Nischarin | Protein Coding | 41 | GC03P052455 |
| ACTN3 | Actinin Alpha 3 | Protein Coding | 39 | GC11P066546 |
| DLD | Dihydrolipoamide Dehydrogenase | Protein Coding | 50 | GC07P107890 |
| FRZB | Frizzled Related Protein | Protein Coding | 41 | GC02M182833 |
| GLYAT | Glycine-N-Acyltransferase | Protein Coding | 41 | GC11M061239 |
| CSF2RA | Colony Stimulating Factor 2 Receptor Subunit Alpha | Protein Coding | 45 | GC0XP001270 |
| PDLIM5 | PDZ And LIM Domain 5 | Protein Coding | 39 | GC04P094451 |
| SLC1A7 | Solute Carrier Family 1 Member 7 | Protein Coding | 43 | GC01M053087 |
| NIF3L1 | NGG1 Interacting Factor 3 Like 1 | Protein Coding | 39 | GC02P200889 |
| BCL7B | BAF Chromatin Remodeling Complex Subunit BCL7B | Protein Coding | 36 | GC07M073536 |
| LIMK2 | LIM Domain Kinase 2 | Protein Coding | 47 | GC22P031212 |
| ANK1 | Ankyrin 1 | Protein Coding | 43 | GC08M041653 |
| CACNA2D3 | Calcium Voltage-Gated Channel Auxiliary Subunit Alpha2delta 3 | Protein Coding | 40 | GC03P054156 |
| FLRT1 | Fibronectin Leucine Rich Transmembrane Protein 1 | Protein Coding | 40 | GC11P064036 |
| ATP5F1E | ATP Synthase F1 Subunit Epsilon | Protein Coding | 32 | GC20M059026 |
| COX7C | Cytochrome C Oxidase Subunit 7C | Protein Coding | 38 | GC05P086617 |
| IL6R | Interleukin 6 Receptor | Protein Coding | 48 | GC01P154405 |
| ENTPD2 | Ectonucleoside Triphosphate Diphosphohydrolase 2 | Protein Coding | 39 | GC09M137048 |
| MXD1 | MAX Dimerization Protein 1 | Protein Coding | 37 | GC02P069897 |
| TRIM74 | Tripartite Motif Containing 74 | Protein Coding | 29 | GC07M072954 |
| NSUN5P2 | NSUN5 Pseudogene 2 | Pseudogene | 24 | GC07M072948 |
| TRIM31 | Tripartite Motif Containing 31 | Protein Coding | 37 | GC06M030871 |
| POM121C | POM121 Transmembrane Nucleoporin C | Protein Coding | 31 | GC07M075416 |
| GTF2IP1 | General Transcription Factor IIi Pseudogene 1 | Pseudogene | 13 | GC07M075185 |
| ELOC | Elongin C | Protein Coding | 33 | GC08M073939 |
| OXA1L | OXA1L Mitochondrial Inner Membrane Protein | Protein Coding | 39 | GC14P022766 |
| FOSL1 | FOS Like 1, AP-1 Transcription Factor Subunit | Protein Coding | 44 | GC11M065909 |
| CCL26 | C-C Motif Chemokine Ligand 26 | Protein Coding | 37 | GC07M075769 |
| MHRT | Myosin Heavy Chain Associated RNA Transcript | RNA Gene | 10 | GC14P025365 |
| ADRA1B | Adrenoceptor Alpha 1B | Protein Coding | 46 | GC05P159867 |
| DEFA3 | Defensin Alpha 3 | Protein Coding | 36 | GC08M007015 |
| TUBG1 | Tubulin Gamma 1 | Protein Coding | 47 | GC17P042609 |
| LAT2 | Linker For Activation Of T Cells Family Member 2 | Protein Coding | 39 | GC07P074199 |
| DNAJC30 | DnaJ Heat Shock Protein Family (Hsp40) Member C30 | Protein Coding | 33 | GC07M073680 |
| TRIM50 | Tripartite Motif Containing 50 | Protein Coding | 32 | GC07M073312 |
| VPS37D | VPS37D Subunit Of ESCRT-I | Protein Coding | 32 | GC07P073666 |
| GTF2IRD2B | GTF2I Repeat Domain Containing 2B | Protein Coding | 30 | GC07P075092 |
| LOC108228208 | 7q11.23 Proximal Recombination Region | Biological Region | 1 | GC07P075442 |
| LOC108228209 | 7q11.23 Distal Recombination Region | Biological Region | 1 | GC07P076626 |
| SPDYE1 | Speedy/RINGO Cell Cycle Regulator Family Member E1 | Protein Coding | 29 | GC07P043998 |
| TRIM73 | Tripartite Motif Containing 73 | Protein Coding | 28 | GC07P075395 |
| METTL27 | Methyltransferase Like 27 | Protein Coding | 25 | GC07M073835 |
| STAG3L1 | Stromal Antigen 3-Like 1 (Pseudogene) | Pseudogene | 24 | GC07P075359 |
| STAG3L4 | Stromal Antigen 3-Like 4 (Pseudogene) | Pseudogene | 23 | GC07P067302 |
| TMEM270 | Transmembrane Protein 270 | Protein Coding | 23 | GC07P073862 |
| NSUN5P1 | NSUN5 Pseudogene 1 | Pseudogene | 20 | GC07P075410 |
| STAG3L3 | Stromal Antigen 3-Like 3 (Pseudogene) | Pseudogene | 19 | GC07M072969 |
| SPDYE7P | Speedy/RINGO Cell Cycle Regulator Family Member E7, Pseudogene | Protein Coding | 18 | GC07M072862 |
| LINC00851 | Long Intergenic Non-Protein Coding RNA 851 | RNA Gene | 16 | GC20P018378 |
| GTF2IRD2P1 | GTF2I Repeat Domain Containing 2 Pseudogene 1 | Pseudogene | 12 | GC07M073242 |
| SPDYE8 | Speedy/RINGO Cell Cycle Regulator Family Member E8 | Protein Coding | 10 | GC07M073022 |
| GTF2IP4 | General Transcription Factor IIi Pseudogene 4 | Pseudogene | 9 | GC07P073206 |
| SPDYE13 | Speedy/RINGO Cell Cycle Regulator Family Member E13 | Protein Coding | 9 | GC07P075284 |
| SPDYE12P | Speedy/RINGO Cell Cycle Regulator Family Member E12, Pseudogene | Protein Coding | 8 | GC07M074904 |
| SPDYE15 | Speedy/RINGO Cell Cycle Regulator Family Member E15 | Protein Coding | 8 | GC07P075335 |
| SPDYE14 | Speedy/RINGO Cell Cycle Regulator Family Member E14 | Protein Coding | 7 | GC07P075300 |
| EIF4HP1 | Eukaryotic Translation Initiation Factor 4H Pseudogene 1 | Pseudogene | 6 | GC07M027456 |
| SPDYE10P | Speedy/RINGO Cell Cycle Regulator Family Member E10, Pseudogene | Protein Coding | 6 | GC07M073104 |
| SPDYE9 | Speedy/RINGO Cell Cycle Regulator Family Member E9 | Protein Coding | 6 | GC07M073078 |
| WBSCR2 | Williams-Beuren Syndrome Chromosome Region 2 | Uncategorized | 5 | GC07U990122 |
| WBSCR23 | Williams-Beuren Syndrome Chromosome Region 23 | RNA Gene | 5 | GC07P074532 |
| LOC106029311 | Williams-Beuren Syndrome Centromeric Block B Recombination Region | Biological Region | 1 | GC07P073209 |
| LOC106029313 | Williams-Beuren Syndrome Telomeric Block B Recombination Region | Biological Region | 1 | GC07P075074 |
| HNRNPH1 | Heterogeneous Nuclear Ribonucleoprotein H1 | Protein Coding | 38 | GC05M179614 |
| PGAP1 | Post-GPI Attachment To Proteins Inositol Deacylase 1 | Protein Coding | 40 | GC02M196833 |
| COX5B | Cytochrome C Oxidase Subunit 5B | Protein Coding | 41 | GC02P097628 |
| ATP13A3 | ATPase 13A3 | Protein Coding | 37 | GC03M194402 |
| PEMT | Phosphatidylethanolamine N-Methyltransferase | Protein Coding | 41 | GC17M017506 |
| LOC107303337 | 3p25 PRRT3 Alu-Mediated Recombination Region | Biological Region | 1 | GC03P009972 |
| LOC107303339 | 3p25 BRK1 Alu-Mediated Recombination Region | Biological Region | 1 | GC03P010120 |
| LOC107303340 | 3p25 Von Hippel-Lindau Tumor Suppressor, E3 Ubiquitin Protein Ligase Alu-Mediated Recombination Region | Biological Region | 1 | GC03P010215 |
| LOC107303341 | 3p25 IRAK2 Alu-Mediated Recombination Region | Biological Region | 1 | GC03P010216 |
| TRPM3 | Transient Receptor Potential Cation Channel Subfamily M Member 3 | Protein Coding | 41 | GC09M070529 |
| HES1 | Hes Family BHLH Transcription Factor 1 | Protein Coding | 43 | GC03P194136 |
| MRPL18 | Mitochondrial Ribosomal Protein L18 | Protein Coding | 36 | GC06P159789 |
| CRY1 | Cryptochrome Circadian Regulator 1 | Protein Coding | 43 | GC12M106991 |
| ARRDC4 | Arrestin Domain Containing 4 | Protein Coding | 34 | GC15P097960 |
| YRDC | YrdC N6-Threonylcarbamoyltransferase Domain Containing | Protein Coding | 32 | GC01M037802 |
| GGA2 | Golgi Associated, Gamma Adaptin Ear Containing, ARF Binding Protein 2 | Protein Coding | 39 | GC16M023464 |
| OSGEPL1 | O-Sialoglycoprotein Endopeptidase Like 1 | Protein Coding | 36 | GC02M189746 |
| ZFYVE21 | Zinc Finger FYVE-Type Containing 21 | Protein Coding | 36 | GC14P103715 |
| CDK5RAP1 | CDK5 Regulatory Subunit Associated Protein 1 | Protein Coding | 37 | GC20M033358 |
| GPR22 | G Protein-Coupled Receptor 22 | Protein Coding | 35 | GC07P107470 |
| OR4L1 | Olfactory Receptor Family 4 Subfamily L Member 1 | Protein Coding | 33 | GC14P020060 |
| SNORD35A | Small Nucleolar RNA, C/D Box 35A | RNA Gene | 16 | GC19P049631 |
| TRL-AAG2-3 | TRNA-Leu (Anticodon AAG) 2-3 | RNA Gene | 10 | GC14P020612 |
| ANKRD49 | Ankyrin Repeat Domain 49 | Protein Coding | 36 | GC11P094493 |
| PLCE1 | Phospholipase C Epsilon 1 | Protein Coding | 44 | GC10P093993 |
| GJD2 | Gap Junction Protein Delta 2 | Protein Coding | 41 | GC15M034751 |
| CD163 | CD163 Molecule | Protein Coding | 42 | GC12M007471 |
| CGAS | Cyclic GMP-AMP Synthase | Protein Coding | 29 | GC06M073414 |
| SLC30A6 | Solute Carrier Family 30 Member 6 | Protein Coding | 38 | GC02P032166 |
| TYK2 | Tyrosine Kinase 2 | Protein Coding | 52 | GC19M010350 |
| ZMYM2 | Zinc Finger MYM-Type Containing 2 | Protein Coding | 39 | GC13P019958 |
| LOC107303342 | 3p25 TatD DNase Domain Containing 2 Alu-Mediated Recombination Region | Biological Region | 1 | GC03P010822 |
| LOC113939944 | Sharpr-MPRA Regulatory Region 9539 | Biological Region | 1 | GC15P048520 |
| ANKRD26 | Ankyrin Repeat Domain 26 | Protein Coding | 39 | GC10M026938 |
| HSPA1L | Heat Shock Protein Family A (Hsp70) Member 1 Like | Protein Coding | 43 | GC06M031809 |
| ORAI1 | ORAI Calcium Release-Activated Calcium Modulator 1 | Protein Coding | 44 | GC12P122835 |
| PAPPA | Pappalysin 1 | Protein Coding | 41 | GC09P116179 |
| ROCK1 | Rho Associated Coiled-Coil Containing Protein Kinase 1 | Protein Coding | 50 | GC18M020946 |
| IFI44L | Interferon Induced Protein 44 Like | Protein Coding | 33 | GC01P078619 |
| MYOG | Myogenin | Protein Coding | 39 | GC01M203083 |
| FNDC3B | Fibronectin Type III Domain Containing 3B | Protein Coding | 36 | GC03P172039 |
| IL4R | Interleukin 4 Receptor | Protein Coding | 47 | GC16P027325 |
| PRKACG | Protein Kinase CAMP-Activated Catalytic Subunit Gamma | Protein Coding | 47 | GC09M069013 |
| MUSK | Muscle Associated Receptor Tyrosine Kinase | Protein Coding | 46 | GC09P110668 |
| GHRHR | Growth Hormone Releasing Hormone Receptor | Protein Coding | 43 | GC07P030978 |
| GET3 | Guided Entry Of Tail-Anchored Proteins Factor 3, ATPase | Protein Coding | 33 | GC19P012737 |
| STX16 | Syntaxin 16 | Protein Coding | 42 | GC20P058652 |
| GADD45A | Growth Arrest And DNA Damage Inducible Alpha | Protein Coding | 44 | GC01P067685 |
| PI4KA | Phosphatidylinositol 4-Kinase Alpha | Protein Coding | 48 | GC22M020707 |
| PRKCZ | Protein Kinase C Zeta | Protein Coding | 48 | GC01P002050 |
| TPM4 | Tropomyosin 4 | Protein Coding | 40 | GC19P023264 |
| MIR383 | MicroRNA 383 | RNA Gene | 15 | GC08M014853 |
| FAS-AS1 | FAS Antisense RNA 1 | RNA Gene | 14 | GC10M088991 |
| CAMTA1 | Calmodulin Binding Transcription Activator 1 | Protein Coding | 40 | GC01P006845 |
| SAR1A | Secretion Associated Ras Related GTPase 1A | Protein Coding | 41 | GC10M070147 |
| UPK1A | Uroplakin 1A | Protein Coding | 37 | GC19P038225 |
| HLA-DMA | Major Histocompatibility Complex, Class II, DM Alpha | Protein Coding | 40 | GC06M032950 |
| FAM126A | Family With Sequence Similarity 126 Member A | Protein Coding | 37 | GC07M022889 |
| STARD13 | StAR Related Lipid Transfer Domain Containing 13 | Protein Coding | 39 | GC13M033103 |
| FLVCR2 | FLVCR Heme Transporter 2 | Protein Coding | 41 | GC14P075578 |
| UBE2K | Ubiquitin Conjugating Enzyme E2 K | Protein Coding | 41 | GC04P039700 |
| POFUT2 | Protein O-Fucosyltransferase 2 | Protein Coding | 38 | GC21M045263 |
| FSCN1 | Fascin Actin-Bundling Protein 1 | Protein Coding | 43 | GC07P005592 |
| DPM3 | Dolichyl-Phosphate Mannosyltransferase Subunit 3, Regulatory | Protein Coding | 40 | GC01M155112 |
| KCNK9 | Potassium Two Pore Domain Channel Subfamily K Member 9 | Protein Coding | 46 | GC08M139585 |
| COA4 | Cytochrome C Oxidase Assembly Factor 4 Homolog | Protein Coding | 33 | GC11M073872 |
| SETD1B | SET Domain Containing 1B, Histone Lysine Methyltransferase | Protein Coding | 35 | GC12P122842 |
| UBAC1 | UBA Domain Containing 1 | Protein Coding | 36 | GC09M135932 |
| SMAD1 | SMAD Family Member 1 | Protein Coding | 44 | GC04P145481 |
| SEC22B | SEC22 Homolog B, Vesicle Trafficking Protein | Protein Coding | 35 | GC01M120150 |
| PTBP1 | Polypyrimidine Tract Binding Protein 1 | Protein Coding | 41 | GC19P000797 |
| DEGS2 | Delta 4-Desaturase, Sphingolipid 2 | Protein Coding | 38 | GC14M100143 |
| PTK7 | Protein Tyrosine Kinase 7 (Inactive) | Protein Coding | 43 | GC06P043076 |
| GNAI2 | G Protein Subunit Alpha I2 | Protein Coding | 47 | GC03P050226 |
| LDHD | Lactate Dehydrogenase D | Protein Coding | 40 | GC16M075111 |
| ALAS2 | 5'-Aminolevulinate Synthase 2 | Protein Coding | 45 | GC0XM055009 |
| FADS1 | Fatty Acid Desaturase 1 | Protein Coding | 44 | GC11M061799 |
| PIGM | Phosphatidylinositol Glycan Anchor Biosynthesis Class M | Protein Coding | 37 | GC01M160027 |
| COQ3 | Coenzyme Q3, Methyltransferase | Protein Coding | 40 | GC06M099369 |
| SNORD94 | Small Nucleolar RNA, C/D Box 94 | RNA Gene | 16 | GC02P086136 |
| AGA | Aspartylglucosaminidase | Protein Coding | 45 | GC04M177430 |
| SYTL4 | Synaptotagmin Like 4 | Protein Coding | 39 | GC0XM100674 |
| PDE3A | Phosphodiesterase 3A | Protein Coding | 48 | GC12P020294 |
| TIAM2 | TIAM Rac1 Associated GEF 2 | Protein Coding | 40 | GC06P154832 |
| C6orf15 | Chromosome 6 Open Reading Frame 15 | Protein Coding | 33 | GC06M031111 |
| TAMM41 | TAM41 Mitochondrial Translocator Assembly And Maintenance Homolog | Protein Coding | 32 | GC03M011721 |
| SLC47A1 | Solute Carrier Family 47 Member 1 | Protein Coding | 41 | GC17P019495 |
| UTRN | Utrophin | Protein Coding | 40 | GC06P144285 |
| MT-TG | Mitochondrially Encoded TRNA-Gly (GGN) | RNA Gene | 12 | GCMTP009993 |
| SAA1 | Serum Amyloid A1 | Protein Coding | 42 | GC11P018267 |
| SLC35E1 | Solute Carrier Family 35 Member E1 | Protein Coding | 35 | GC19M016549 |
| RGS6 | Regulator Of G Protein Signaling 6 | Protein Coding | 40 | GC14P071867 |
| HPCA | Hippocalcin | Protein Coding | 41 | GC01P032885 |
| LINC01554 | Long Intergenic Non-Protein Coding RNA 1554 | RNA Gene | 21 | GC05P095838 |
| CASC15 | Cancer Susceptibility 15 | RNA Gene | 18 | GC06P021669 |
| ADAM8 | ADAM Metallopeptidase Domain 8 | Protein Coding | 42 | GC10M133262 |
| TXNDC15 | Thioredoxin Domain Containing 15 | Protein Coding | 37 | GC05P134873 |
| OR1L6 | Olfactory Receptor Family 1 Subfamily L Member 6 | Protein Coding | 30 | GC09P122750 |
| HSP90B1 | Heat Shock Protein 90 Beta Family Member 1 | Protein Coding | 45 | GC12P103930 |
| PTMS | Parathymosin | Protein Coding | 37 | GC12P006765 |
| DPPA3 | Developmental Pluripotency Associated 3 | Protein Coding | 32 | GC12P008252 |
| HIKESHI | Heat Shock Protein Nuclear Import Factor Hikeshi | Protein Coding | 32 | GC11P086303 |
| B4GALNT1 | Beta-1,4-N-Acetyl-Galactosaminyltransferase 1 | Protein Coding | 44 | GC12M057623 |
| CCNB1 | Cyclin B1 | Protein Coding | 47 | GC05P069167 |
| PRIMA1 | Proline Rich Membrane Anchor 1 | Protein Coding | 32 | GC14M093718 |
| MCCC1 | Methylcrotonoyl-CoA Carboxylase 1 | Protein Coding | 44 | GC03M183015 |
| FCRL3 | Fc Receptor Like 3 | Protein Coding | 36 | GC01M157674 |
| MIR22HG | MIR22 Host Gene | RNA Gene | 27 | GC17M001714 |
| SP3 | Sp3 Transcription Factor | Protein Coding | 42 | GC02M173882 |
| GAS6 | Growth Arrest Specific 6 | Protein Coding | 44 | GC13M113820 |
| IGHM | Immunoglobulin Heavy Constant Mu | Protein Coding | 31 | GC14M109532 |
| RGPD2 | RANBP2 Like And GRIP Domain Containing 2 | Protein Coding | 30 | GC02M087755 |
| TCEA3 | Transcription Elongation Factor A3 | Protein Coding | 36 | GC01M023382 |
| PRDX5 | Peroxiredoxin 5 | Protein Coding | 46 | GC11P064317 |
| BIRC2 | Baculoviral IAP Repeat Containing 2 | Protein Coding | 45 | GC11P102347 |
| HSPA1B | Heat Shock Protein Family A (Hsp70) Member 1B | Protein Coding | 40 | GC06P047325 |
| GALK2 | Galactokinase 2 | Protein Coding | 41 | GC15P049155 |
| SUB1 | SUB1 Regulator Of Transcription | Protein Coding | 37 | GC05P032533 |
| C5orf15 | Chromosome 5 Open Reading Frame 15 | Protein Coding | 33 | GC05M133955 |
| LPCAT1 | Lysophosphatidylcholine Acyltransferase 1 | Protein Coding | 36 | GC05M001456 |
| PRKAR2A | Protein Kinase CAMP-Dependent Type II Regulatory Subunit Alpha | Protein Coding | 46 | GC03M048744 |
| PANX1 | Pannexin 1 | Protein Coding | 43 | GC11P094128 |
| DMGDH | Dimethylglycine Dehydrogenase | Protein Coding | 42 | GC05M078997 |
| SRD5A1 | Steroid 5 Alpha-Reductase 1 | Protein Coding | 44 | GC05P006633 |
| FHL5 | Four And A Half LIM Domains 5 | Protein Coding | 37 | GC06P096562 |
| TOMM22 | Translocase Of Outer Mitochondrial Membrane 22 | Protein Coding | 36 | GC22P038681 |
| DNAJC4 | DnaJ Heat Shock Protein Family (Hsp40) Member C4 | Protein Coding | 32 | GC11P064230 |
| YWHAH | Tyrosine 3-Monooxygenase/Tryptophan 5-Monooxygenase Activation Protein Eta | Protein Coding | 47 | GC22P031944 |
| NME7 | NME/NM23 Family Member 7 | Protein Coding | 43 | GC01M169101 |
| DPAGT1 | Dolichyl-Phosphate N-Acetylglucosaminephosphotransferase 1 | Protein Coding | 45 | GC11M119096 |
| HMGCS2 | 3-Hydroxy-3-Methylglutaryl-CoA Synthase 2 | Protein Coding | 44 | GC01M119747 |
| BAZ2B | Bromodomain Adjacent To Zinc Finger Domain 2B | Protein Coding | 39 | GC02M159318 |
| KCNK5 | Potassium Two Pore Domain Channel Subfamily K Member 5 | Protein Coding | 37 | GC06M042248 |
| BAG2 | BAG Cochaperone 2 | Protein Coding | 40 | GC06P057172 |
| GP9 | Glycoprotein IX Platelet | Protein Coding | 46 | GC03P130615 |
| H1-5 | H1.5 Linker Histone, Cluster Member | Protein Coding | 32 | GC06M028364 |
| HOXB5 | Homeobox B5 | Protein Coding | 39 | GC17M048591 |
| SOX6 | SRY-Box Transcription Factor 6 | Protein Coding | 42 | GC11M015949 |
| CTNNA2 | Catenin Alpha 2 | Protein Coding | 43 | GC02P079185 |
| AKR1B1 | Aldo-Keto Reductase Family 1 Member B | Protein Coding | 47 | GC07M134442 |
| EMX2 | Empty Spiracles Homeobox 2 | Protein Coding | 43 | GC10P117542 |
| CABIN1 | Calcineurin Binding Protein 1 | Protein Coding | 41 | GC22P024011 |
| SLC30A4 | Solute Carrier Family 30 Member 4 | Protein Coding | 39 | GC15M045479 |
| TCFL5 | Transcription Factor Like 5 | Protein Coding | 36 | GC20M062841 |
| MIR92A1 | MicroRNA 92a-1 | RNA Gene | 19 | GC13P091431 |
| APOLD1 | Apolipoprotein L Domain Containing 1 | Protein Coding | 33 | GC12P012725 |
| PNPLA4 | Patatin Like Phospholipase Domain Containing 4 | Protein Coding | 37 | GC0XM007898 |
| PCSK1 | Proprotein Convertase Subtilisin/Kexin Type 1 | Protein Coding | 45 | GC05M096391 |
| TRMT44 | TRNA Methyltransferase 44 Homolog | Protein Coding | 32 | GC04P008439 |
| CBX5 | Chromobox 5 | Protein Coding | 44 | GC12M054230 |
| CHL1 | Cell Adhesion Molecule L1 Like | Protein Coding | 39 | GC03P000213 |
| ESM1 | Endothelial Cell Specific Molecule 1 | Protein Coding | 40 | GC05M054977 |
| CRELD1 | Cysteine Rich With EGF Like Domains 1 | Protein Coding | 39 | GC03P009960 |
| PVR | PVR Cell Adhesion Molecule | Protein Coding | 44 | GC19P044644 |
| SLC25A26 | Solute Carrier Family 25 Member 26 | Protein Coding | 38 | GC03P066120 |
| HSD17B12 | Hydroxysteroid 17-Beta Dehydrogenase 12 | Protein Coding | 40 | GC11P043636 |
| YEATS2 | YEATS Domain Containing 2 | Protein Coding | 36 | GC03P183698 |
| SNN | Stannin | Protein Coding | 33 | GC16P011669 |
| ATP8A1 | ATPase Phospholipid Transporting 8A1 | Protein Coding | 42 | GC04M042410 |
| MED23 | Mediator Complex Subunit 23 | Protein Coding | 41 | GC06M131573 |
| GLS2 | Glutaminase 2 | Protein Coding | 41 | GC12M056470 |
| EXTL3 | Exostosin Like Glycosyltransferase 3 | Protein Coding | 45 | GC08P028615 |
| ABCG4 | ATP Binding Cassette Subfamily G Member 4 | Protein Coding | 39 | GC11P119150 |
| OSM | Oncostatin M | Protein Coding | 43 | GC22M030262 |
| TNNI3K | TNNI3 Interacting Kinase | Protein Coding | 40 | GC01P074235 |
| HES5 | Hes Family BHLH Transcription Factor 5 | Protein Coding | 34 | GC01M002528 |
| WASHC4 | WASH Complex Subunit 4 | Protein Coding | 30 | GC12P105108 |
| TCP1 | T-Complex 1 | Protein Coding | 41 | GC06M159778 |
| CDCA7L | Cell Division Cycle Associated 7 Like | Protein Coding | 40 | GC07M021900 |
| POLL | DNA Polymerase Lambda | Protein Coding | 43 | GC10M101578 |
| KCNN2 | Potassium Calcium-Activated Channel Subfamily N Member 2 | Protein Coding | 43 | GC05P114058 |
| CES1 | Carboxylesterase 1 | Protein Coding | 46 | GC16M055836 |
| EN2 | Engrailed Homeobox 2 | Protein Coding | 40 | GC07P155459 |
| EVX1 | Even-Skipped Homeobox 1 | Protein Coding | 33 | GC07P027298 |
| LOXL2 | Lysyl Oxidase Like 2 | Protein Coding | 44 | GC08M023296 |
| PRDM6 | PR/SET Domain 6 | Protein Coding | 39 | GC05P123089 |
| PCDH8 | Protocadherin 8 | Protein Coding | 39 | GC13M052842 |
| POLK | DNA Polymerase Kappa | Protein Coding | 41 | GC05P075511 |
| UGDH | UDP-Glucose 6-Dehydrogenase | Protein Coding | 45 | GC04M039502 |
| SLC2A4 | Solute Carrier Family 2 Member 4 | Protein Coding | 45 | GC17P007295 |
| MINK1 | Misshapen Like Kinase 1 | Protein Coding | 41 | GC17P004833 |
| VTI1A | Vesicle Transport Through Interaction With T-SNAREs 1A | Protein Coding | 39 | GC10P112446 |
| NRP2 | Neuropilin 2 | Protein Coding | 43 | GC02P205681 |
| LNPEP | Leucyl And Cystinyl Aminopeptidase | Protein Coding | 45 | GC05P096935 |
| ACTR2 | Actin Related Protein 2 | Protein Coding | 43 | GC02P065227 |
| TANC1 | Tetratricopeptide Repeat, Ankyrin Repeat And Coiled-Coil Containing 1 | Protein Coding | 33 | GC02P158968 |
| CD3D | CD3d Molecule | Protein Coding | 47 | GC11M118338 |
| ITPA | Inosine Triphosphatase | Protein Coding | 47 | GC20P003189 |
| PRSS12 | Serine Protease 12 | Protein Coding | 40 | GC04M118280 |
| GBF1 | Golgi Brefeldin A Resistant Guanine Nucleotide Exchange Factor 1 | Protein Coding | 43 | GC10P102245 |
| BLNK | B Cell Linker | Protein Coding | 47 | GC10M096198 |
| ELP6 | Elongator Acetyltransferase Complex Subunit 6 | Protein Coding | 33 | GC03M047495 |
| OFCC1 | Orofacial Cleft 1 Candidate 1 | Protein Coding | 32 | GC06M009596 |
| SLC12A9 | Solute Carrier Family 12 Member 9 | Protein Coding | 37 | GC07P100826 |
| PPP1CC | Protein Phosphatase 1 Catalytic Subunit Gamma | Protein Coding | 45 | GC12M110709 |
| RFFL | Ring Finger And FYVE Like Domain Containing E3 Ubiquitin Protein Ligase | Protein Coding | 39 | GC17M035006 |
| SYT10 | Synaptotagmin 10 | Protein Coding | 34 | GC12M033374 |
| CLCN3 | Chloride Voltage-Gated Channel 3 | Protein Coding | 41 | GC04P169612 |
| GH-LCR | Growth Hormone Locus Control Region | Biological Region | 1 | GC17P063917 |
| SARS1 | Seryl-TRNA Synthetase 1 | Protein Coding | 35 | GC01P109214 |
| MARK3 | Microtubule Affinity Regulating Kinase 3 | Protein Coding | 48 | GC14P103385 |
| FTSJ3 | FtsJ RNA 2'-O-Methyltransferase 3 | Protein Coding | 36 | GC17M063819 |
| CHRNB3 | Cholinergic Receptor Nicotinic Beta 3 Subunit | Protein Coding | 41 | GC08P042697 |
| TAPBPL | TAP Binding Protein Like | Protein Coding | 35 | GC12P006451 |
| CHD1L | Chromodomain Helicase DNA Binding Protein 1 Like | Protein Coding | 41 | GC01P147190 |
| GNAZ | G Protein Subunit Alpha Z | Protein Coding | 43 | GC22P023070 |
| VASP | Vasodilator Stimulated Phosphoprotein | Protein Coding | 43 | GC19P045507 |
| DPT | Dermatopontin | Protein Coding | 37 | GC01M168664 |
| IQSEC1 | IQ Motif And Sec7 Domain ArfGEF 1 | Protein Coding | 42 | GC03M015774 |
| ZNF202 | Zinc Finger Protein 202 | Protein Coding | 39 | GC11M123724 |
| POLR2G | RNA Polymerase II Subunit G | Protein Coding | 38 | GC11P062762 |
| GPR4 | G Protein-Coupled Receptor 4 | Protein Coding | 39 | GC19M045589 |
| DBX1 | Developing Brain Homeobox 1 | Protein Coding | 33 | GC11M020177 |
| CMC4 | C-X9-C Motif Containing 4 | Protein Coding | 29 | GC0XM155061 |
| ASIC2 | Acid Sensing Ion Channel Subunit 2 | Protein Coding | 39 | GC17M033013 |
| MEF2A | Myocyte Enhancer Factor 2A | Protein Coding | 47 | GC15P099565 |
| DNAJA1 | DnaJ Heat Shock Protein Family (Hsp40) Member A1 | Protein Coding | 41 | GC09P033025 |
| OCM2 | Oncomodulin 2 | Protein Coding | 27 | GC07M097984 |
| STIP1 | Stress Induced Phosphoprotein 1 | Protein Coding | 43 | GC11P064203 |
| PTGER3 | Prostaglandin E Receptor 3 | Protein Coding | 46 | GC01M070852 |
| MEF2D | Myocyte Enhancer Factor 2D | Protein Coding | 43 | GC01M156463 |
| LOC108644431 | Myotonic Dystrophy Type 2 Repeat Instability Region | Biological Region | 1 | GC03P130680 |
| FDX2 | Ferredoxin 2 | Protein Coding | 28 | GC19M010311 |
| FBP2 | Fructose-Bisphosphatase 2 | Protein Coding | 43 | GC09M094558 |

The date for data search is March 2021.
