# Supplementary material for: Resveratrol Ameliorates Lipopolysaccharide-Induced Sudden Sensorineural Hearing Loss in In Vitro Model through Multitarget Antiapoptotic Mechanism Based on Network Pharmacology and Molecular Docking
Source: Evid Based Complement Alternat Med. 2022 May 19;2022:6404588. doi: 10.1155/2022/6404588 (PMC9135530; doi:10.1155/2022/6404588)
Supplement: Supplementary Materials — Table S1. Targets of resveratrol from the TCMSP database (151 resveratrol target proteins were obtained and transformed into corresponding differential genes). Table S2. Targets of SSNHL from the DisGeNET database (2342 corresponding target genes were obtained). Table S3. RSV-SSNHL potential targets and Cytoscape analysis results (a total of 2416 nodes were obtained by PPI analysis, and the number of nodes was selected as greater than or equal to 70). [file 6404588.f1.zip › 6404588.f1/Table S3 Cytoscape analysis result.docx]

Table S3 RSV-SSNHL potential targets Cytoscape analysis results

| **Gene name** | **Degree** | **Stress** |
| --- | --- | --- |
| AKT1 | 82 | 3234 |
| STAT3 | 80 | 2320 |
| JUN | 80 | 2552 |
| TNF | 78 | 3234 |
| TP53 | 76 | 2852 |
| MAPK3 | 72 | 1954 |
| CASP3 | 70 | 2244 |
| VEGFA | 70 | 1846 |
| IL6 | 68 | 2442 |
| MMP9 | 60 | 2434 |
| HIF1A | 60 | 1178 |
| MYC | 60 | 1586 |
| MAPK1 | 58 | 1186 |
| CTNNB1 | 58 | 1684 |
| IL1B | 54 | 1244 |
| CCND1 | 50 | 834 |
| FOS | 46 | 790 |
| FGF2 | 44 | 638 |
| PTEN | 42 | 490 |
| IL10 | 42 | 474 |
| PTGS2 | 40 | 452 |
| MAPK8 | 38 | 538 |
| CXCL8 | 38 | 512 |
| MTOR | 38 | 500 |
| TGFB1 | 38 | 356 |
| HGF | 36 | 570 |
| PPARG | 36 | 358 |
| BCL2L1 | 36 | 168 |
| STAT1 | 36 | 380 |
| EDN1 | 36 | 372 |
| CCL2 | 36 | 302 |
| SIRT1 | 34 | 400 |
| CDC42 | 34 | 458 |
| ICAM1 | 34 | 230 |
| MMP2 | 32 | 356 |
| CDKN1A | 30 | 116 |
| VCAM1 | 30 | 136 |
| NOS3 | 28 | 354 |
| CRP | 28 | 124 |
| CASP9 | 26 | 144 |
| BRCA1 | 26 | 132 |
| CDK4 | 26 | 158 |
| IL1A | 26 | 46 |
| CAT | 24 | 1228 |
| MCL1 | 24 | 40 |
| PRKCA | 24 | 196 |
| IGF1R | 24 | 150 |
| CCNB1 | 22 | 62 |
| PECAM1 | 22 | 198 |
| BCL2L11 | 20 | 20 |
| MPO | 20 | 1314 |
| SELE | 20 | 24 |
| CDK6 | 20 | 36 |
| BIRC5 | 18 | 32 |
| PPARA | 18 | 140 |
| PRKCB | 16 | 54 |
| NTRK1 | 16 | 18 |
| F3 | 12 | 60 |
| CD28 | 10 | 36 |
| SOD2 | 10 | 46 |
| BRCA2 | 10 | 0 |
| SOD1 | 8 | 18 |
| SLC2A4 | 8 | 10 |
| TNFSF10 | 8 | 2 |
| GJA1 | 8 | 4 |
| TGFB2 | 8 | 4 |
| PRKD1 | 8 | 20 |
| PLAU | 6 | 0 |
| ABCB1 | 4 | 0 |
| PLAT | 4 | 12 |
| PRKAG2 | 4 | 0 |
| CA2 | 2 | 0 |
| XDH | 2 | 0 |
| PON1 | 2 | 0 |
| ODC1 | 2 | 0 |

The genes whose names are in red font are the genes with the top 8 degree values.
